# Supplementary material for: Structural basis for RAD18 regulation by MAGEA4 and its implications for RING ubiquitin ligase binding by MAGE family proteins
Source: EMBO J. 2024 Mar 6;43(7):1273–300. doi: 10.1038/s44318-024-00058-9 (PMC10987633; doi:10.1038/s44318-024-00058-9)
Supplement: Supplementary file 1 — Appendix [file 44318_2024_58_MOESM1_ESM.pdf]

Appendix

Structural basis for RAD18 regulation by MAGEA4 and its implications for RING ubiquitin ligase binding by MAGE family proteins

Simonne Griffith-Jones, Lucía Álvarez, Urbi Mukhopadhyay, Sarah Gharbi, Mandy Rettel, Michael Adams, Janosch Hennig, Sagar Bhogaraju*

Table of Contents

	Page
**Appendix Figure S1**	2
**Appendix Figure S2**	3
**Appendix Table S1**	4
**Appendix Table S2**	5

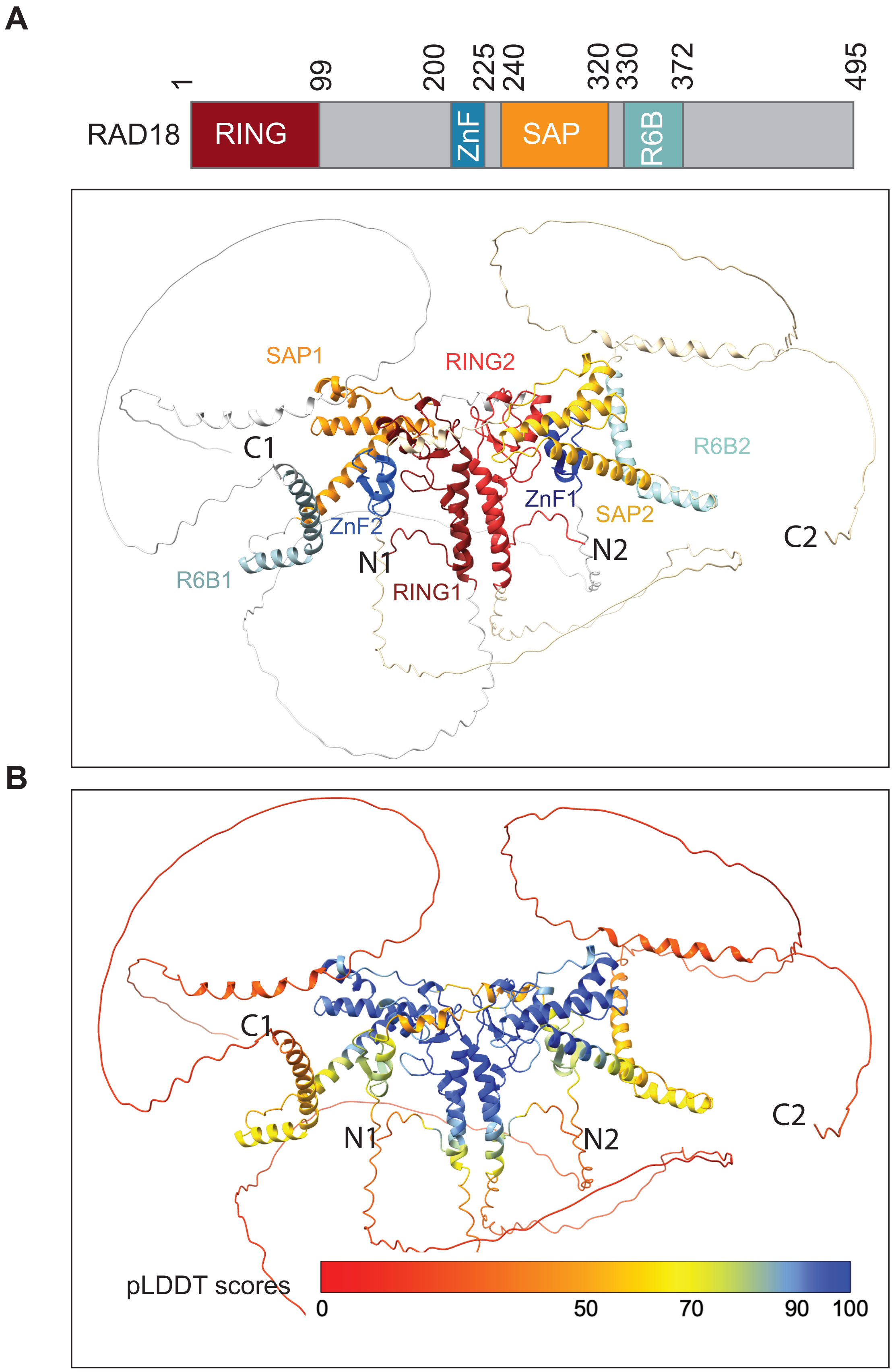

Appendix Figure S1: AlphaFold2 model of full-length dimeric RAD18 (Uniprot Q9NS91 residues 1-495), performed using AlphaFold multimer.

A. Domain boundaries of RAD18 redefined according to the AF model (top). The AF model suggests that the R6BD of RAD18 is likely to occur between residues 330-372, rather than the previously defined residues 340-395 (Notenboom et al, 2007). Similarly, the SAP domain is likely to reside at residues 240-320, instead of residues 248-282, as previously reported (Notenboom et al, 2007). RAD18 is coloured according to the new domain boundaries.

B. AlpaFold2 model of dimeric RAD18 coloured by pLDDT score.

```
Colored PROMALS3D alignment (sequences in aligned order)
Conservation:                                   5  7   9  666 75  5   575677  7        9  75  67        77  7 7
MAGEC1_MHD_AF-O60732.pdb_     1  ---------LDEKVDELARFLLLKYQVKQPITKAEMLTNVISRYTGYFPVIFRKAREFIEILFGISLREV   61
MAGEC2_MHD_AF-Q9UBF1.pdb_     1  ---------LDEKVAELVEFLLLKYEAEEPVTEAEMLMIVI-KYKDYFPVILKRAREFMELLFGLALIEV   60
MAGEB18_MHD_AF-Q96M61.pdb_    1  ---------LNKKVVSLVHFLLQKYETKEPITKGDMIKFVIRKDKCHFNEILKRASEHMELALGVDLKEV   61
MAGEB10_MHD_AF-Q96LZ2.pdb_    1  ---------VDEKVIILVHYLLYKYQMKEPITKADMLRNVTQMSKSQFPVILSRASEHLELIFGLDLKEV   61
MAGEB16_MHD_AF-A0A0A0MSA3     1  ---------LDQKVAFLVNFMLHKCQMKKPITKADMLKIIIKDDESHFSEILLRASEHLEMIFGLDVVEV   61
MAGEB3_MHD_AF-O15480.pdb_     1  ---------LIMKTNMLVQFLMEMYKMKKPIMKADMLKIVQKSHKNCFPEILKKASFNMEVVFGVDLKKV   61
MAGEB4_MHD_AF-O15481.pdb_     1  ---------LTRKTKMLVQFLLYKYKMKEPTTKAEMLKIISKKYKEHFPEIFRKVSQRTELVFGLALKEV   61
MAGEB2_MHD_AF-O15479.pdb_     1  ---------LTRKSGSLVQFLLYKYKIKKSVTKGEMLKIVGKRFREHFPEILKKASEGLSVVFGLELNKV   61
MAGEB1_MHD_AF-P43366.pdb_     1  ---------VAWEAGMLMHFILRKYKMREPIMKADMLKVVDEKYKDHFTEILNGASRRLELVFGLDLKED   61
MAGEB5_MHD_AF-Q9BZ81.pdb_     1  ---------INIKVGLLEQFLLYKFKMKQRILKEDMLKIVNPRYQNQFAEIHRRASEHIEVVFAVDLKEV   61
MAGEB6_MHD_AF-Q8N7X4.pdb_     1  ---------VKKKACTLAQFLQKKFEKKESILKADMLKCVRREYKPYFPQILNRTSQHLVVAFGVELKEM   61
MAGEA12_MHD_AF-P43365.pdb     1  ---------LSRKMAELVHFLLLKYRAREPFTKAEMLGSVIRNFQDFFPVIFSKASEYLQLVFGIEVVEV   61
MAGEA2_MHD_AF-P43356.pdb_     1  ---------ISRKMVELVHFLLLKYRAREPVTKAEMLESVLRNCQDFFPVIFSKASEYLQLVFGIEVVEV   61
MAGEA6_MHD_AF-P43360.pdb_     1  ---------LSRKVAKLVHFLLLKYRAREPVTKAEMLGSVVGNWQYFFPVIFSKASDSLQLVFGIELMEV   61
MAGEA3_MHD_AF-P43357.pdb_     1  ---------LSRKVAELVHFLLLKYRAREPVTKAEMLGSVVGNWQYFFPVIFSKASSSLQLVFGIELMEV   61
MAGEA4_MHD_AF-Q1RN33.pdb_     1  DAESLFREALSNKVDELAHFLLRKYRAKELVTKAEMLERVIKNYKRCFPVIFGKASESLKMIFGIDVKEV   70
MAGEA1_MHD_AF-P43355.pdb_     1  ---------ITKKVADLVGFLLLKYRAREPVTKAEMLESVIKNYKHCFPEIFGKASESLQLVFGIDVKEA   61
MAGEA11_MHD_AF-P43364.pdb     1  ---------LHDKIIDLVHLLLRKYRVKGLITKAEMLGSVIKNYEDYFPEIFREASVCMQLLFGIDVKEV   61
MAGEA10_MHD_AF-P43363.pdb     1  ---------IDEKVTDLVQFLLFKYQMKEPITKAEILESVIRNYEDHFPLLFSEASECMLLVFGIDVKEV   61
MAGEA9_MHD_AF-P43362.pdb_     1  ---------LKLKVAELVHFLLHKYRVKEPVTKAEMLESVIKNYKRYFPVIFGKASEFMQVIFGTDVKEV   61
Consensus_aa:                    .........lp.Khs.LhpFLL.KYph+cslhKA-ML..V.pp@p.hFs.Ihp+ASp.hpllFGl-l.EV
Consensus_ss:                                hhhhhhhhhhhhhhh      hhhhhhhhhhhhhhhhhhhhhhhhhhhhhh eeeeee

Conservation:                                6 7    5 9  5           7   695   9  75 57     79  95 5        7  5  5
MAGEC1_MHD_AF-O60732.pdb_    62  -DPDDSYVFVNTLDLTSEGCLSDEQGMSQNRLLILILSIIFIKGTYASEEVIWDVLSGIGVRAGREHFAF  130
MAGEC2_MHD_AF-Q9UBF1.pdb_    61  -GPDHFCVFANTVGLTDEG--SDDEGMPENSLLIIILSVIFIKGNCASEEVIWEVLNAVGVYAGREHFVY  127
MAGEB18_MHD_AF-Q96M61.pdb_   62  DPIRHYYAFFSKLDLTYDETTSDEEKIPKTGLLMIALGVIFLNGNRAPEEAVWEIMNMMGVYADRKHFLY  131
MAGEB10_MHD_AF-Q96LZ2.pdb_   62  EPNKHIYVLVNKLDLGCDAKLSDETGVPKTGLLMTVLGIIFTNGNCVAEEEVWKVFNTMGLYDGIEHFMF  131
MAGEB16_MHD_AF-A0A0A0MSA3    62  DPTTHCYGLFIKLGLTYDGMLSGEKGVPKTGLLIIVLGVIFMKGNRATEEEVWEVLNLTGVYSGKKHFIF  131
MAGEB3_MHD_AF-O15480.pdb_    62  DSTKDSYVLVSKMDLPNNGTVTRGRGFPKTGLLLNLLGVIFMKGNCATEEKIWEFLNKMRIYDGKKHFIF  131
MAGEB4_MHD_AF-O15481.pdb_    62  NPTTHSYILVSMLG-PNDGNQSSAWTLPRNGLLMPLLSVIFLNGNCAREEEIWEFLNMLGIYDGKRHLIF  130
MAGEB2_MHD_AF-O15479.pdb_    62  NPNGHTYTFIDKVDLTDEESLLSSWDFPRRKLLMPLLGVIFLNGNSATEEEIWEFLNMLGVYDGEEHSVF  131
MAGEB1_MHD_AF-P43366.pdb_    62  NPSGHTYTLVSKLNLTNDGNLSNDWDFPRNGLLMPLLGVIFLKGNSATEEEIWKFMNVLGAYDGEEHLIY  131
MAGEB5_MHD_AF-Q9BZ81.pdb_    62  NPTCHLYDLVSKLKLPNNGRIHVGKVLPKTGLLMTFLVVIFLKGNCANKEDTWKFLDMMQIYDGKKYYIY  131
MAGEB6_MHD_AF-Q8N7X4.pdb_    62  DSSGESYTLVSKLGLPSEGILSGDNALPKSGLLMSLLVVIFMNGNCATEEEVWEFLGLLGIYDGILHSIY  131
MAGEA12_MHD_AF-P43365.pdb    62  VRIGHLYIILVTCLGLSYDGLLGDNQIVPKTGLLIIVLAIIAKEGDCAPEEKIWEELSVLEASDGREDSVF  131
MAGEA2_MHD_AF-P43356.pdb_    62  VPISHLYILVTCLGLSYDGLLGDNQVMPKTGLLIIVLAIIAIEGDCAPEEKIWEELSMLEVFEGREDSVF  131
MAGEA6_MHD_AF-P43360.pdb_    62  DPIGHVYIFATCLGLSYDGLLGDNQIMPKTGFLIIILAIIAKEGDCAPEEKIWEELSVLEVFEGREDSIF  131
MAGEA3_MHD_AF-P43357.pdb_    62  DPIGHLYIFATCLGLSYDGLLGDNQIMPKAGLLIIVLAIIAREGDCAPEEKIWEELSVLEVFEGREDSIL  131
MAGEA4_MHD_AF-Q1RN33.pdb_    71  DPTSNTYTLVTCLGLSYDGLLGNNQIFPKTGLLIIVLGTIAMEGDSASEEEIWEELGVMGVYDGREHTVY  140
MAGEA1_MHD_AF-P43355.pdb_    62  DPTGHSYVLVTCLGLSYDGLLGDNQIMPKTGFLIIVLVMIAMEGGHAPEEEIWEELSVMEVYDGREHSAY  131
MAGEA11_MHD_AF-P43364.pdb    62  DPTSHSYVLVTSLNLSYDGIQCNEQSMPKSGLLIIVLGVIFMEGNCIPEEVMWEVLSIMGVYAGREHFLF  131
MAGEA10_MHD_AF-P43363.pdb    62  DPTGHSFVLVTSLGLTYDGMLSDVQSMPKTGILILILSIVFIEGYCTPEEVIWEALNMMGLYDGMEHLIY  131
MAGEA9_MHD_AF-P43362.pdb_    62  DPAGHSYILVTALGLSCDSMLGDGHSMPKAALLIIVLGVILTKDNCAPEEVIWEALSVMGVYVGKEHMFY  131
Consensus_aa:                    ssssc.Yhhhs.LsLs.-t.lts.p.hP+stLLh.lLtlIhhpGs.AsEE.lW-.Lshh.l@sGccc.h@
Consensus_ss:                         eeeeee                         hhhhhhhhhhh        hhhhhhhhhh

Conservation:                    7575   575 595   9759 796 7 96 779999779  9  7 676           5       6   5
MAGEC1_MHD_AF-O60732.pdb_   131  GEPRELLTKVWVQEHYLEYREVPNSSPPRYEFLWGPRAHSEVIKRKVVEFLAMLKNTVPITFPSSYKDA-   199
MAGEC2_MHD_AF-Q9UBF1.pdb_   128  GEPRELLTKVWVQGHYLEYREVPHSSPPYYEFLWGPRAHSESIKKKVLEFLAKLNNTVPSSFPSWYKDA-   196
MAGEB18_MHD_AF-Q96M61.pdb_  132  GDPRKVMTKDLVQLKYLEYQQVPNSDPPRYEFLWGPRAHAETSKMKVLEFVAKIHDTVPSA----FPSCY   197
MAGEB10_MHD_AF-Q96LZ2.pdb_  132  GEPRKLLTKDLVKENYLEYQQVPNSDPPRYQFLWGPRAHAETSKMKVLEFLAKVNDTAPSE----FSNWY   197
MAGEB16_MHD_AF-A0A0A0MSA3   132  GEPRMLITKDFVKEKYLEYQQVANSDPARYEFLWGPRAKAETSKMKVLEFVAKVHGSYPHS----FPSQY   197
MAGEB3_MHD_AF-O15480.pdb_   132  GEPRKLITQDLVKLKYLEYRQVPNSNPARYEFLWGPRAHAETSKMKVLEFWAKVNKTVPSA----FQFWY   197
MAGEB4_MHD_AF-O15481.pdb_   131  GEPRKLITQDLVQEKYLEYQQVPNSDPPRYQFLWGPRAHAETSKMKVLEFLAKVNDTTPNN----FPLLY   196
MAGEB2_MHD_AF-O15479.pdb_   132  GEPWKLITKDLVQEKYLEYKQVPSSDPPRFQFLWGPRAYAETSKMKVLEFLAKVNGTTPCA----FPTHY   197
MAGEB1_MHD_AF-P43366.pdb_   132  GEPRKFITQDLVQEKYLKYEQVPNSDPPRYQFLWGPRAYAETTKMKVLEFLAKMNGATPRD----FPSHY   197
MAGEB5_MHD_AF-Q9BZ81.pdb_   132  GEPRKLITQDFVRLTYLEYHQVPCSYPAHYQFLWGPRAYTETSKMKVLEYLAKVNDIAPGA----FSSQY   197
MAGEB6_MHD_AF-Q8N7X4.pdb_   132  GDARKIITEDLVQDKYVVYRQVCNSDPPCYEFLWGPRAYAETTKMRVLRVLADSSNTSPGL----YPHLY   197
MAGEA12_MHD_AF-P43365.pdb   132  AHPRKLLTQDLVQENYLEYRQVPGSDPACYEFLWGPRALVETSYVKVLHHLLKISGGPHIS----YPPLH   197
MAGEA2_MHD_AF-P43356.pdb_   132  AHPRKLLMQDLVQENYLEYRQVPGSDPACYEFLWGPRALIETSYVKVLHHTLKIGGEPHIS----YPPLH   197
MAGEA6_MHD_AF-P43360.pdb_   132  GDPKKLLTQYFVQENYLEYRQVPGSDPACYEFLWGPRALIETSYVKVLHHMVKISGGPRIS----YPLLH   197
MAGEA3_MHD_AF-P43357.pdb_   132  GDPKKLLTQHFVQENYLEYRQVPGSDPACYEFLWGPRALVETSYVKVLHHMVKISGGPHIS----YPPLH   197
MAGEA4_MHD_AF-Q1RN33.pdb_   141  GEPRKLLTQDWVQENYLEYRQVPGSNPARYEFLWGPRALAETSYVKVLEHVVRVNARVRIA----YPSLR   206
MAGEA1_MHD_AF-P43355.pdb_   132  GEPRKLLTQDLVQEKYLEYRQVPDSDPARYEFLWGPRALAETSYVKVLEYVIKVSARVRFF----FPSLR   197
MAGEA11_MHD_AF-P43364.pdb   132  GEPRKLLTQNWVQEKYLVYRQVPGTDPACYEFLWGPRAHAETSKMKVLEYIANANGRDPTS----YPSLY   197
MAGEA10_MHD_AF-P43363.pdb   132  GEPRKLLTQDWVQENYLEYRQVPGSDPARYEFLWGPRAHAEIRKMSLLKFLAKVNGSDPRS----FPLWY   197
MAGEA9_MHD_AF-P43362.pdb_   132  GEPRKLLTQDWVQENYLEYRQVPGSDPAHYEFLWGSKAHAETSYEKVINYLVMLNAREPIC----YPSLY   197
Consensus_aa:                    G-PR+LlTpshVpbpYLEYcQVPsSsPs.YpFLWGPRAhhETobhKVLc@lhclss.s..s....@s.h@
Consensus_ss:                      hhhhhhhhhhh    eeeee        eeeeee          hhhhhhhhhhh         hhh

Conservation:                    6 7
MAGEC1_MHD_AF-O60732.pdb_        -----------
MAGEC2_MHD_AF-Q9UBF1.pdb_        -----------
MAGEB18_MHD_AF-Q96M61.pdb   198  EEA--------  200
MAGEB10_MHD_AF-Q96LZ2.pdb   198  TEA--------  200
MAGEB16_MHD_AF-A0A0A0MSA3   198  AEA--------  200
MAGEB3_MHD_AF-O15480.pdb_   198  EEA--------  200
MAGEB4_MHD_AF-O15481.pdb_   197  EEA--------  199
MAGEB2_MHD_AF-O15479.pdb_   198  EEA--------  200
MAGEB1_MHD_AF-P43366.pdb_   198  EEA--------  200
MAGEB5_MHD_AF-Q9BZ81.pdb_   198  EEA--------  200
MAGEB6_MHD_AF-Q8N7X4.pdb_   198  EDA--------  200
MAGEA12_MHD_AF-P43365.pdb   198  EWA--------  200
MAGEA2_MHD_AF-P43356.pdb_   198  ERA--------  200
MAGEA6_MHD_AF-P43360.pdb_   198  EWA--------  200
MAGEA3_MHD_AF-P43357.pdb_   198  EWV--------  200
MAGEA4_MHD_AF-Q1RN33.pdb_   207  EAALLEEEEGV  217
MAGEA1_MHD_AF-P43355.pdb_   198  EAA--------  200
MAGEA11_MHD_AF-P43364.pdb   198  EDA--------  200
MAGEA10_MHD_AF-P43363.pdb   198  EEA--------  200
MAGEA9_MHD_AF-P43362.pdb_   198  EEV--------  200
Consensus_aa:                    E.A........
Consensus_ss:                    hh
```

MAGEA11 Substrate-binding cleft

MAGEA4 Ligase-binding cleft

Appendix Figure S2: Structure based multiple sequence alignment of MHD domains from various type 1 MAGEs was performed using the PROMALS3D server. Residues lining the substrate-binding cleft and the ligase-binding cleft are highlighted in green and pink respectively.

Appendix Table S1: Ubiquitination sites of RAD18 and ubiquitin chain linkages detected in the autoubiquitinated RAD18/RAD6 sample

	Modified residue	Number of reads	diGly Score
RAD18 Residue	K83	2	45,4
	K102	4	77,8
	K110	5	98,9
	K115	4	81,1
	K151	6	68,5
	K154	20	69,1
	K156	22	77,4
	K161	9	63,6
	K168	8	68,5
	K170	3	60,7
	K186	5	64,1
	K197	4	66,9
	K241	0	N/A
	K245	3	51,0
	K257	2	50,7
	K258	1	52,4
	K259	1	71,0
	K261	8	60,3
	K271	11	67,8
	K276	6	72,2
	K309	4	73,1
	K318	24	63,3
	K328	17	77,2
	K333	5	87,8
	K341	6	88,5
	K345	2	76,5
	K347	4	65,5
	K363	2	46,0
	K370	14	66,0
	K376	6	74,8
	K383	2	80,6
	K462	9	150,4
Ubiquitin Residue	K63	24	83,1
	K48	15	56,9
	K11	16	66,5
	K6	12	35,8

Appendix Table S2: Oligonucleotides used in this study

	Construct	Forward primer (5'-3')	Reverse primer (5'-3')
**Primers**	**RAD18_F350D**	GCCTGATCCACCAGAAGCTGATCTTCACTCTTATGTTTTTTACGATATT	AATATCGTAAAAAACATAAGAGTGAAGATCAGCTTCTGGTGGATCAGGC
	RAD18_V354D	GTATCCTTTTCTAGCCTGATCATCCAGAAGCTGAAATTCACTCT	AGAGTGAATTTCAGCTTCTGGATGATCAGGCTAGAAAAGGATAC
	RAD18_A357D	TTTTCTTGTATCCTTTTCTATCCTGATCCACCAGAAGCTG	CAGCTTCTGGTGGATCAGGATAGAAAAGGATACAAGAAAA
	RAD18_Y361D	TTCCAGCAATTTTCTTGTCTCCTTTTCTAGCCTGATCCAC	GTGGATCAGGCTAGAAAAGGAGACAAGAAAATTGCTGGAA
	RAD18_R51A	CACATAACTACTGCTCTCTCTGTATAGCAAAATTTCTGTCCTATAAAACTCAGTG	CACTGAGTTTTATAGGACAGAAATTTTGCTATACAGAGAGAGCAGTAGTTATGTG
	RAD18_R51E	TTCACATAACTACTGCTCTCTCTGTATAGAGAAATTTCTGTCCTATAAAACTCAGTGTC	GACACTGAGTTTTATAGGACAGAAATTTCTCTATACAGAGAGAGCAGTAGTTATGTGAA
	RAD18_A287D	GTACACATGTACAATGACCAATGCGATGCTTTGC	GCAAAGCATCGCATTGGTCATTGTACATGTGTAC
	MAGEA4_M161D	CTTCACGTCAATGCCAAAGATATCCTTCAGGGACTCGGAGGCTTT	AAAGCCTCCGAGTCCCTGAAGGATATCTTTGGCATTGACGTGAAG
	MAGEA4_I205D	TGCCCAGGACGTCTATCAGAAGGCCTGTCTTGGG	CCCAAGACAGGCCTTCTGATAGACGTCCTGGGCA
	MAGEA4_L288D	ATTGACCCTGACCACATGCTCATCGACTTTCACATAGCTGGTTTC	GAAACCAGCTATGTGAAAGTCGATGAGCATGTGGTCAGGGTCAAT
	MAGEA4_L121A	GATGAGTTGGCTCATTTTGCGCTCCGCAAGTATCGAG	CTCGATACTTGCGGAGCGCAAAATGAGCCAACTCATC
	MAGEA4_L122A	GTTGGCTCATTTTGCGGCCCGCAAGTATCGAGC	GCTCGATACTTGCGGGCCGCAAAATGAGCCAAC
	MAGEC2_L318D	GCATCAAGAAGAAAGTACTAGAGTTTGATGCCAAGCTGAACAACACTG	CAGTGTTGTTCAGCTTGGCATCAAACTCTAGTACTTTCTTCTTGATGC
	pCoofy1-RAD6-His-removal	GTTTAACTTTAAGAAGGAGATATACCATGTCGACCCCGGCCC	GGGCCGGGGTCGACATGGTATATCTCCTTCTTAAAGTTAAAC
**Oligonucleotide used in ubiquitination assay in the presence of DNA**			5'-GAAAACGCGGCGCAAGACCTGGACAAGATCGAG-3'
