# Supplementary material for: Structural basis for RAD18 regulation by MAGEA4 and its implications for RING ubiquitin ligase binding by MAGE family proteins
Source: EMBO J. 2024 Mar 6;43(7):1273–300. doi: 10.1038/s44318-024-00058-9 (PMC10987633; doi:10.1038/s44318-024-00058-9)
Supplement: Supplementary file 4 — Source Data Fig. 1 [file 44318_2024_58_MOESM4_ESM.zip › Figure 1/1F/README.rtf]

The Microcal PEAQ-ITC Analysis Software (Malvern) was used to calculate KD, ∆, -T∆ (kcal/mol) and the exported values are presented in the first Tab. The raw values are given in the subsequent tabs. Three replicates were conducted for MAGEA4 WT, the first replicate was chosen for figure 1F.
