# Supplementary figures and images for: Structural basis for RAD18 regulation by MAGEA4 and its implications for RING ubiquitin ligase binding by MAGE family proteins
Source: EMBO J. 2024 Mar 6;43(7):1273–300. doi: 10.1038/s44318-024-00058-9 (PMC10987633; doi:10.1038/s44318-024-00058-9)

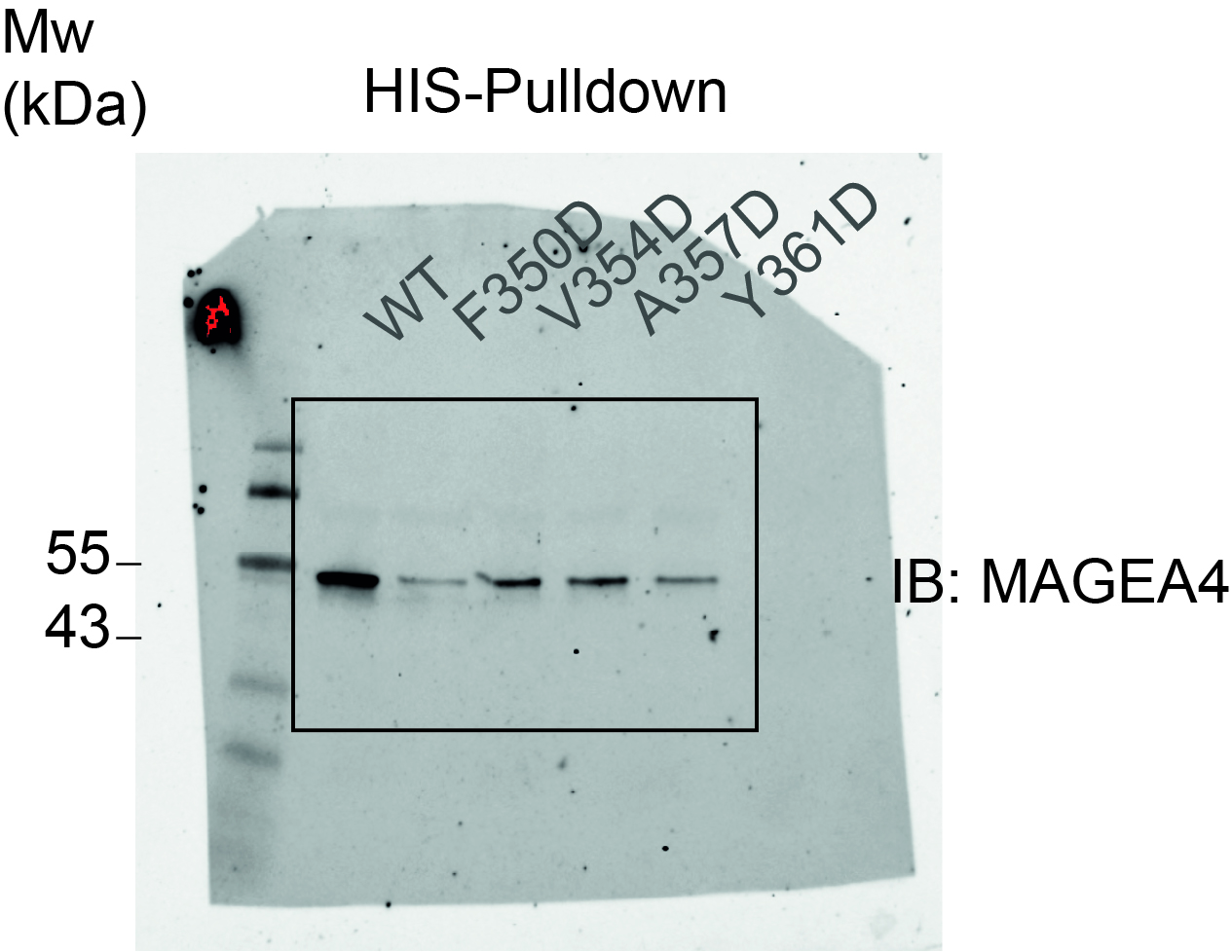

Supplement: Supplementary file 4 — Source Data Fig. 1 [file 44318_2024_58_MOESM4_ESM.zip › Figure 1/1E/Pulldown_MAGEA4Model.jpg]

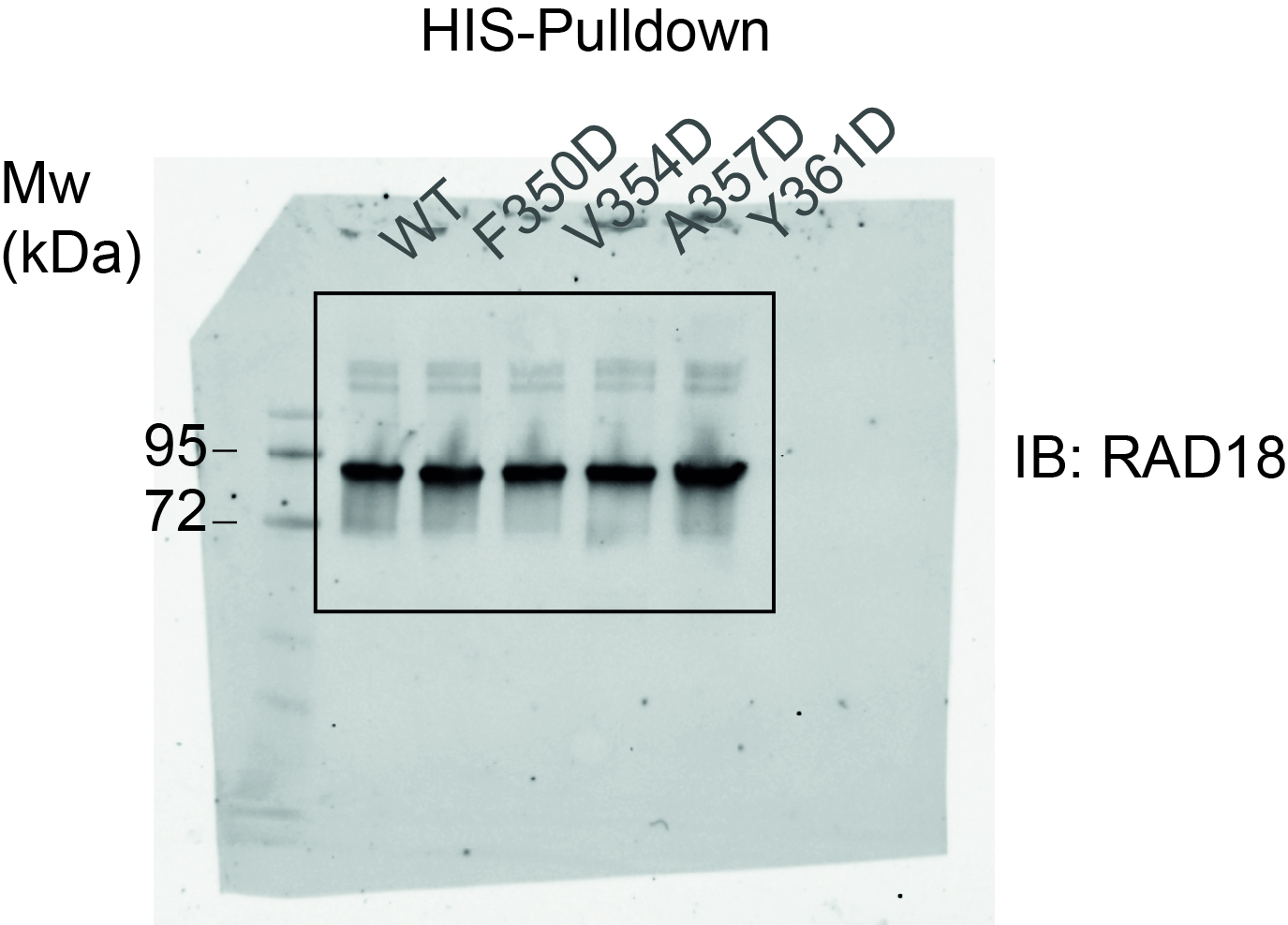

Supplement: Supplementary file 4 — Source Data Fig. 1 [file 44318_2024_58_MOESM4_ESM.zip › Figure 1/1E/Pulldown_RAD18Model.jpg]

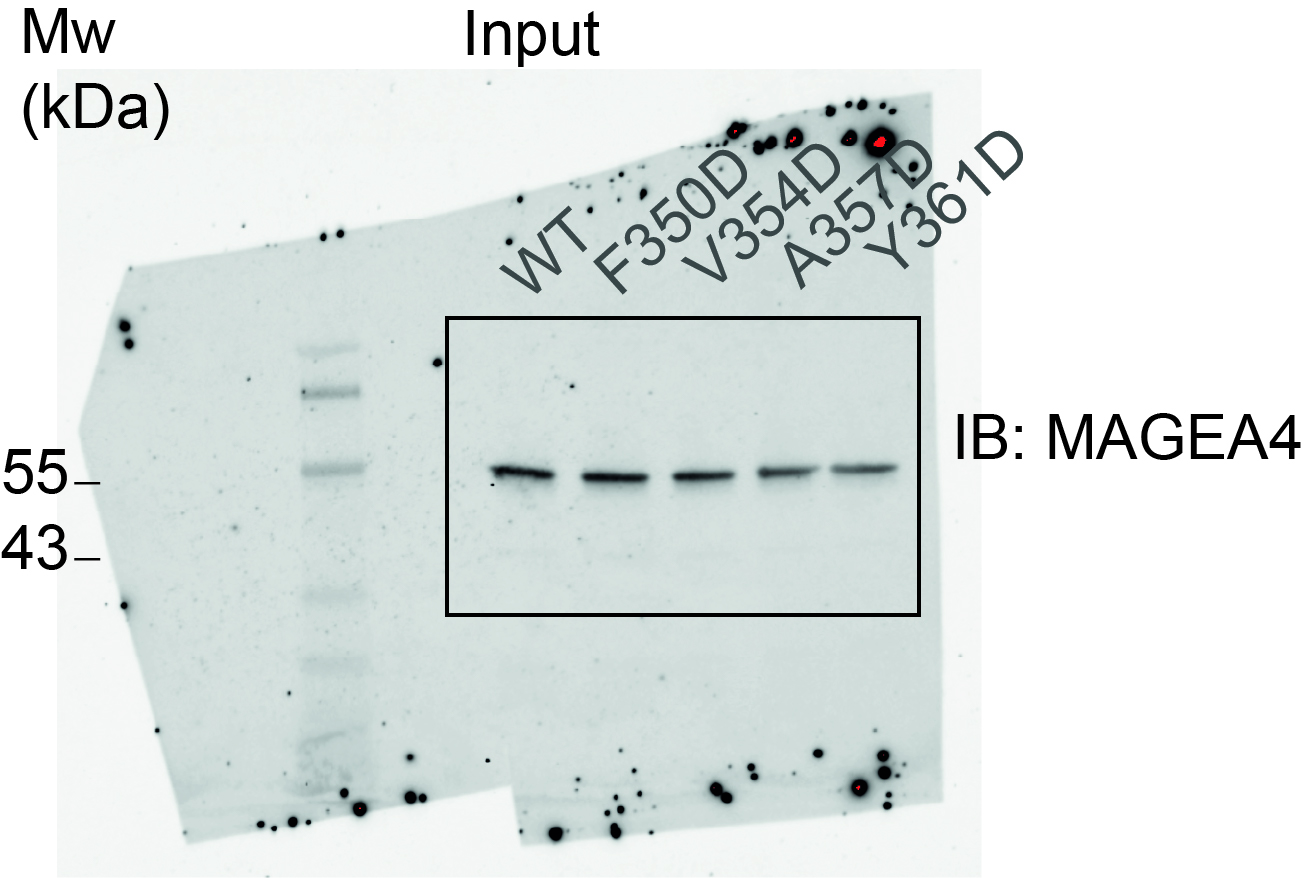

Supplement: Supplementary file 4 — Source Data Fig. 1 [file 44318_2024_58_MOESM4_ESM.zip › Figure 1/1E/Input_MAGEA4Model.jpg]

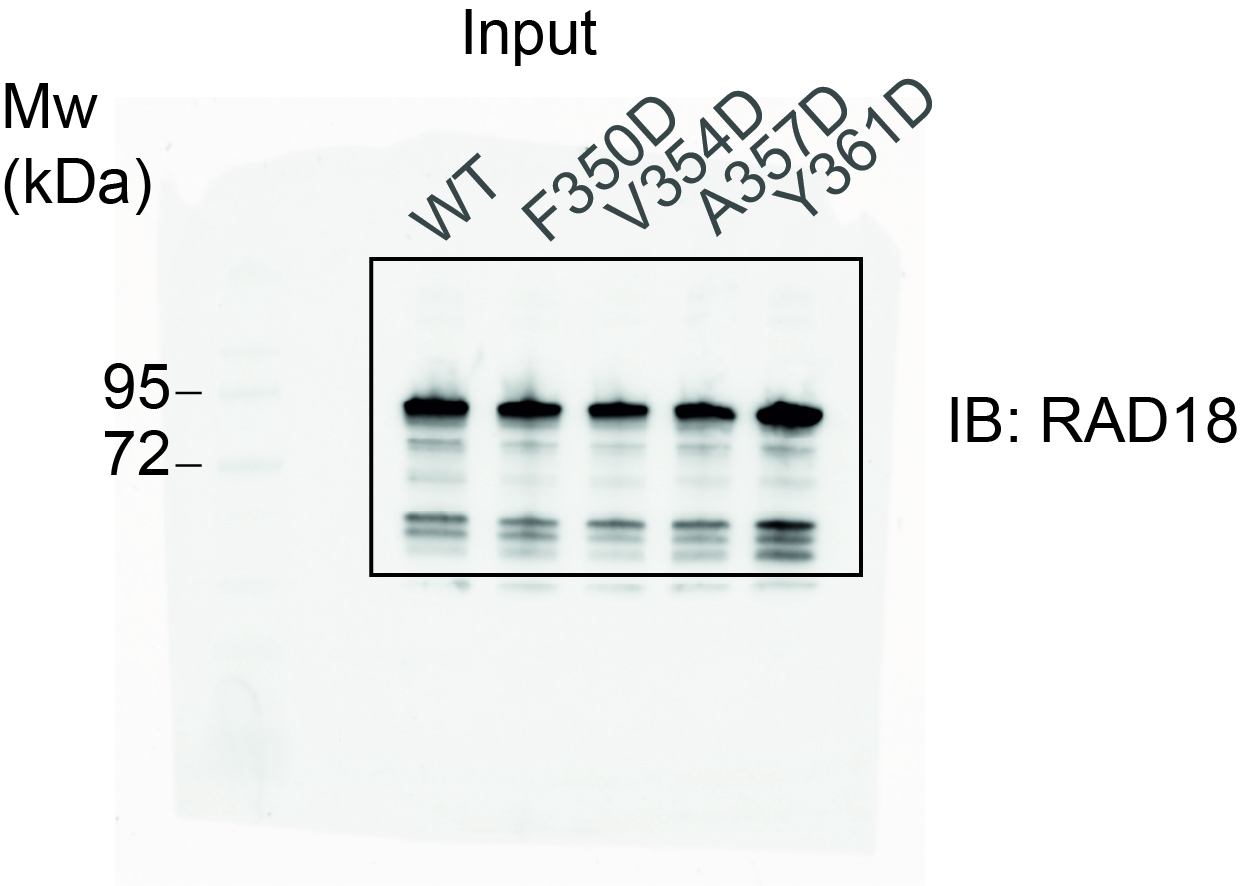

Supplement: Supplementary file 4 — Source Data Fig. 1 [file 44318_2024_58_MOESM4_ESM.zip › Figure 1/1E/Input_RAD18Model.jpg]

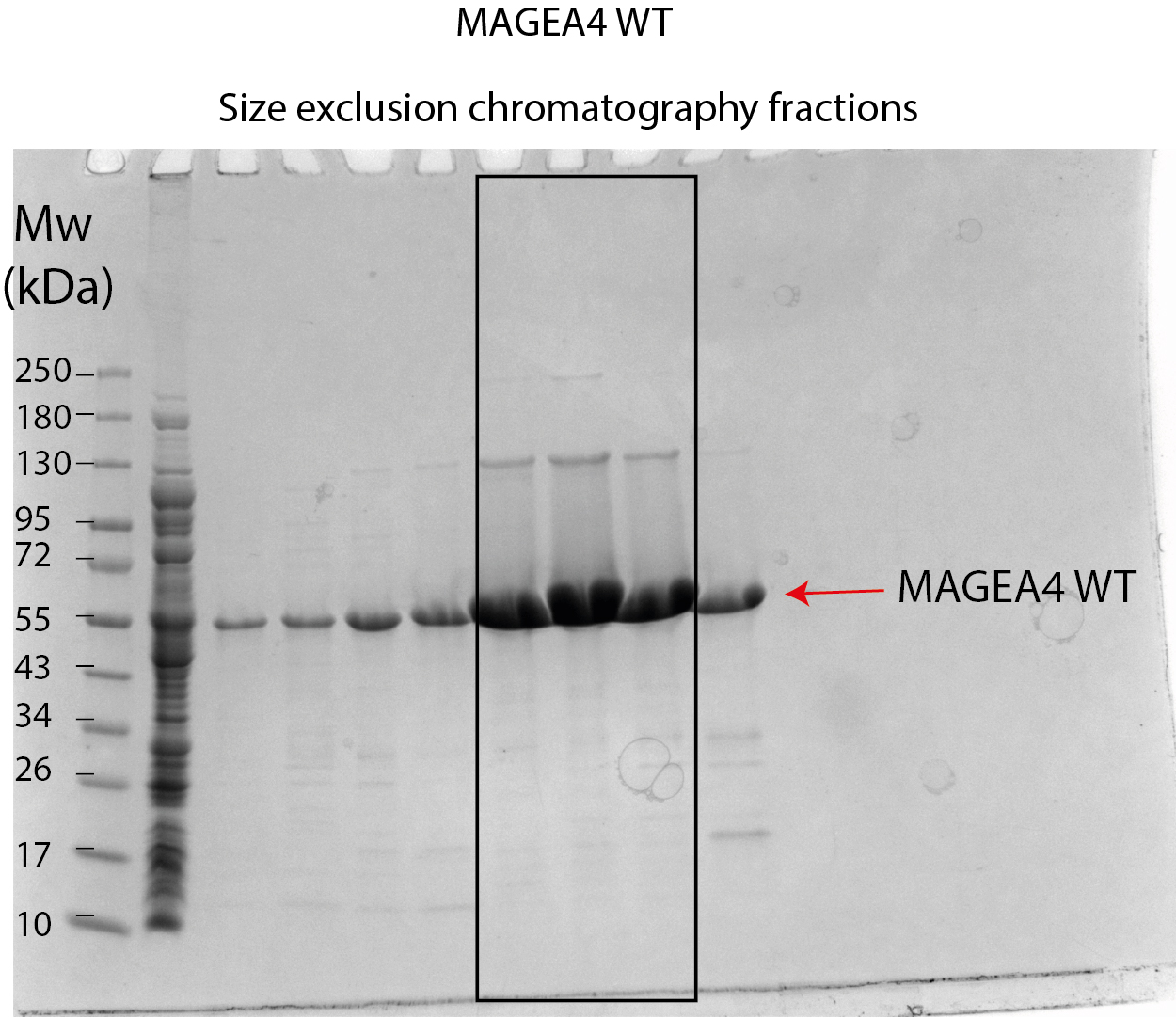

Supplement: Supplementary file 5 — Source Data Fig. 2 [file 44318_2024_58_MOESM5_ESM.zip › Figure 2/2C/MAGEA4_WT_SEC.jpg]

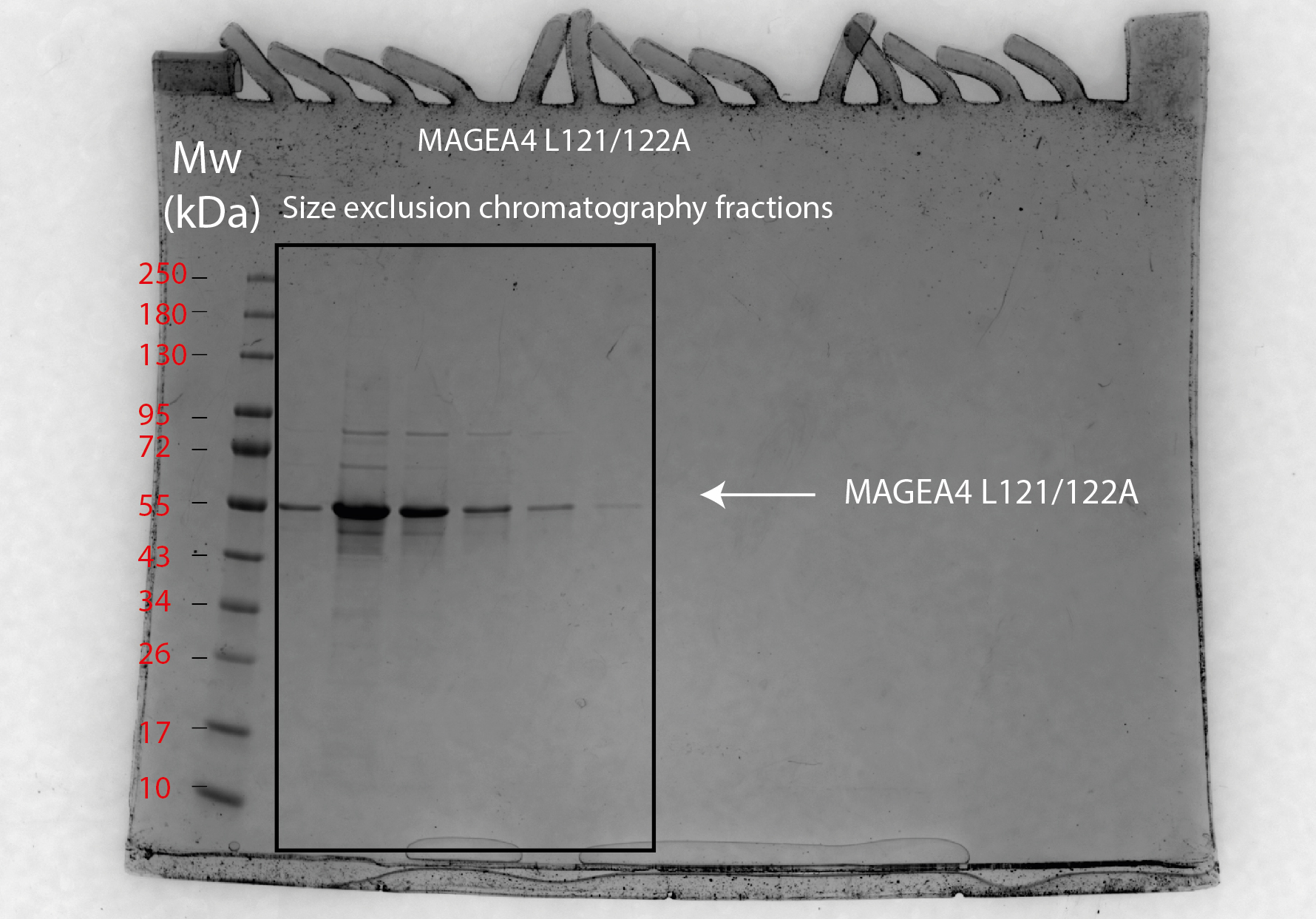

Supplement: Supplementary file 5 — Source Data Fig. 2 [file 44318_2024_58_MOESM5_ESM.zip › Figure 2/2C/MAGEA4_Di-L_SEC.jpg]

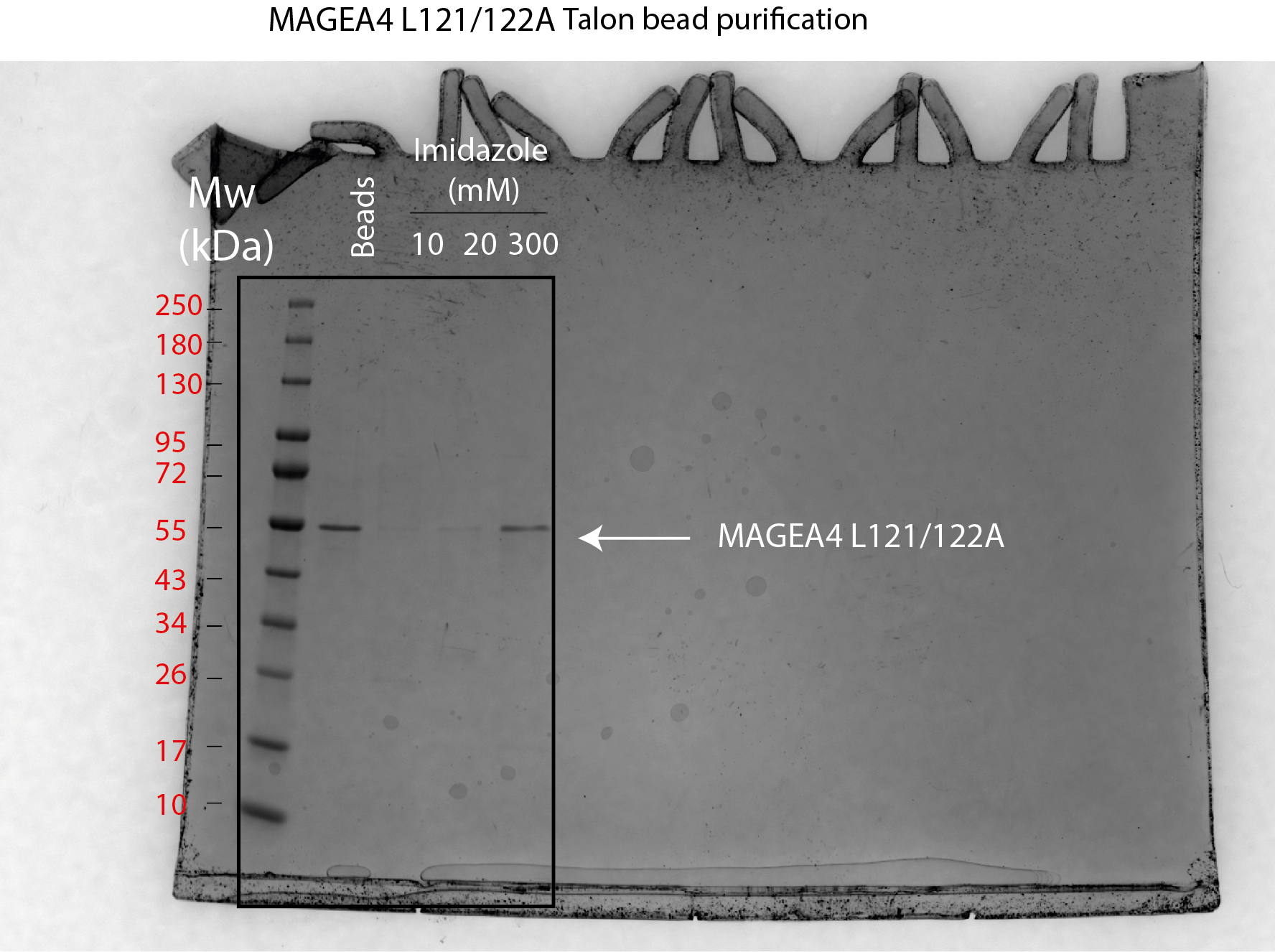

Supplement: Supplementary file 5 — Source Data Fig. 2 [file 44318_2024_58_MOESM5_ESM.zip › Figure 2/2B/MAGEA4_Di-L_TALON.jpg]

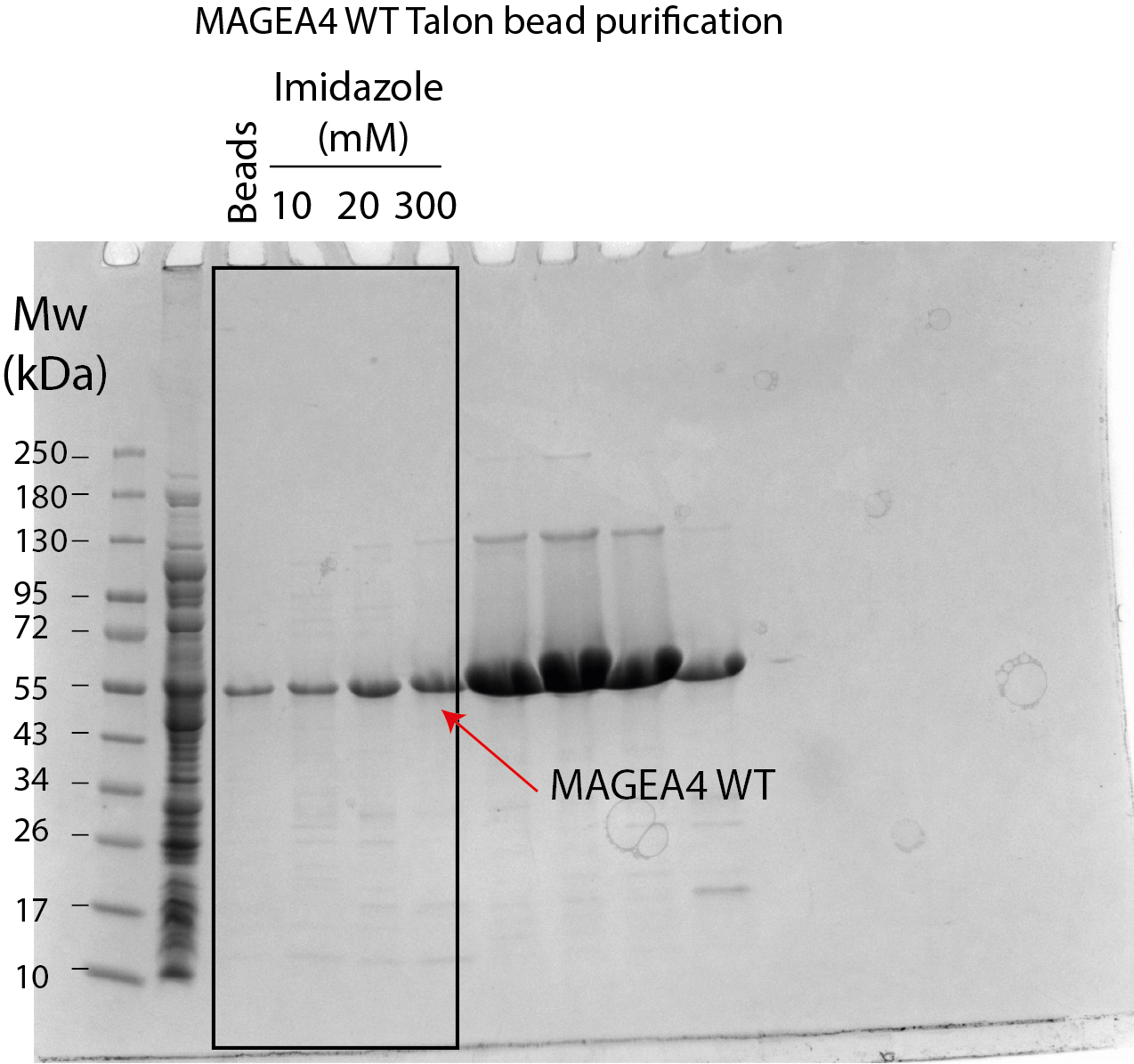

Supplement: Supplementary file 5 — Source Data Fig. 2 [file 44318_2024_58_MOESM5_ESM.zip › Figure 2/2B/MAGEA4_WT_Talon.jpg]

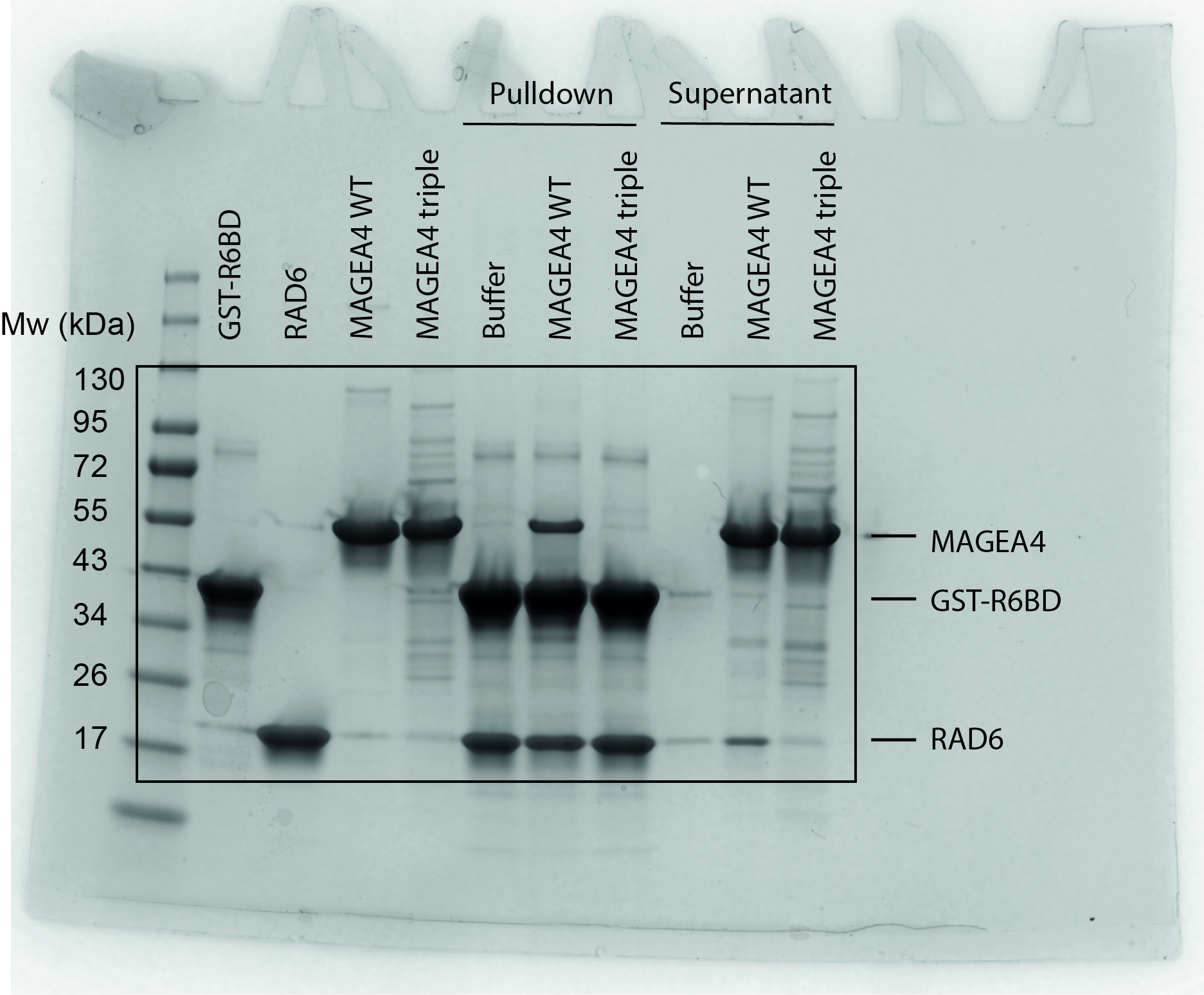

Supplement: Supplementary file 6 — Source Data Fig. 3 [file 44318_2024_58_MOESM6_ESM.zip › Figure 3/3C/Asset 1Model.jpg]

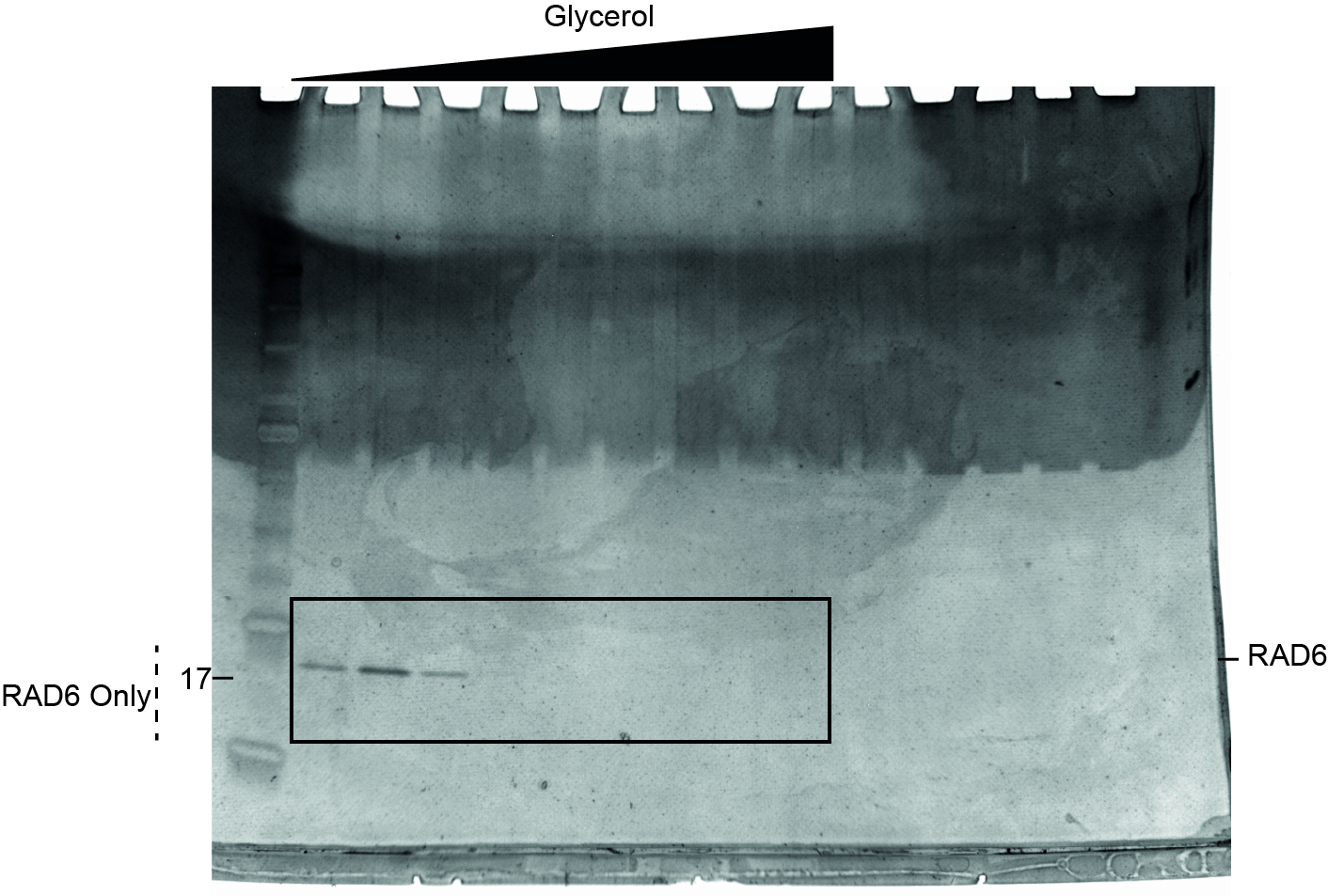

Supplement: Supplementary file 6 — Source Data Fig. 3 [file 44318_2024_58_MOESM6_ESM.zip › Figure 3/3D/RAD6Model.jpg]

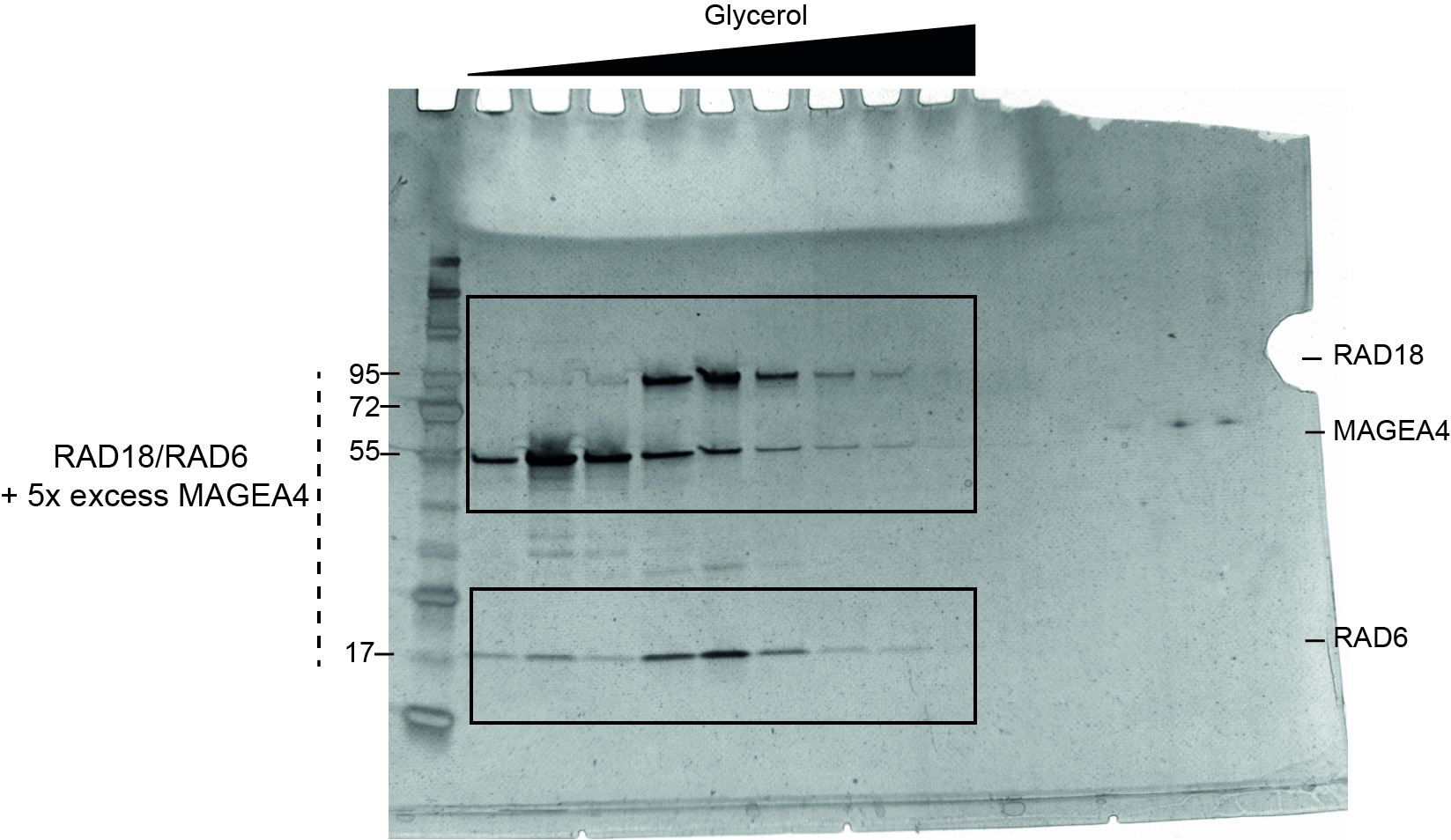

Supplement: Supplementary file 6 — Source Data Fig. 3 [file 44318_2024_58_MOESM6_ESM.zip › Figure 3/3D/RAD18RAD6MAGEA4Model.jpg]

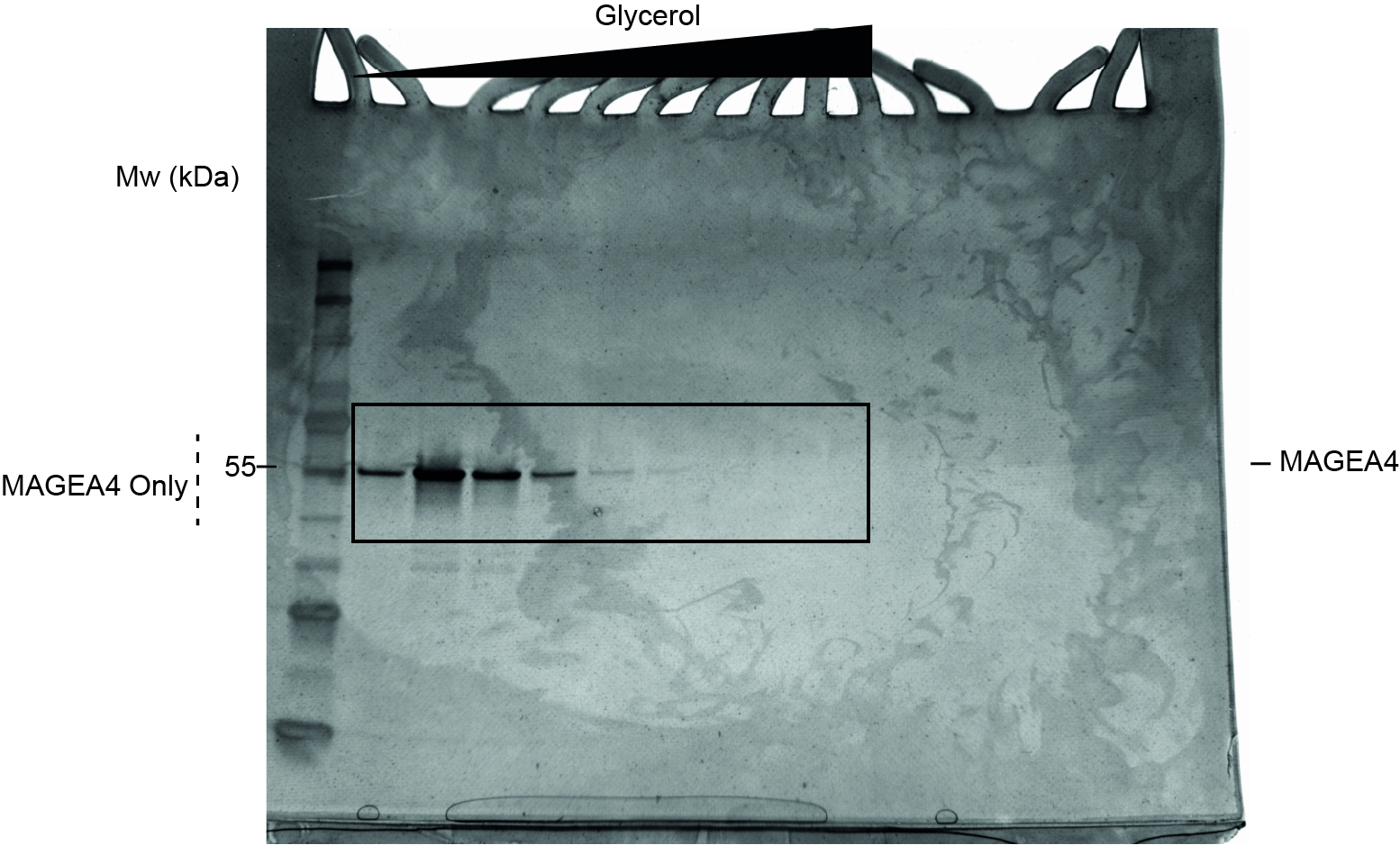

Supplement: Supplementary file 6 — Source Data Fig. 3 [file 44318_2024_58_MOESM6_ESM.zip › Figure 3/3D/MAGEA4Model.jpg]

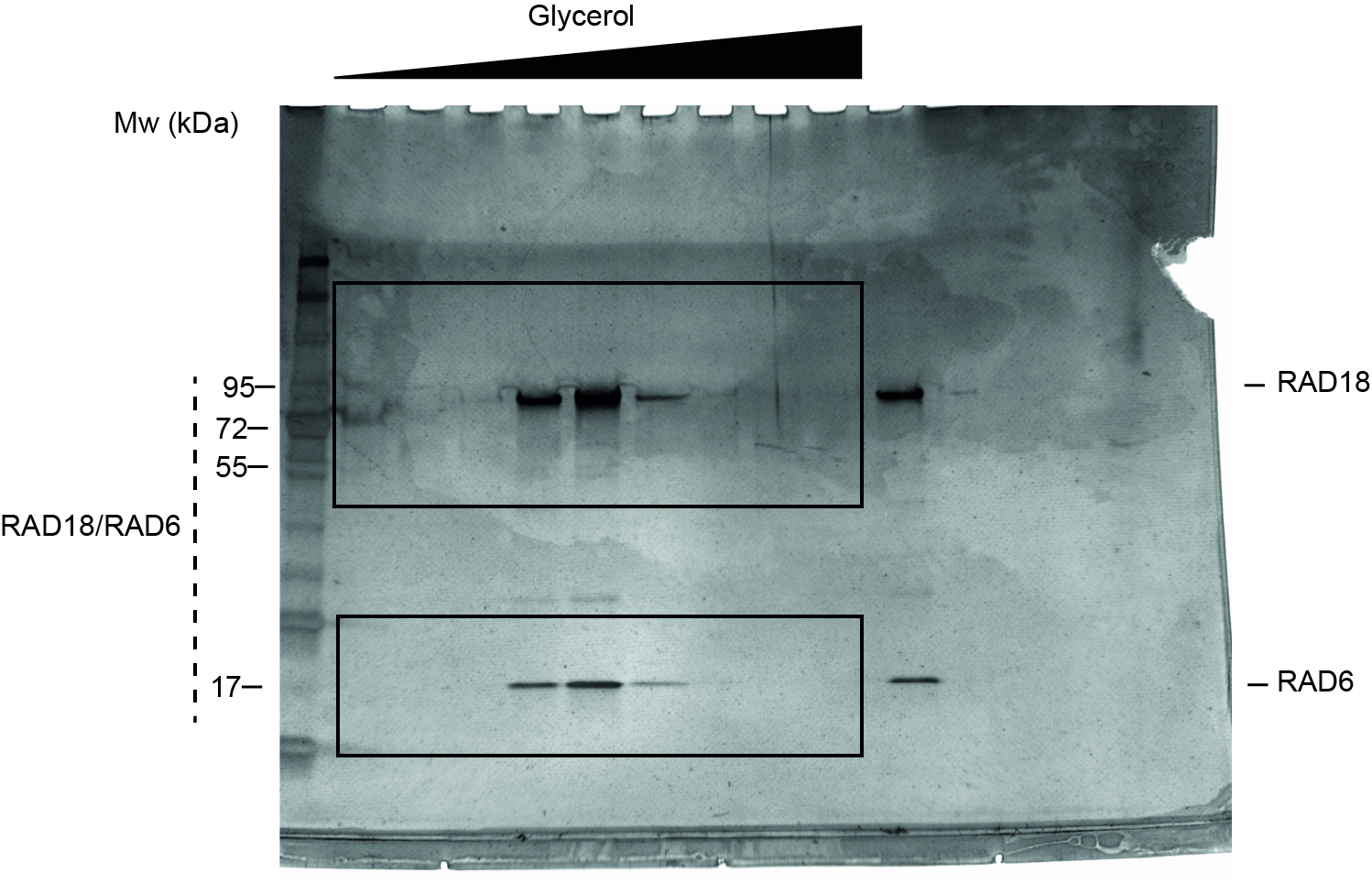

Supplement: Supplementary file 6 — Source Data Fig. 3 [file 44318_2024_58_MOESM6_ESM.zip › Figure 3/3D/RAD18RAD6Model.jpg]

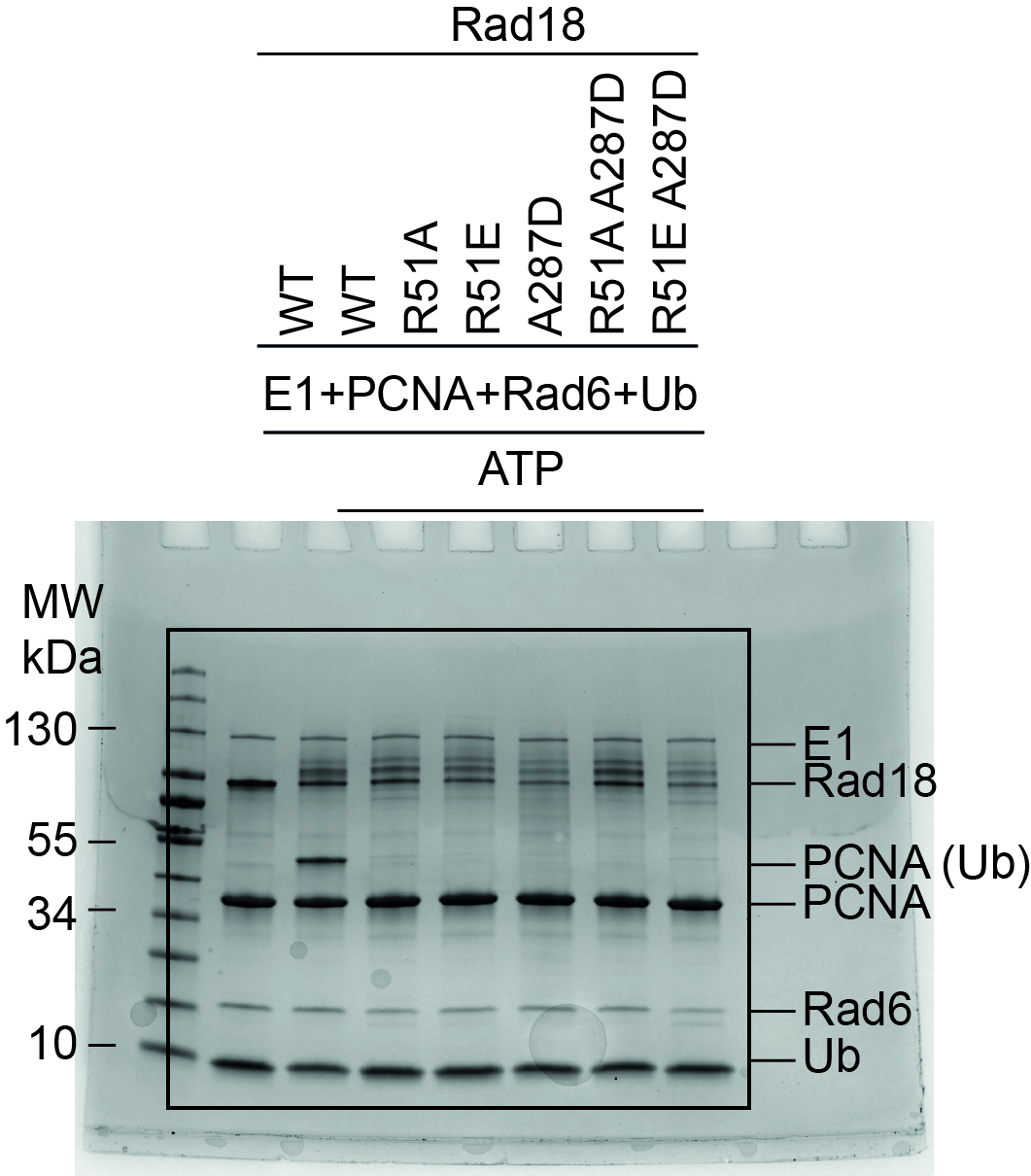

Supplement: Supplementary file 7 — Source Data Fig. 4 [file 44318_2024_58_MOESM7_ESM.zip › Figure 4/4E/CoomassieModel.jpg]

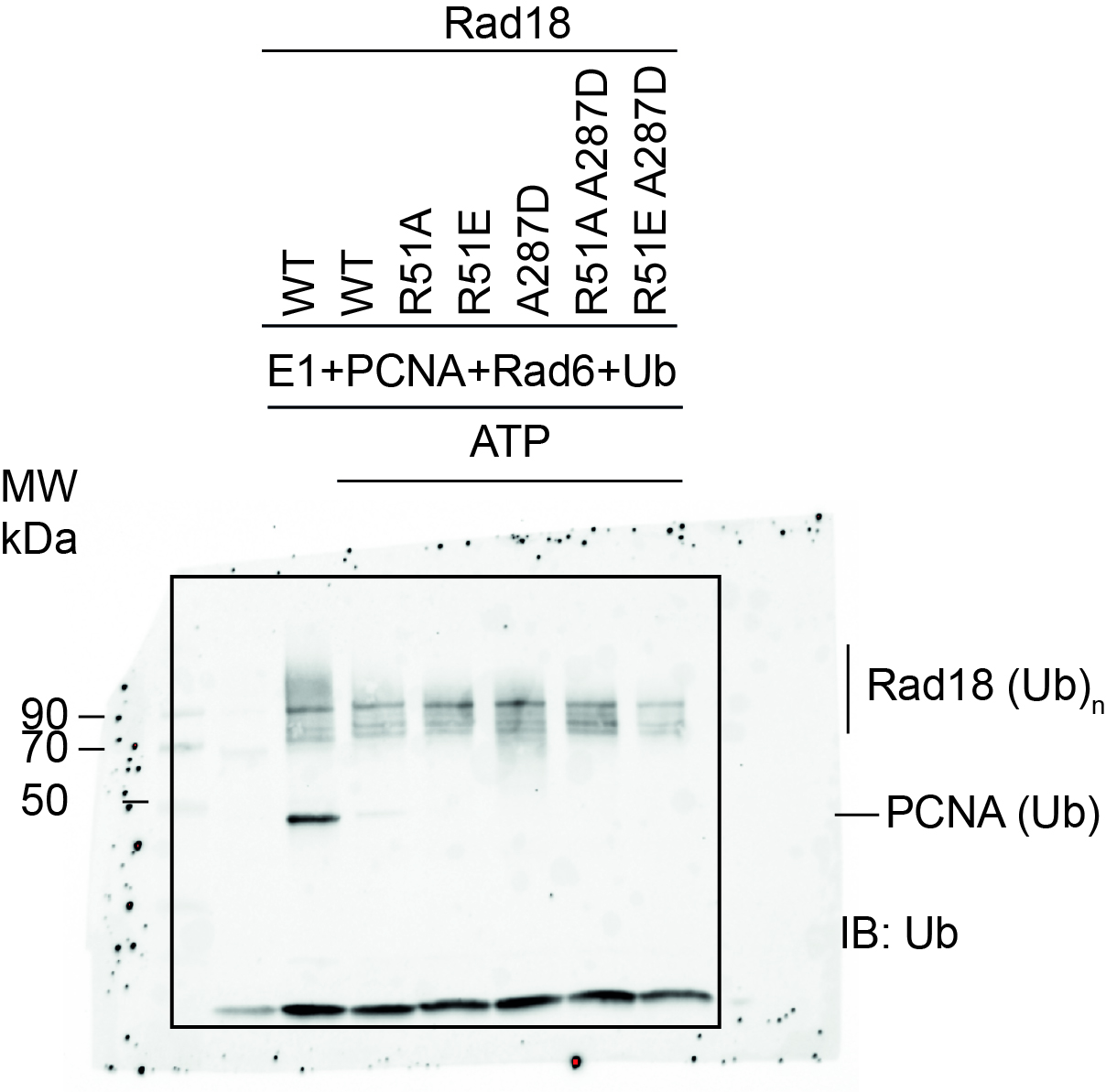

Supplement: Supplementary file 7 — Source Data Fig. 4 [file 44318_2024_58_MOESM7_ESM.zip › Figure 4/4E/Ub blotModel.jpg]

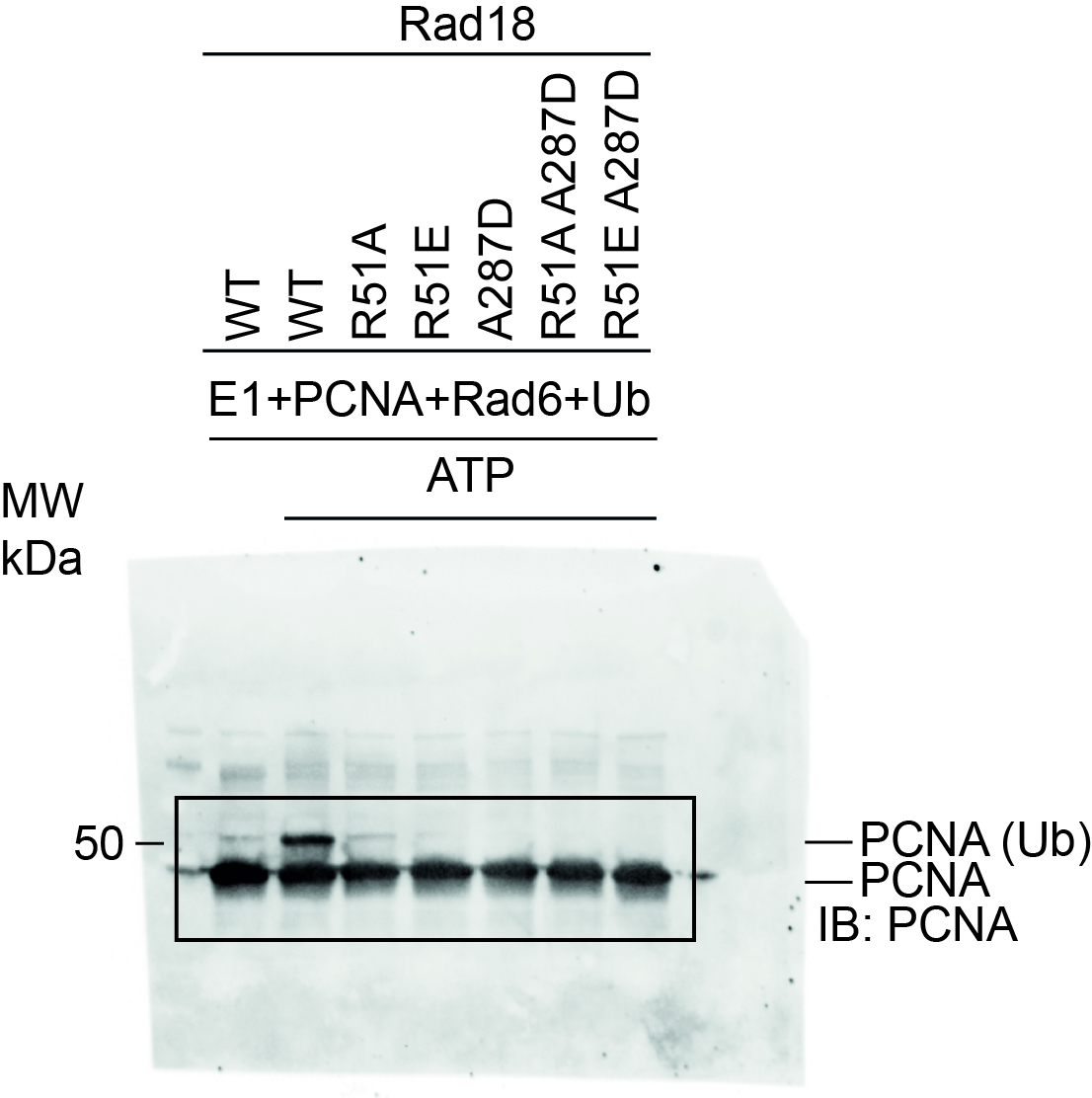

Supplement: Supplementary file 7 — Source Data Fig. 4 [file 44318_2024_58_MOESM7_ESM.zip › Figure 4/4E/PCNA blotModel.jpg]

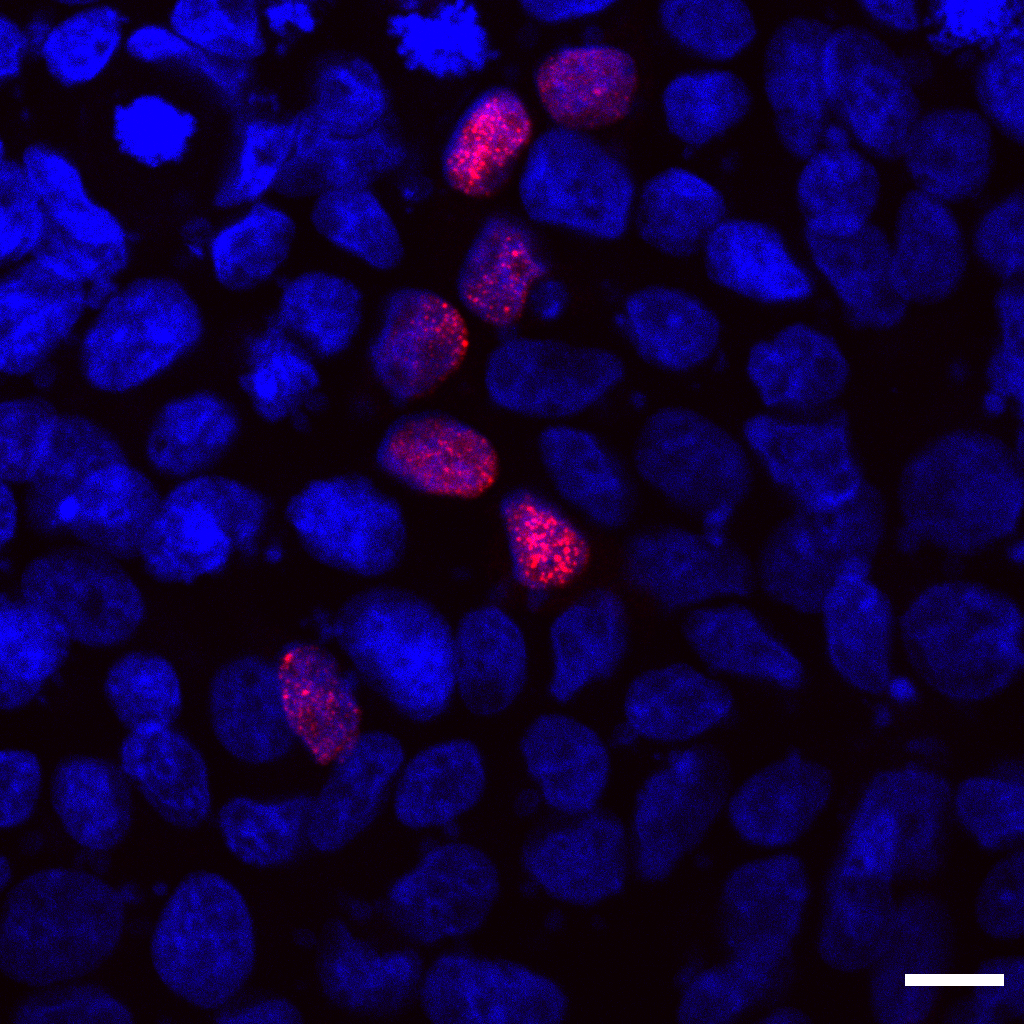

Supplement: Supplementary file 7 — Source Data Fig. 4 [file 44318_2024_58_MOESM7_ESM.zip › Figure 4/4F/RAD18+UV/Composite.png]

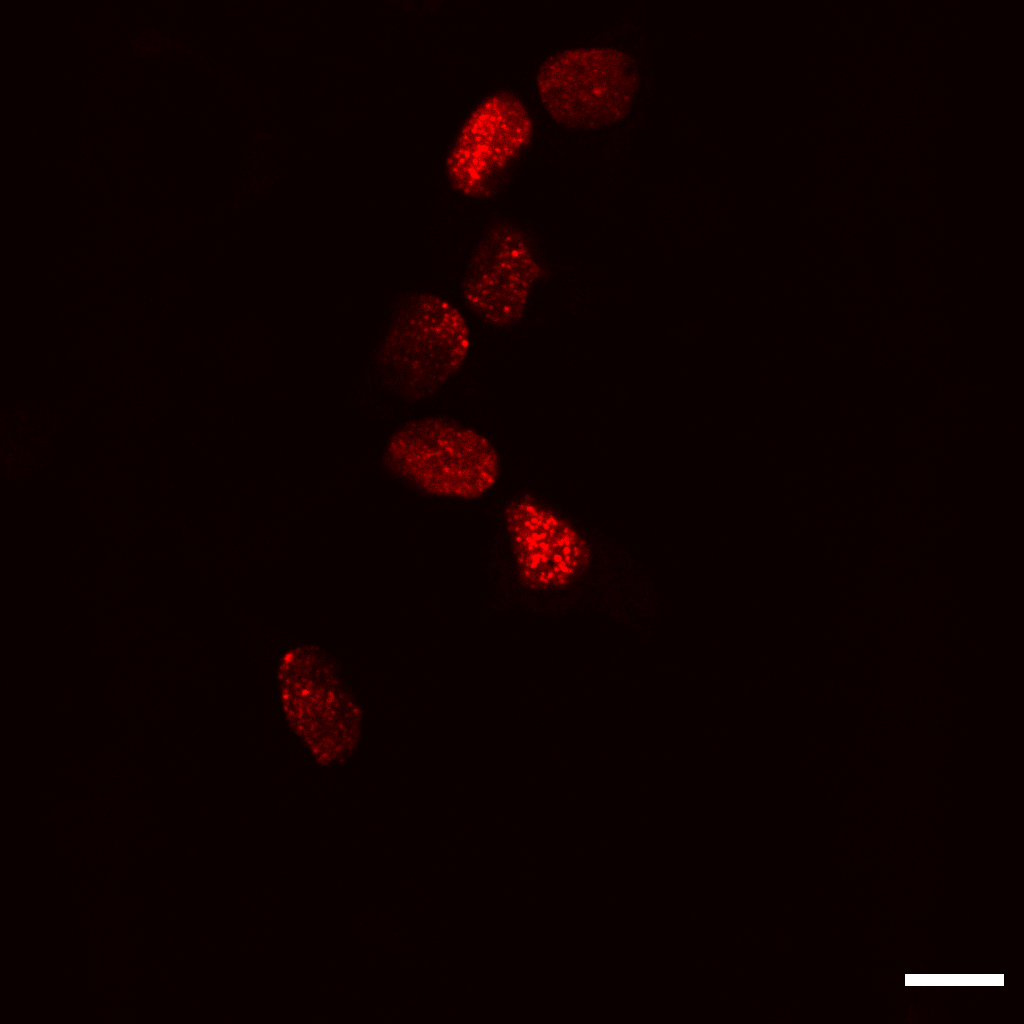

Supplement: Supplementary file 7 — Source Data Fig. 4 [file 44318_2024_58_MOESM7_ESM.zip › Figure 4/4F/RAD18+UV/C1-Composite.png]

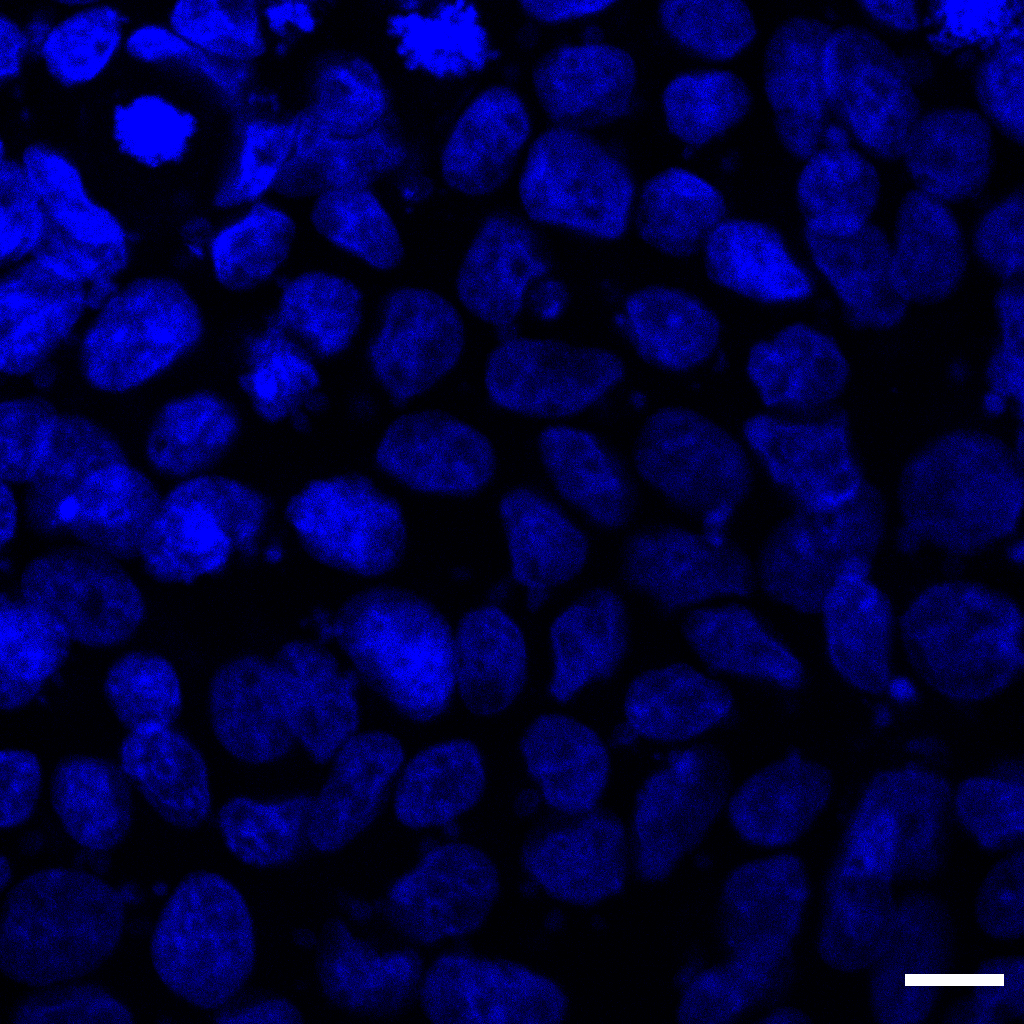

Supplement: Supplementary file 7 — Source Data Fig. 4 [file 44318_2024_58_MOESM7_ESM.zip › Figure 4/4F/RAD18+UV/C2-Composite.png]

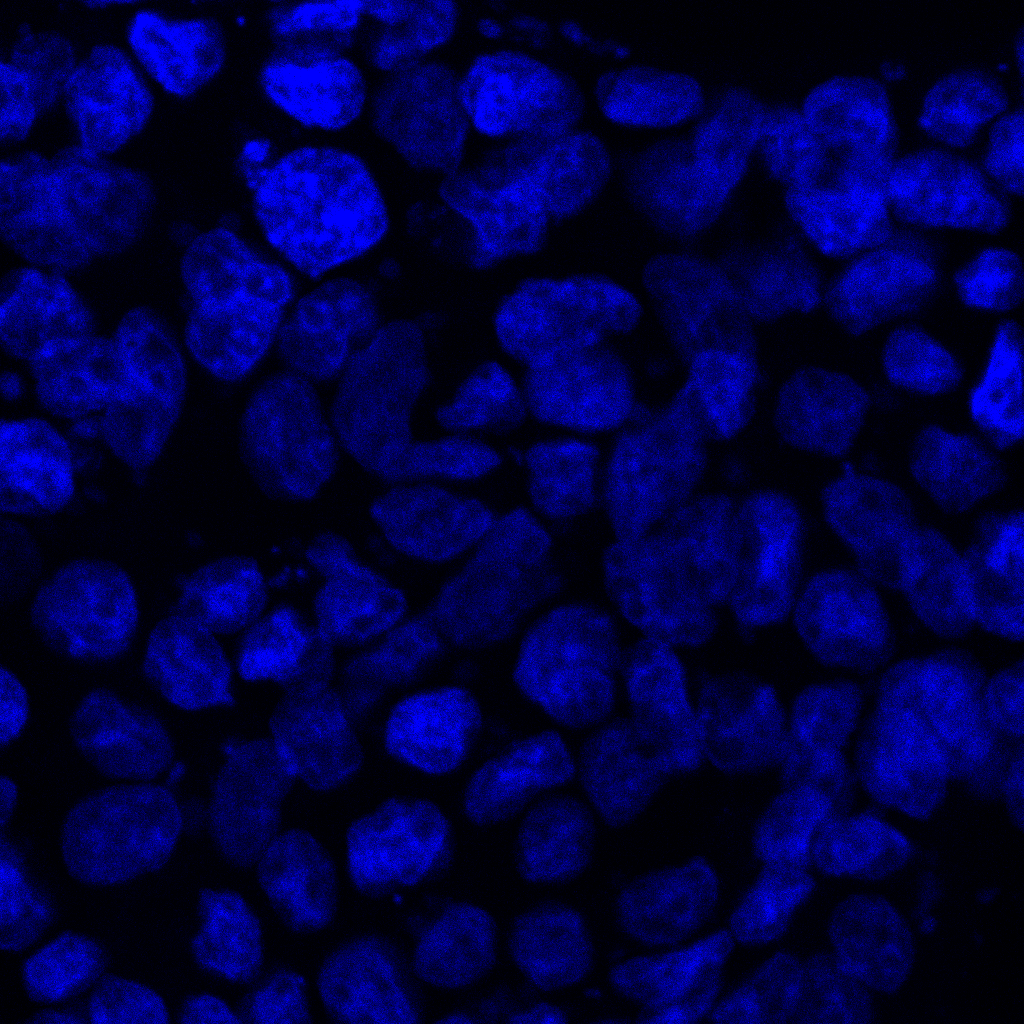

Supplement: Supplementary file 7 — Source Data Fig. 4 [file 44318_2024_58_MOESM7_ESM.zip › Figure 4/4F/R51E_+UV/C2-Composite.tif]

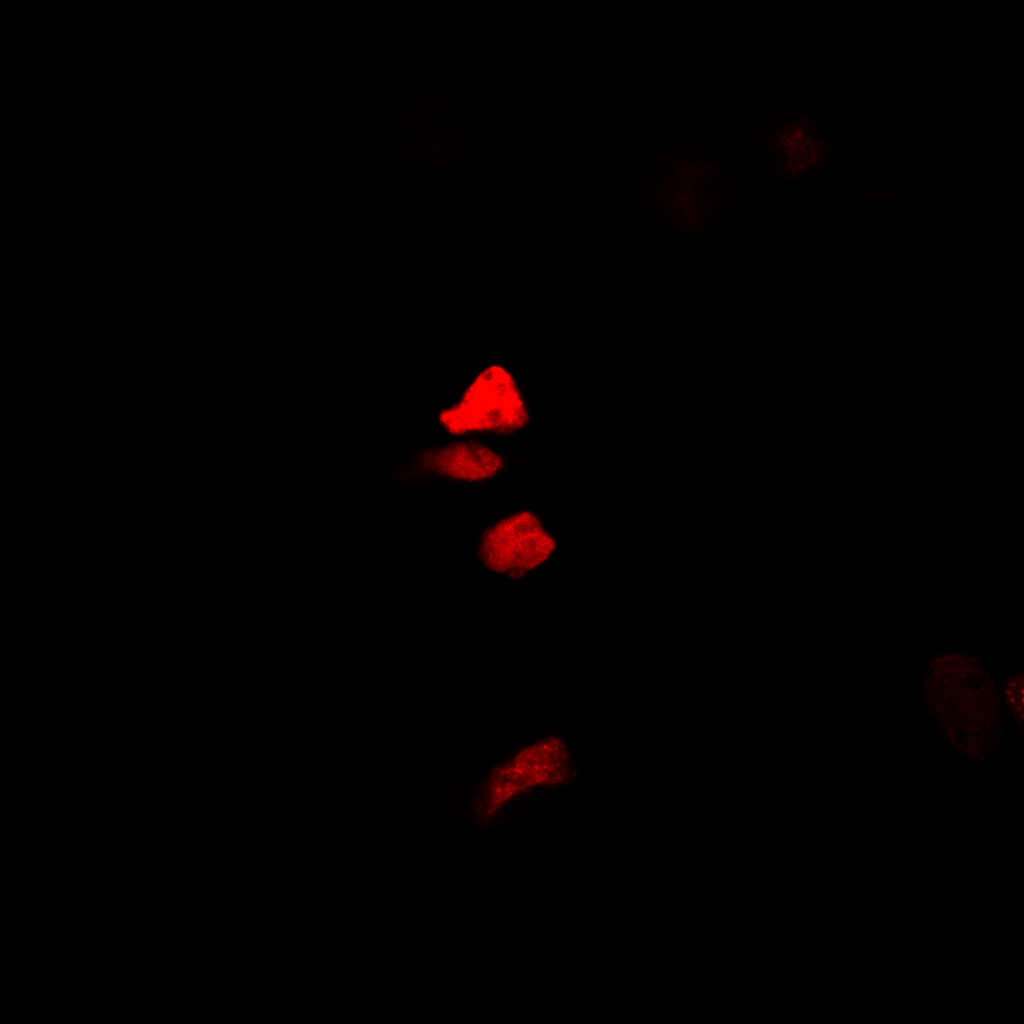

Supplement: Supplementary file 7 — Source Data Fig. 4 [file 44318_2024_58_MOESM7_ESM.zip › Figure 4/4F/R51E_+UV/C1-Composite.tif]

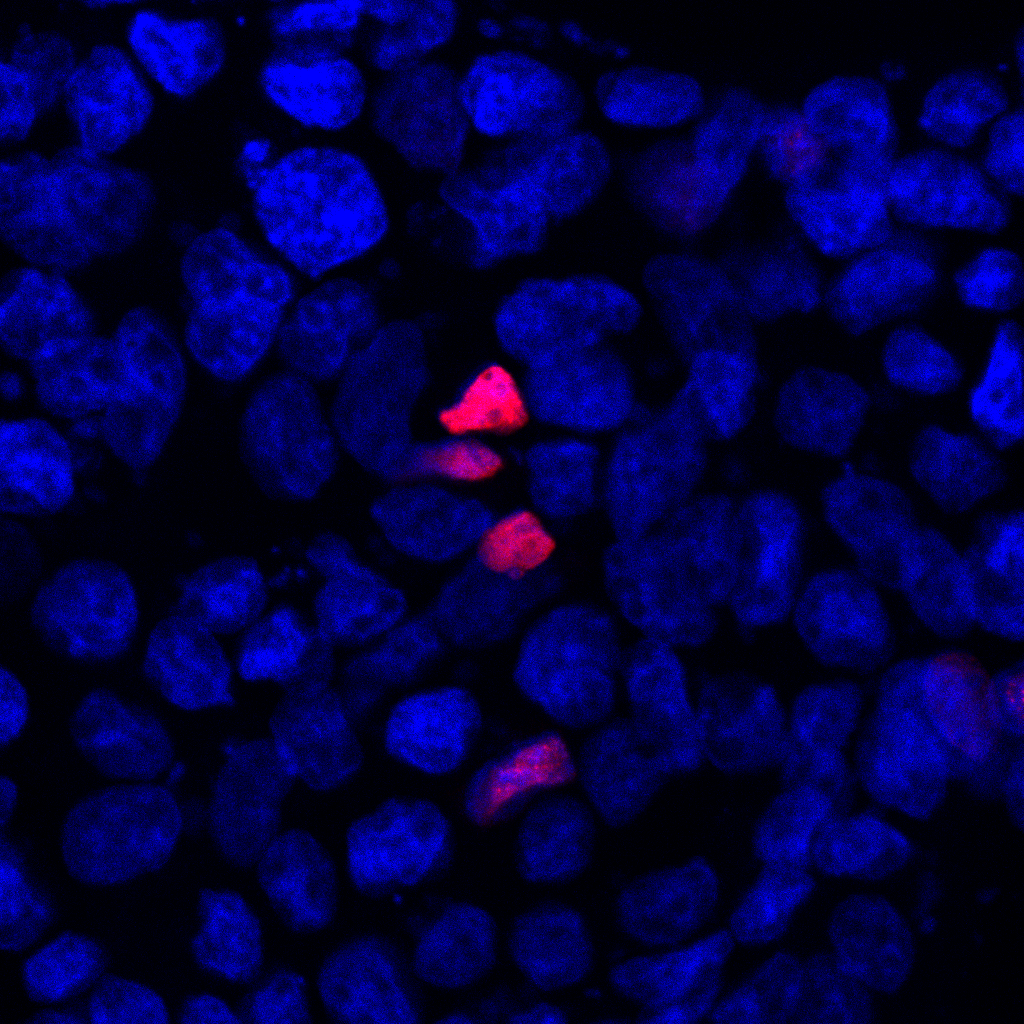

Supplement: Supplementary file 7 — Source Data Fig. 4 [file 44318_2024_58_MOESM7_ESM.zip › Figure 4/4F/R51E_+UV/Composite (RGB).tif]

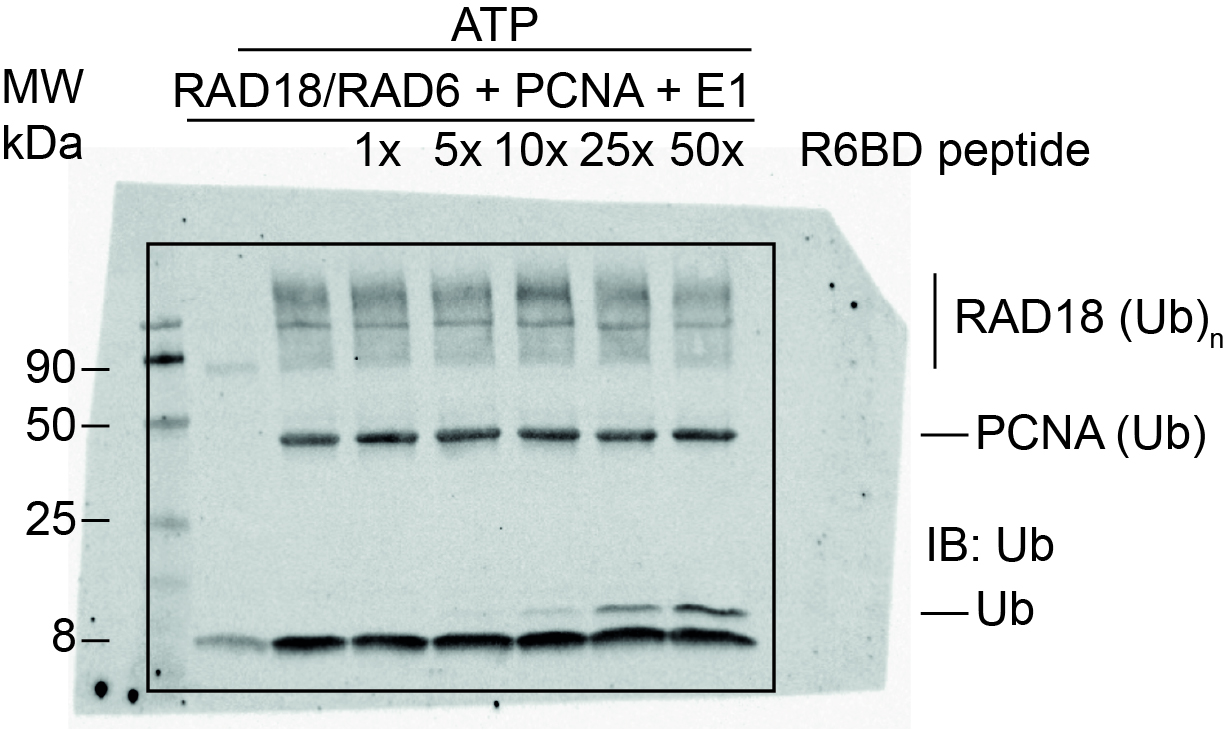

Supplement: Supplementary file 8 — Source Data Fig. 5 [file 44318_2024_58_MOESM8_ESM.zip › Figure 5/5G/Ub_BlotModel.jpg]

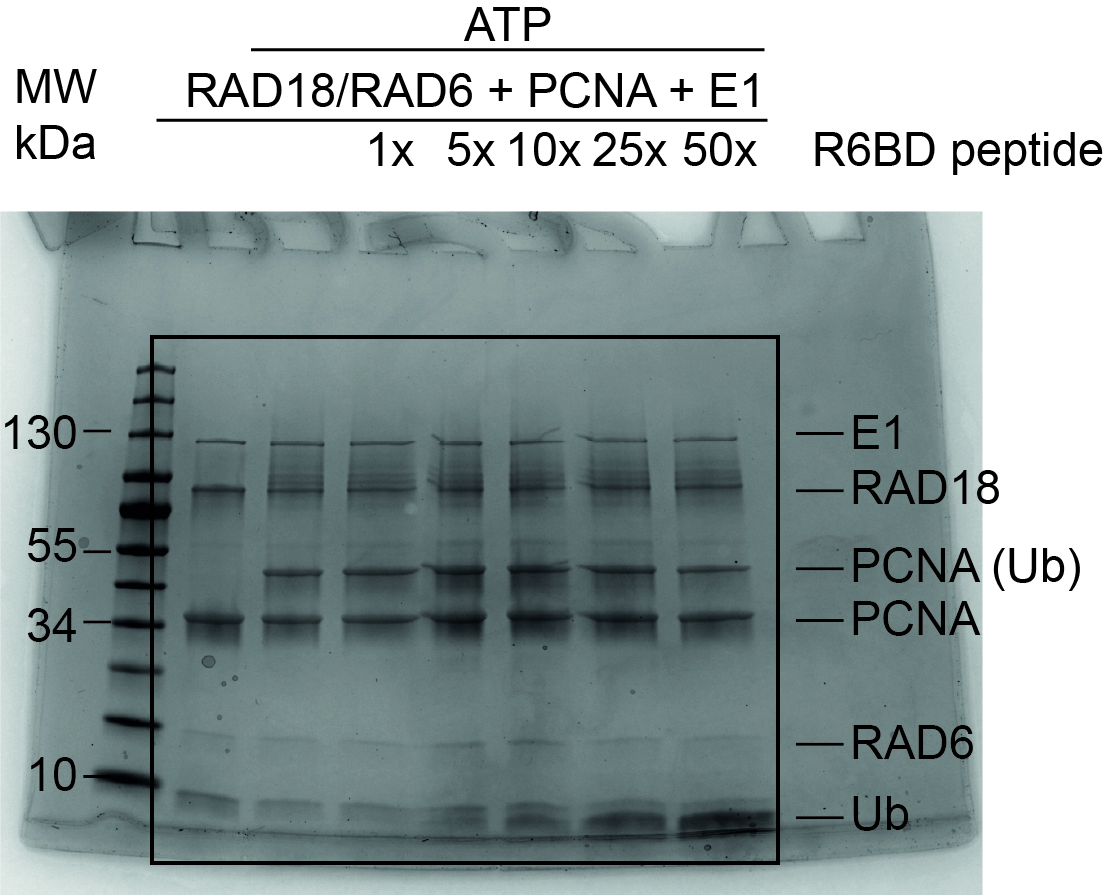

Supplement: Supplementary file 8 — Source Data Fig. 5 [file 44318_2024_58_MOESM8_ESM.zip › Figure 5/5G/CoomassieModel.jpg]

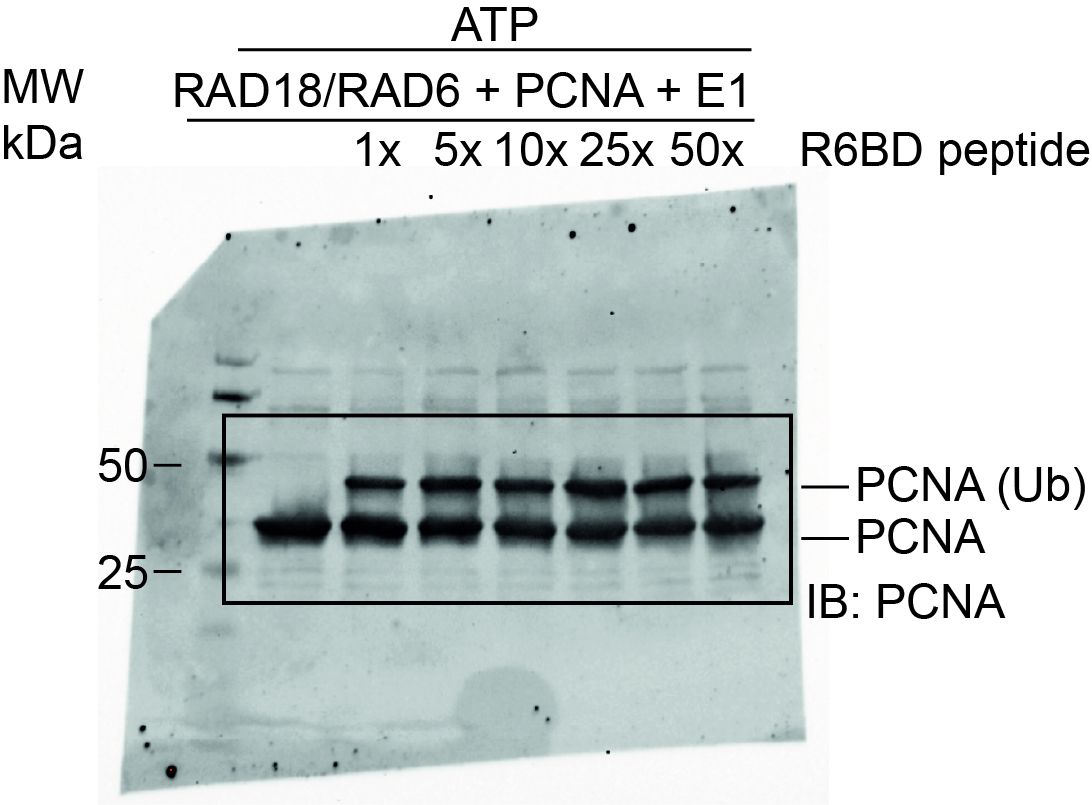

Supplement: Supplementary file 8 — Source Data Fig. 5 [file 44318_2024_58_MOESM8_ESM.zip › Figure 5/5G/PCNA_BlotModel.jpg]

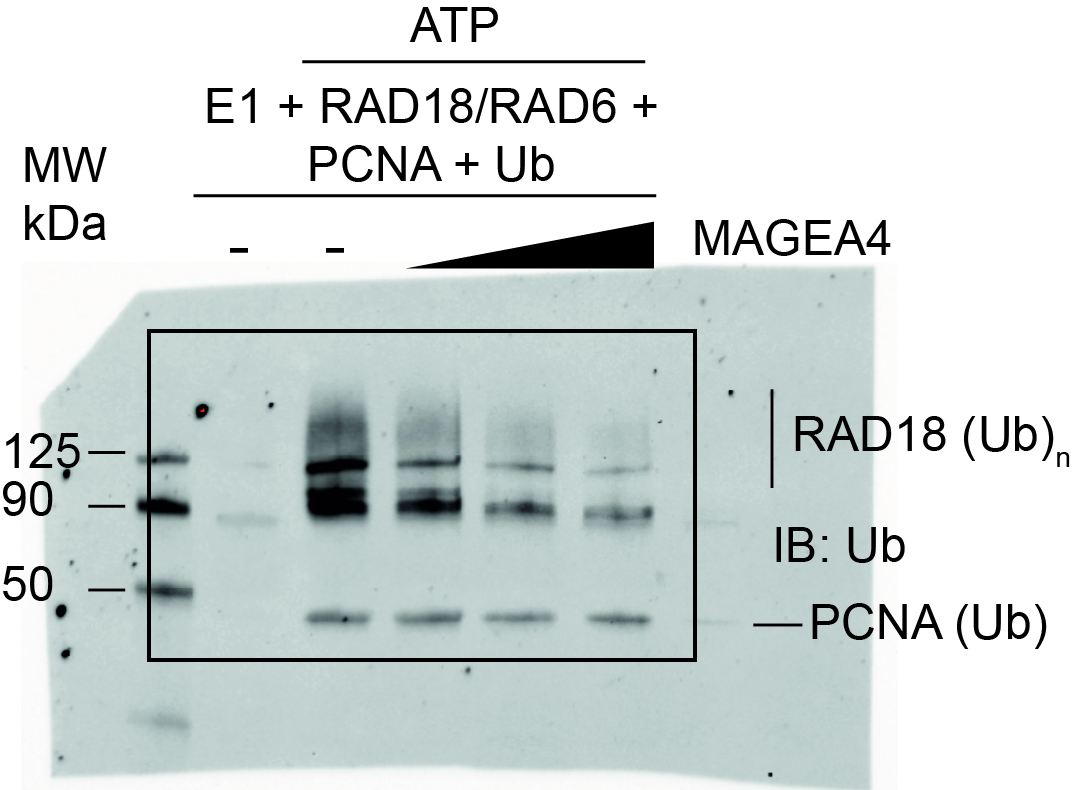

Supplement: Supplementary file 8 — Source Data Fig. 5 [file 44318_2024_58_MOESM8_ESM.zip › Figure 5/5A/Ub_blotModel.jpg]

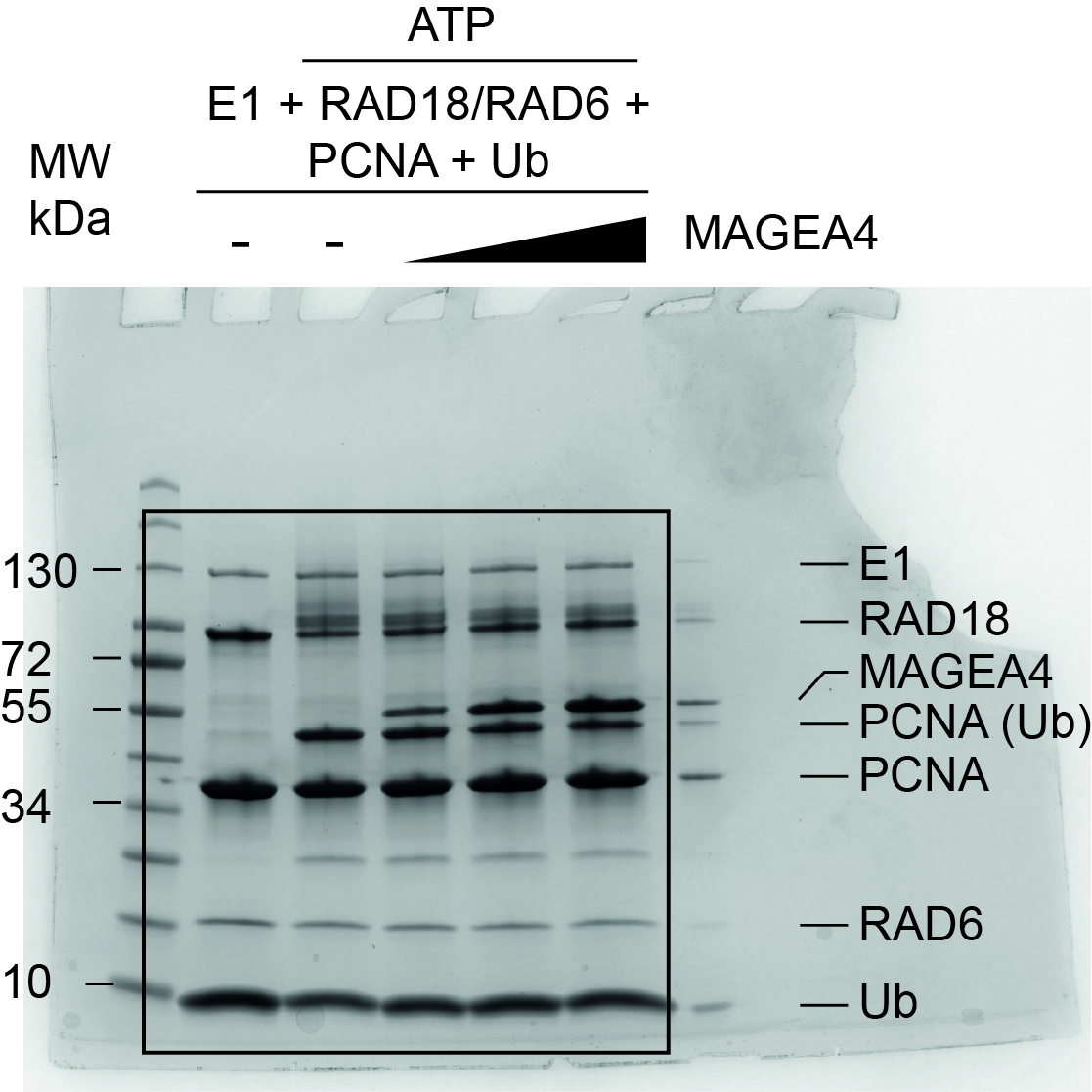

Supplement: Supplementary file 8 — Source Data Fig. 5 [file 44318_2024_58_MOESM8_ESM.zip › Figure 5/5A/CoomassieModel.jpg]

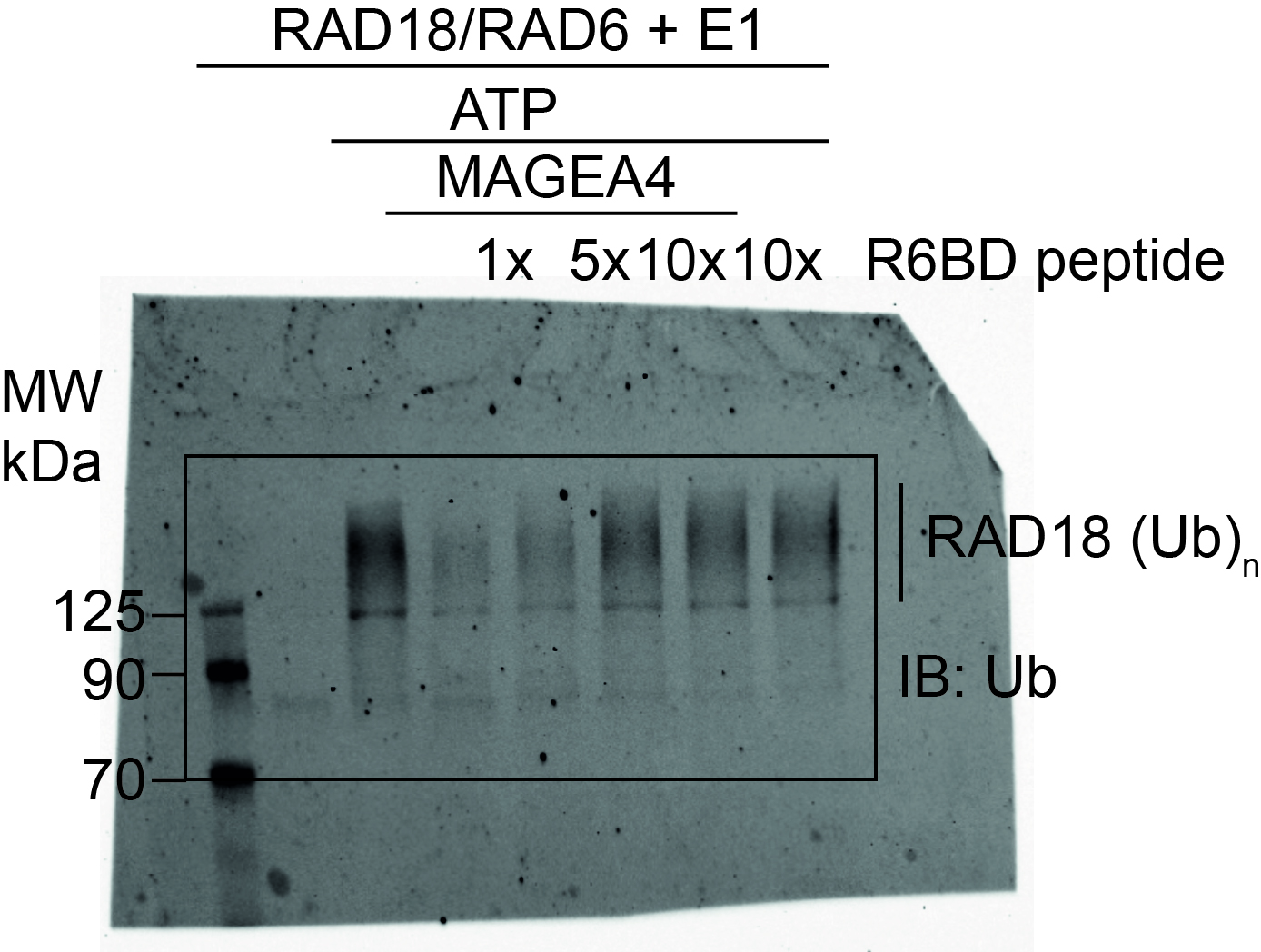

Supplement: Supplementary file 8 — Source Data Fig. 5 [file 44318_2024_58_MOESM8_ESM.zip › Figure 5/5F/Ub_BlotModel.jpg]

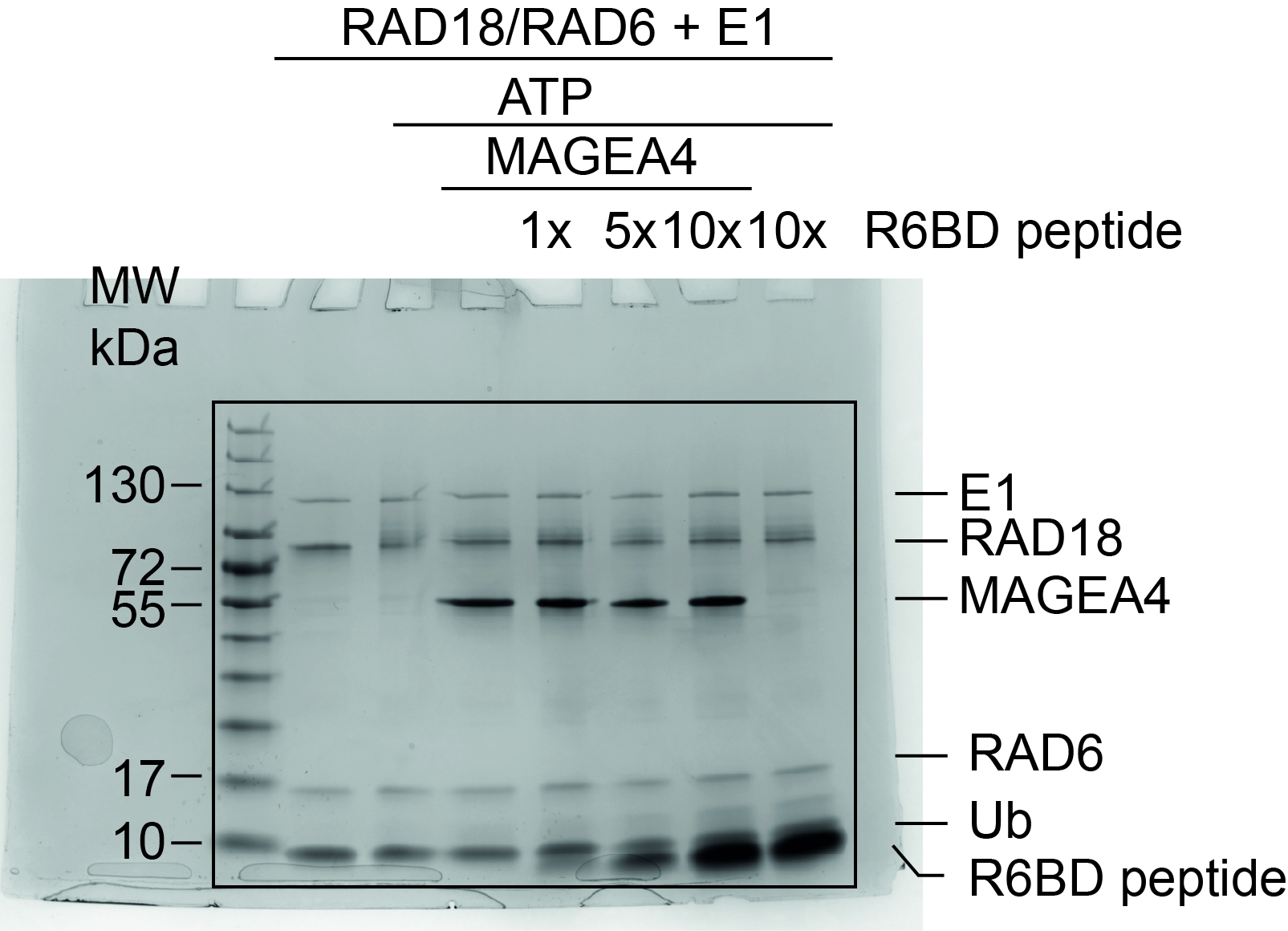

Supplement: Supplementary file 8 — Source Data Fig. 5 [file 44318_2024_58_MOESM8_ESM.zip › Figure 5/5F/CoomassieModel.jpg]

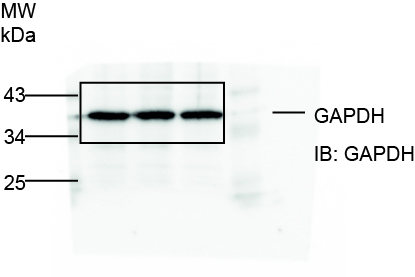

Supplement: Supplementary file 8 — Source Data Fig. 5 [file 44318_2024_58_MOESM8_ESM.zip › Figure 5/5C/GAPDH_BlotModel.jpg]

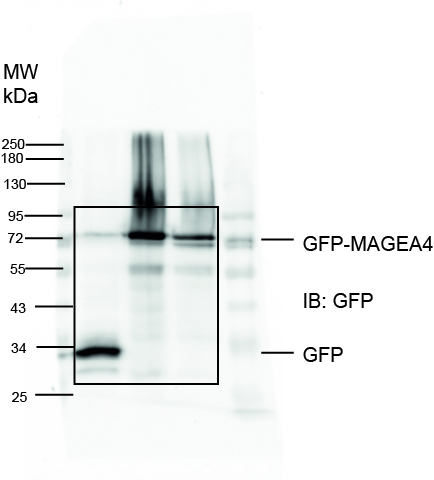

Supplement: Supplementary file 8 — Source Data Fig. 5 [file 44318_2024_58_MOESM8_ESM.zip › Figure 5/5C/GFP-BlotModel.jpg]

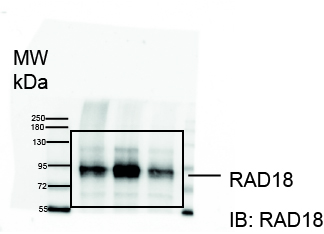

Supplement: Supplementary file 8 — Source Data Fig. 5 [file 44318_2024_58_MOESM8_ESM.zip › Figure 5/5C/RAD18_BlotModel.jpg]

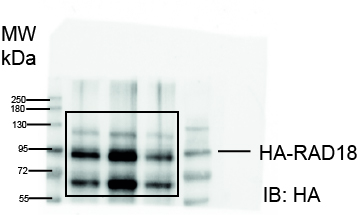

Supplement: Supplementary file 8 — Source Data Fig. 5 [file 44318_2024_58_MOESM8_ESM.zip › Figure 5/5C/HA_BlotModel.jpg]

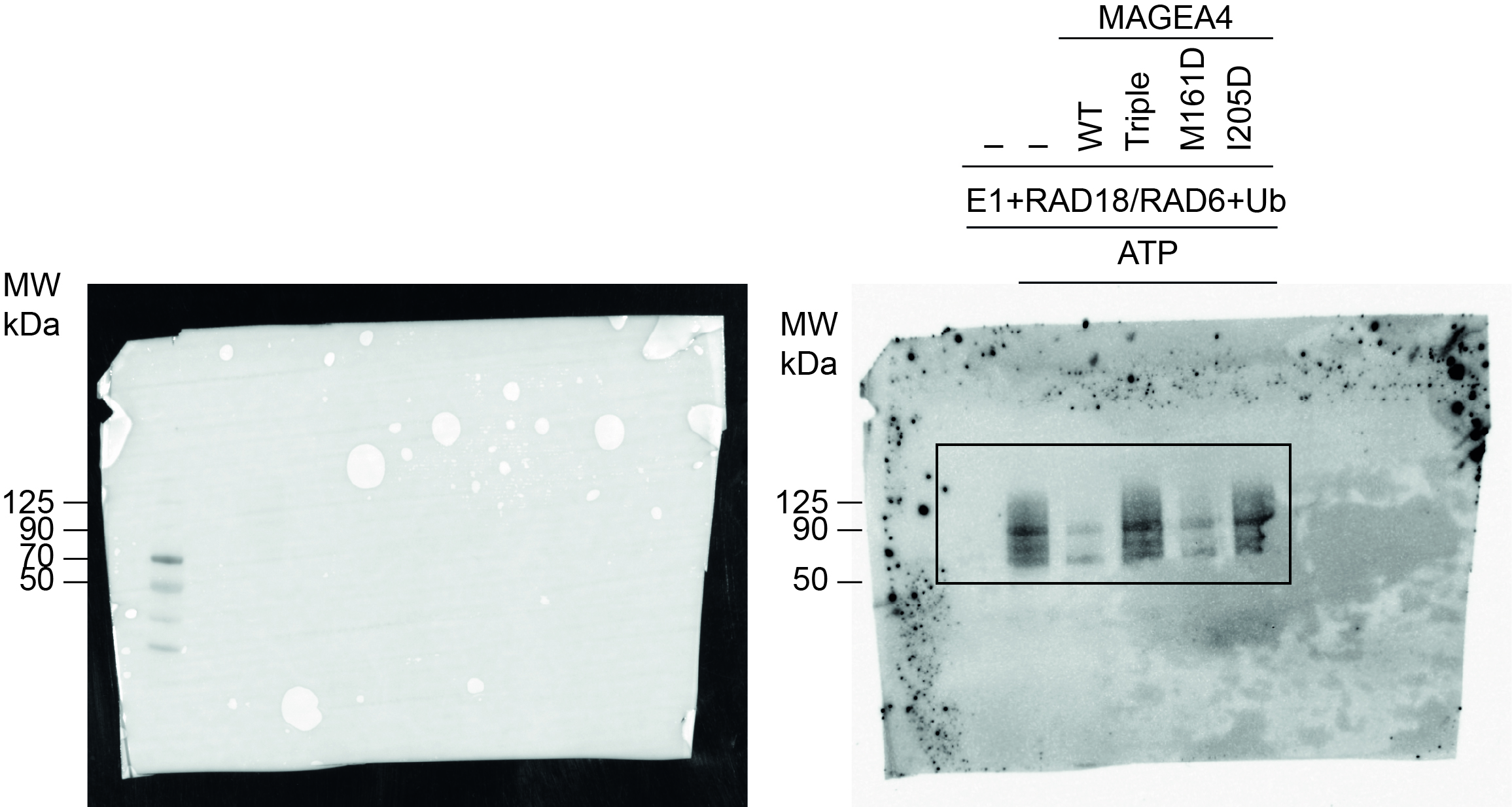

Supplement: Supplementary file 8 — Source Data Fig. 5 [file 44318_2024_58_MOESM8_ESM.zip › Figure 5/5B/Ub_BlotModel.jpg]

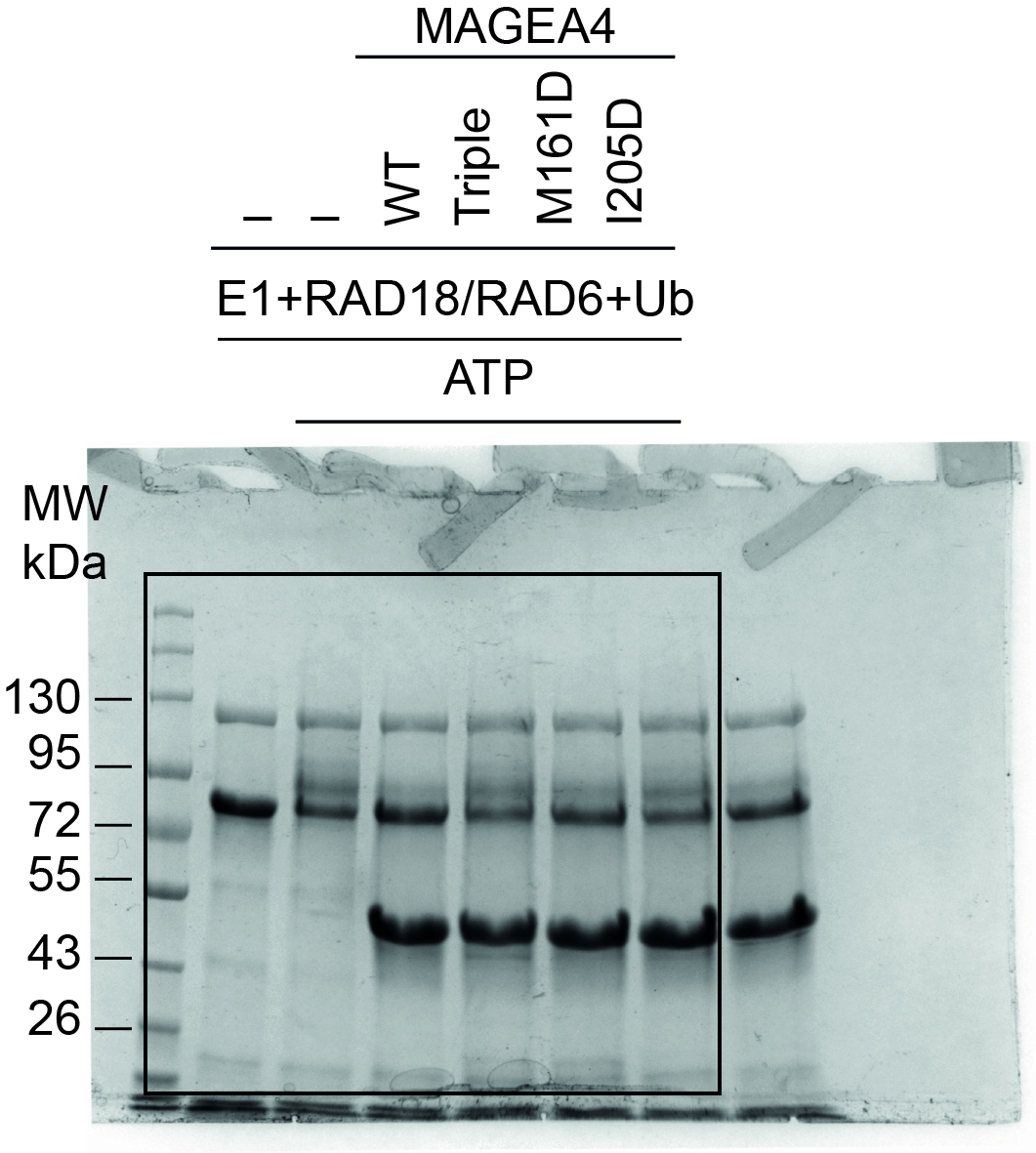

Supplement: Supplementary file 8 — Source Data Fig. 5 [file 44318_2024_58_MOESM8_ESM.zip › Figure 5/5B/CoomassieModel.jpg]

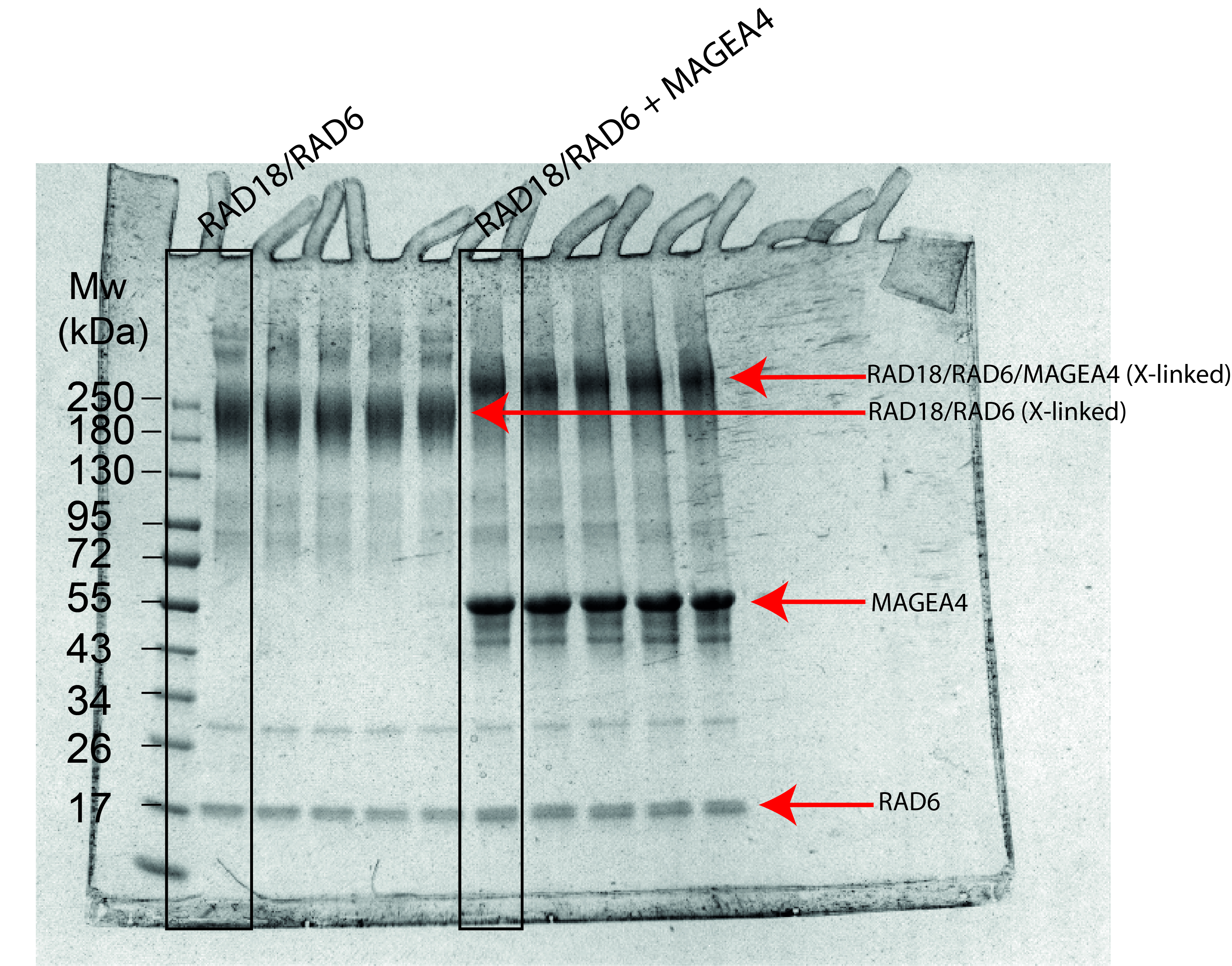

Supplement: Supplementary file 9 — EV Figures Source Data [file 44318_2024_58_MOESM9_ESM.zip › 2 EV Figures/EV2/EV2B/XLINK_RAD18RAD6_RAD18RAD6MAGEA4Model.jpg]

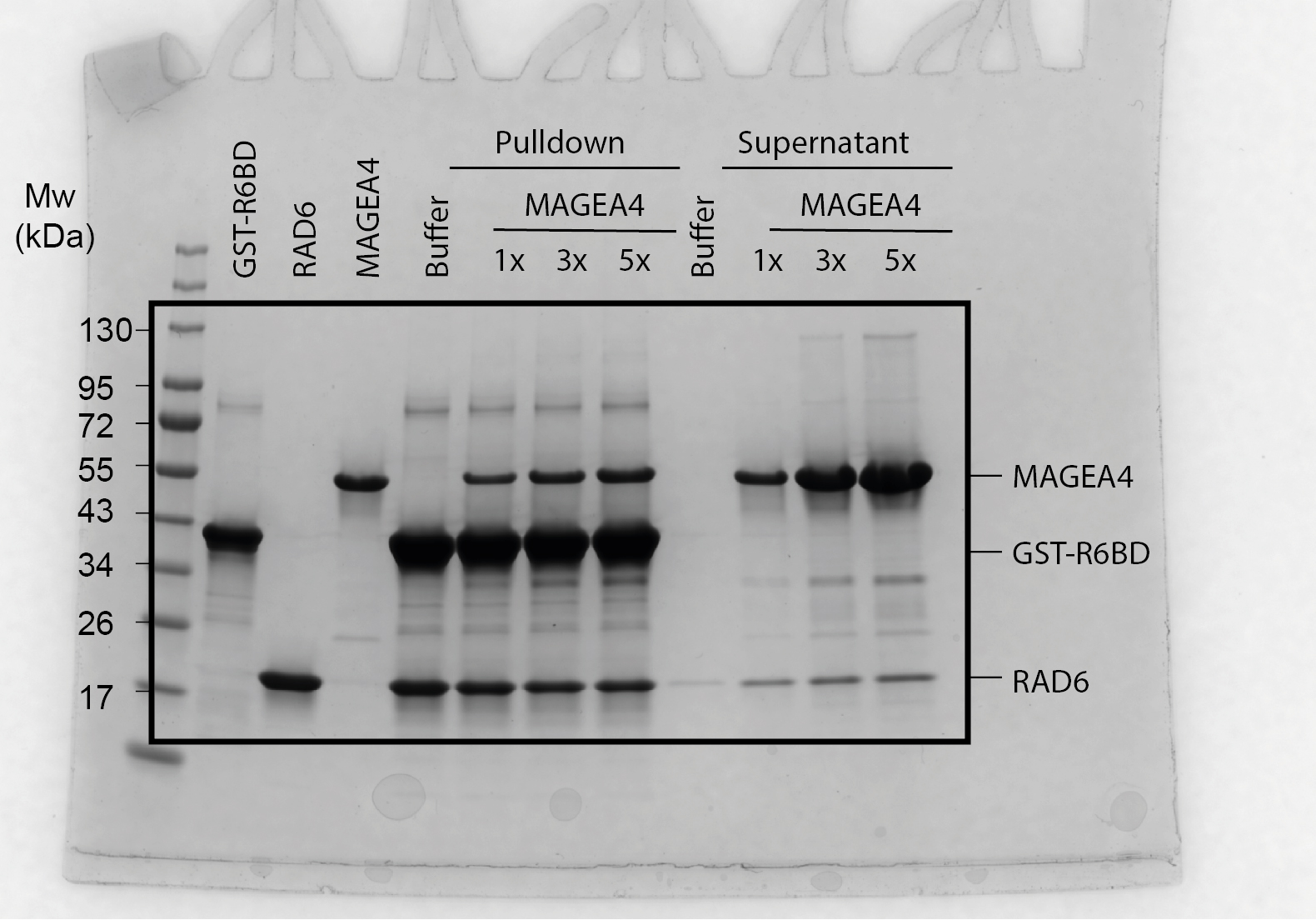

Supplement: Supplementary file 9 — EV Figures Source Data [file 44318_2024_58_MOESM9_ESM.zip › 2 EV Figures/EV2/EV2A/GST-pulldown.jpg]

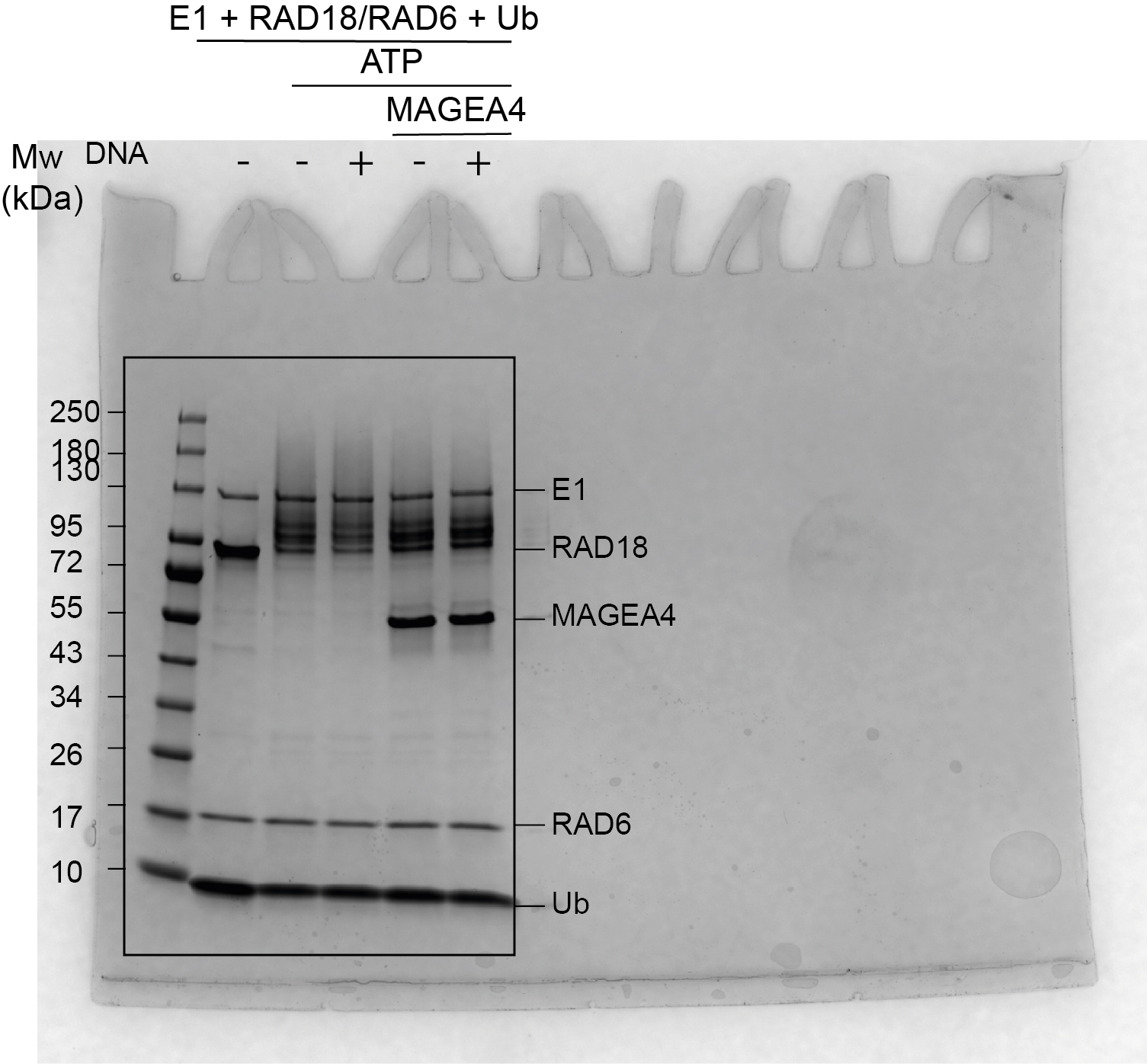

Supplement: Supplementary file 9 — EV Figures Source Data [file 44318_2024_58_MOESM9_ESM.zip › 2 EV Figures/EV4/EV4A/Coomassie.jpg]

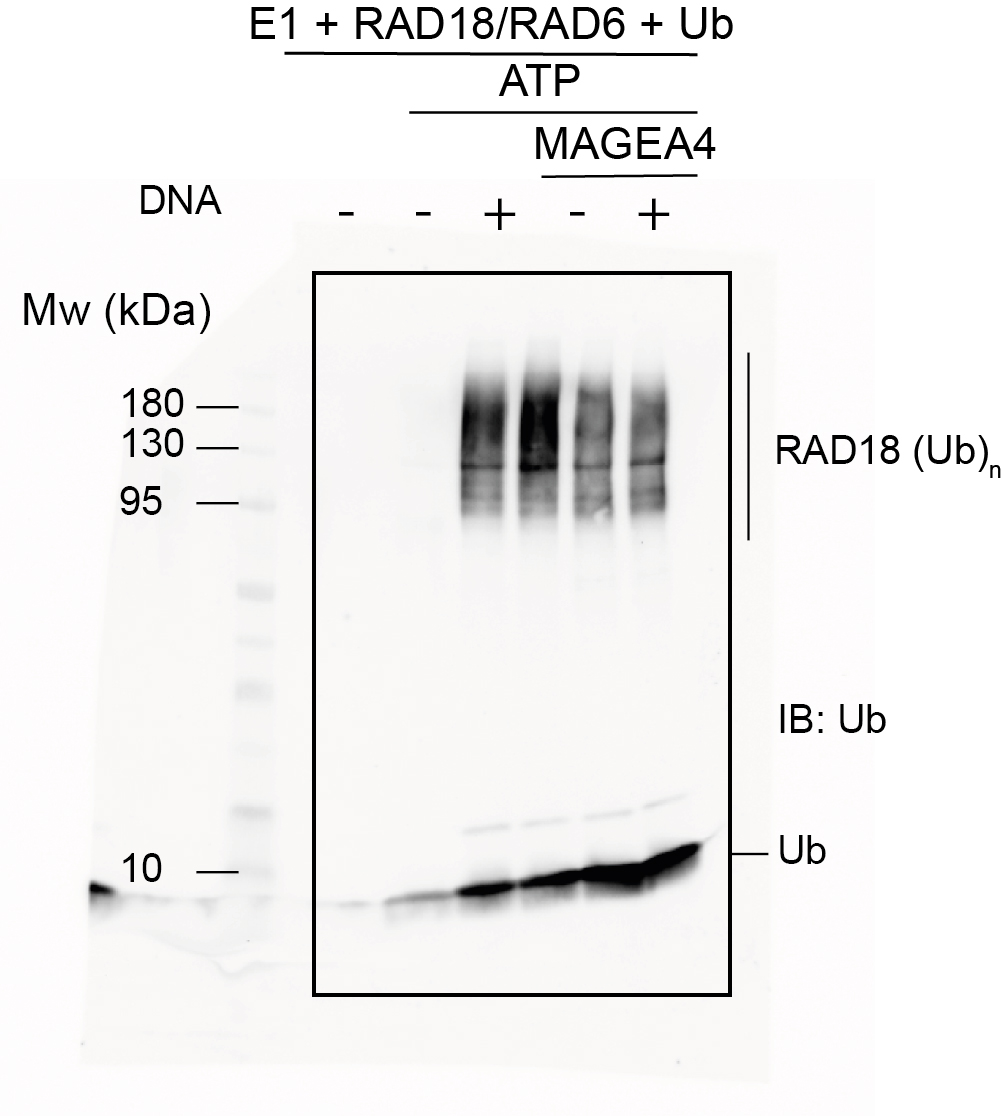

Supplement: Supplementary file 9 — EV Figures Source Data [file 44318_2024_58_MOESM9_ESM.zip › 2 EV Figures/EV4/EV4A/Ub_Blot.jpg]

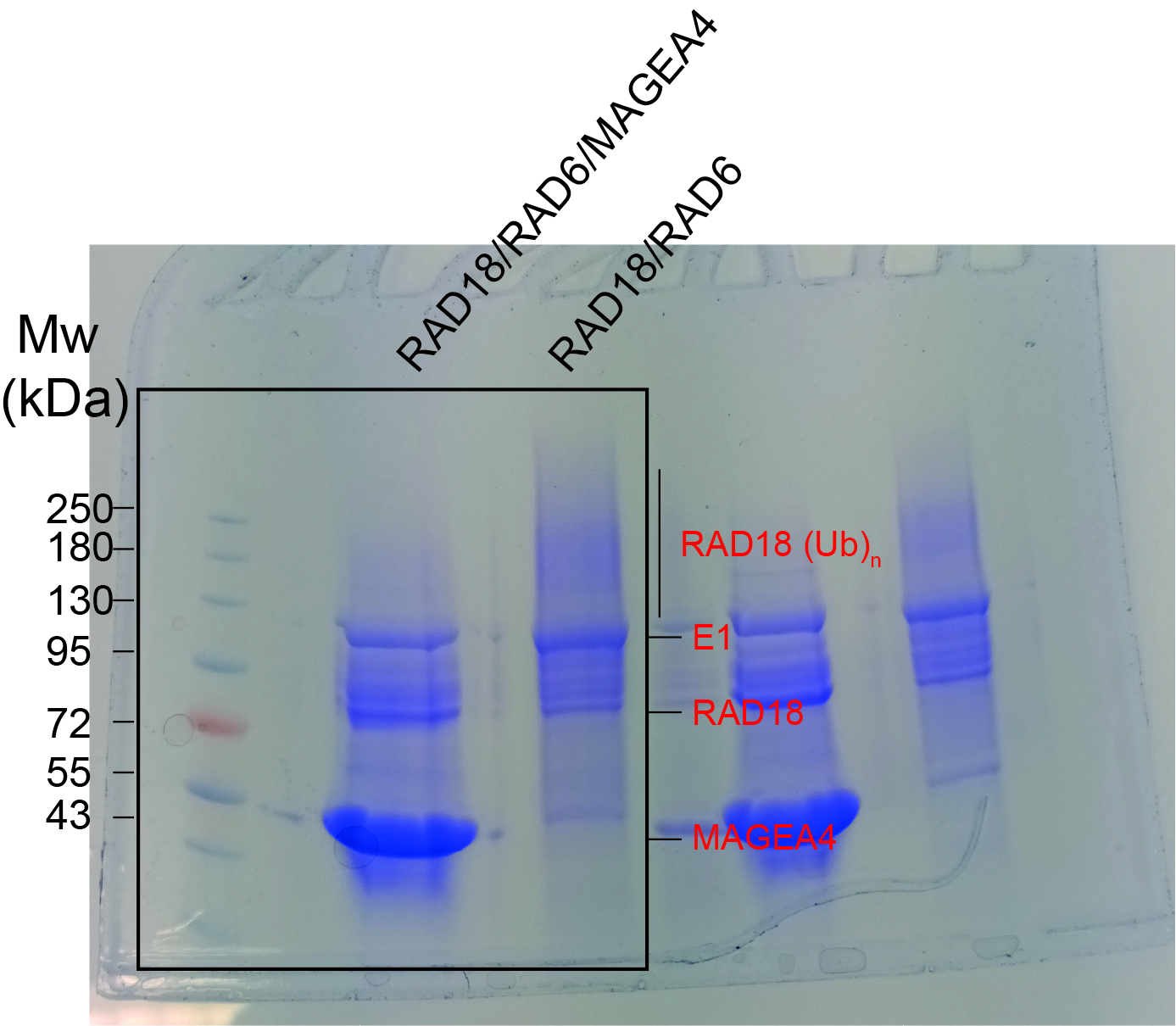

Supplement: Supplementary file 9 — EV Figures Source Data [file 44318_2024_58_MOESM9_ESM.zip › 2 EV Figures/EV4/EV4C/MS_Auto_Ub_GelModel.jpg]

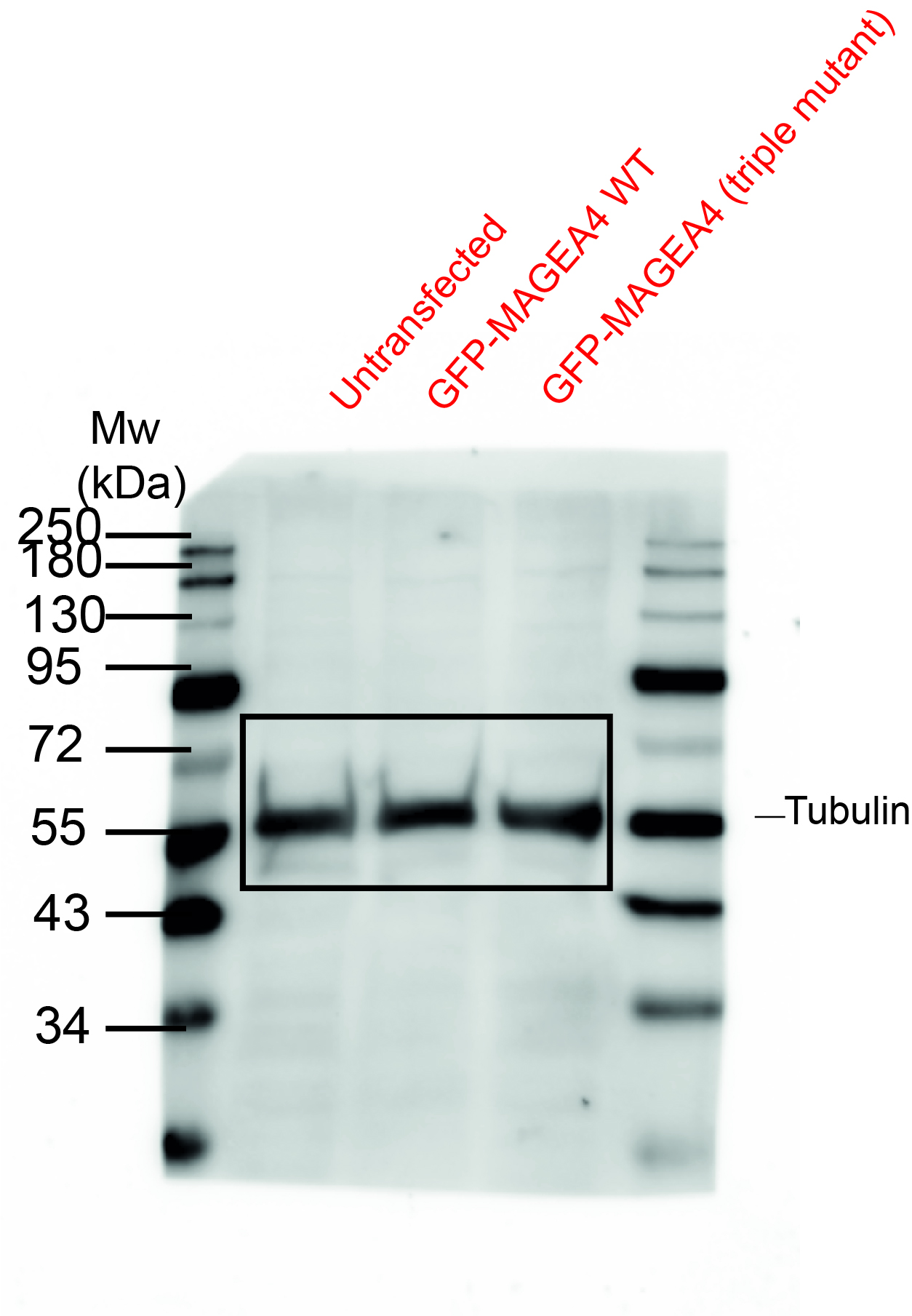

Supplement: Supplementary file 9 — EV Figures Source Data [file 44318_2024_58_MOESM9_ESM.zip › 2 EV Figures/EV4/EV4B/HEK_Cells_TublinModel.jpg]

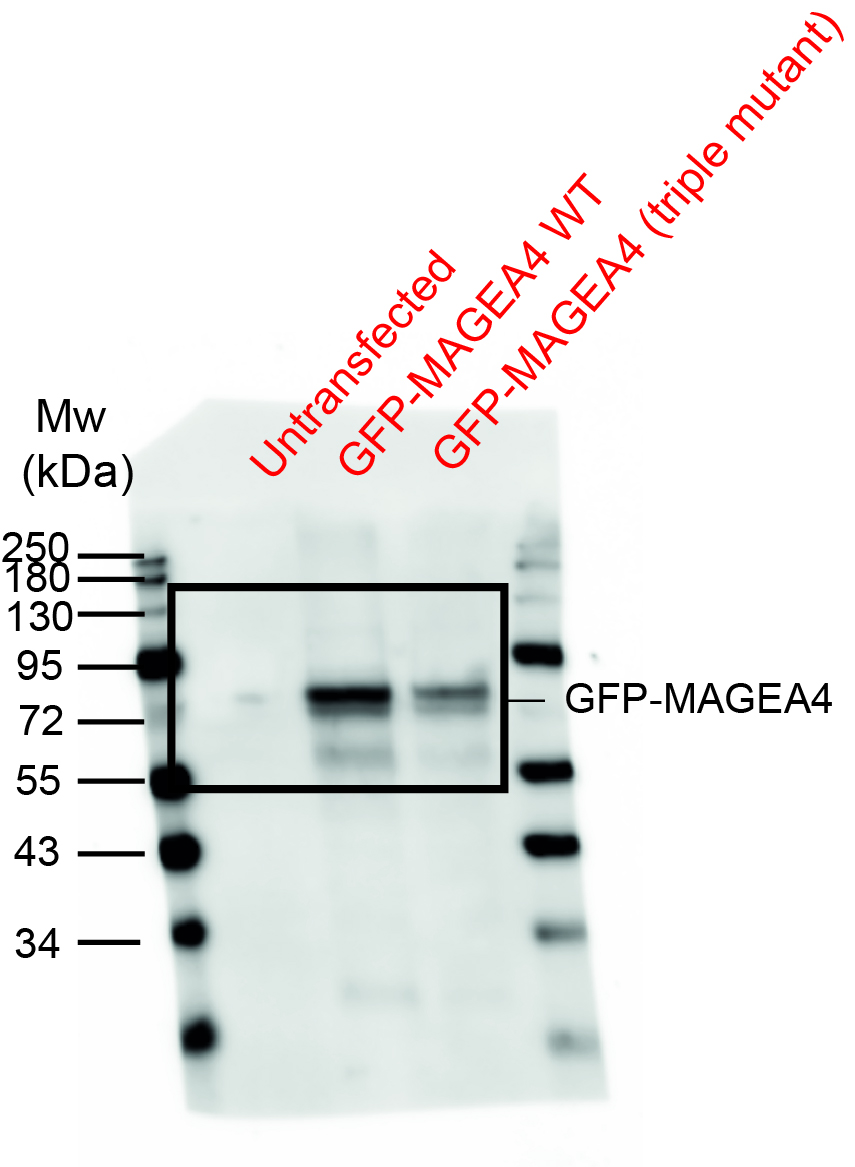

Supplement: Supplementary file 9 — EV Figures Source Data [file 44318_2024_58_MOESM9_ESM.zip › 2 EV Figures/EV4/EV4B/HEK_Cells_MAGEModel.jpg]

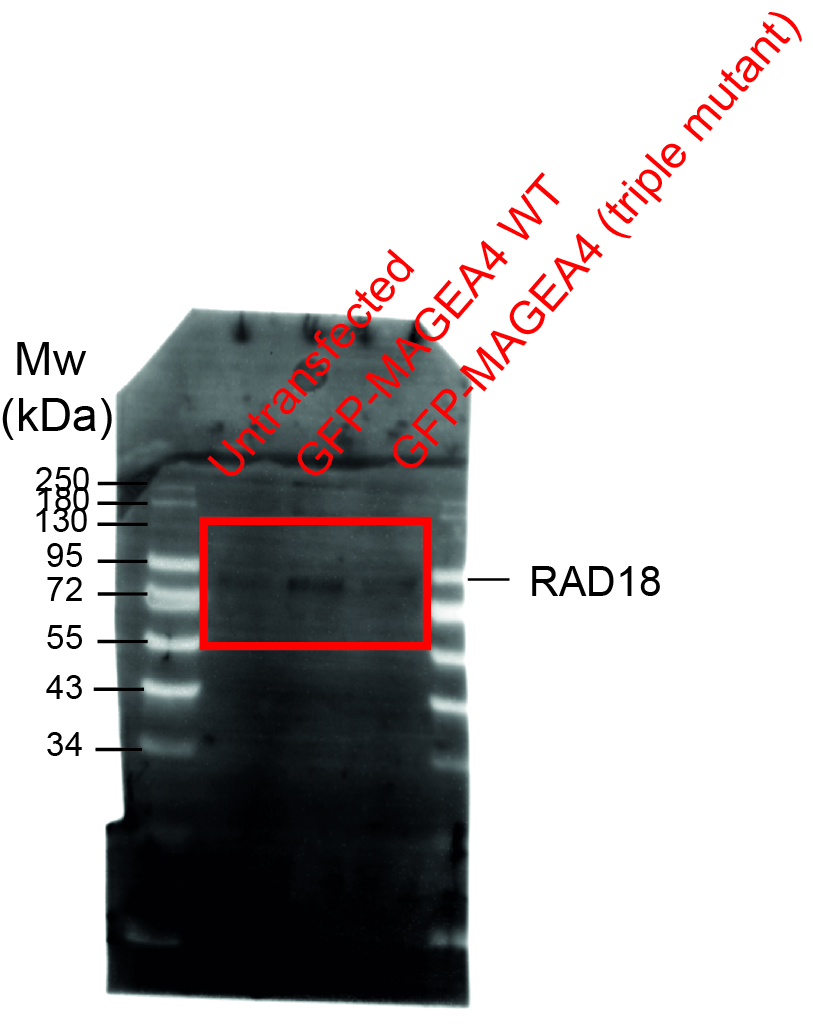

Supplement: Supplementary file 9 — EV Figures Source Data [file 44318_2024_58_MOESM9_ESM.zip › 2 EV Figures/EV4/EV4B/Endogenous_RAD18_RAD18Model.jpg]

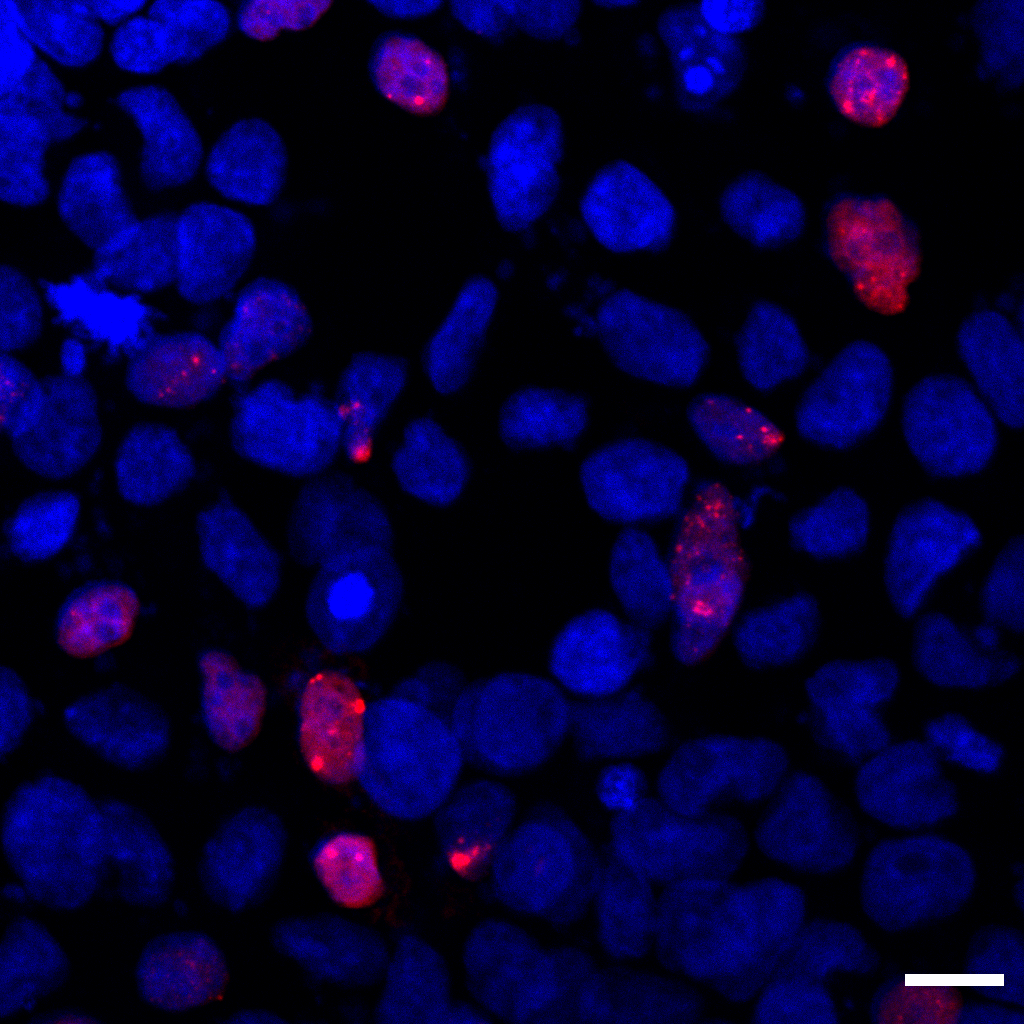

Supplement: Supplementary file 9 — EV Figures Source Data [file 44318_2024_58_MOESM9_ESM.zip › 2 EV Figures/EV3/EV3C/R51E-UV/Composite.png]

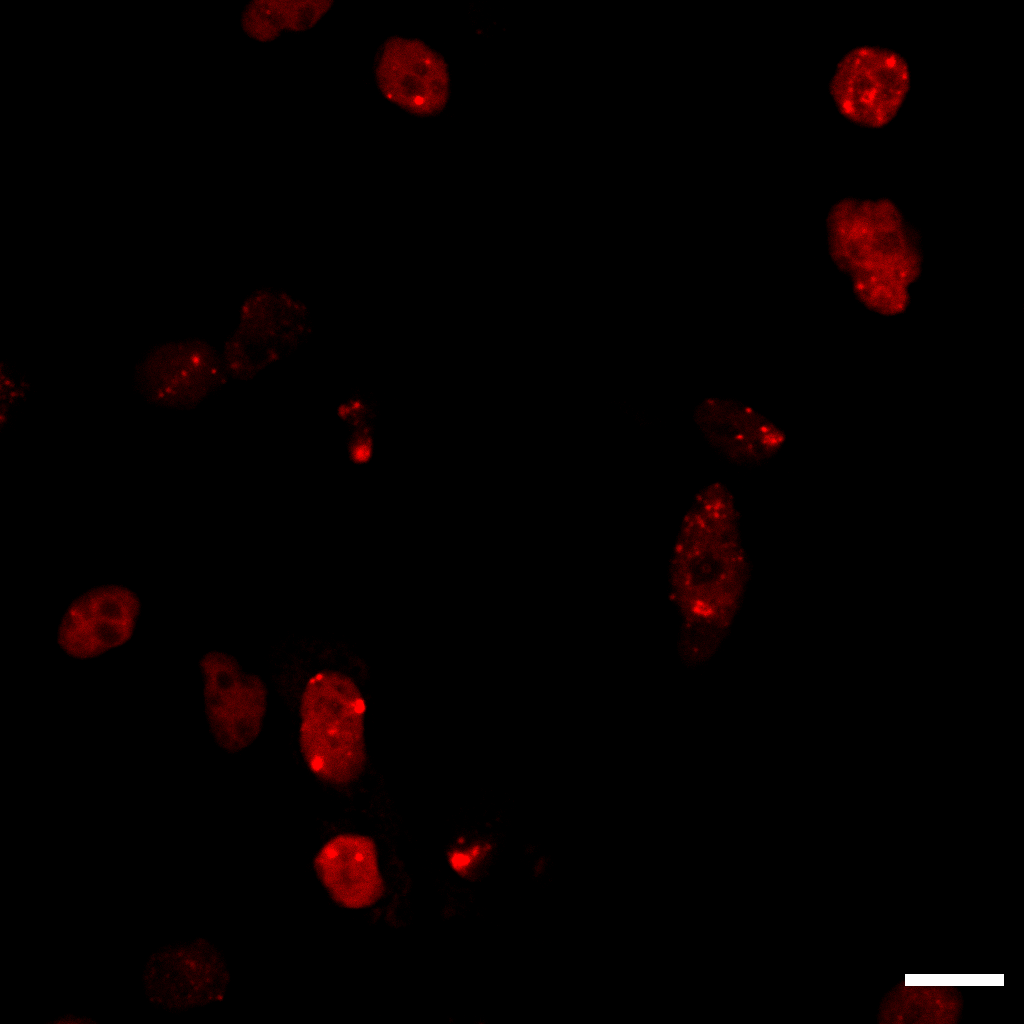

Supplement: Supplementary file 9 — EV Figures Source Data [file 44318_2024_58_MOESM9_ESM.zip › 2 EV Figures/EV3/EV3C/R51E-UV/C1-Composite.png]

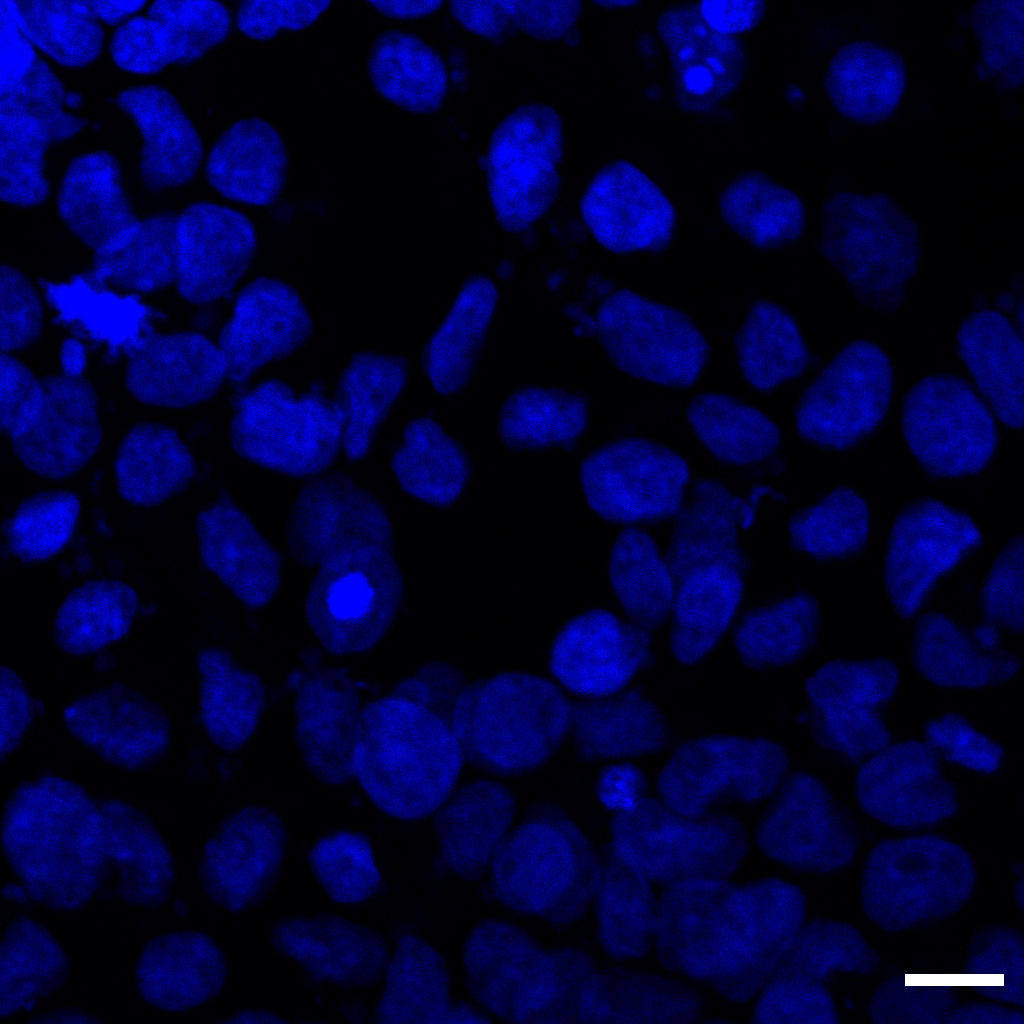

Supplement: Supplementary file 9 — EV Figures Source Data [file 44318_2024_58_MOESM9_ESM.zip › 2 EV Figures/EV3/EV3C/R51E-UV/C2-Composite.png]

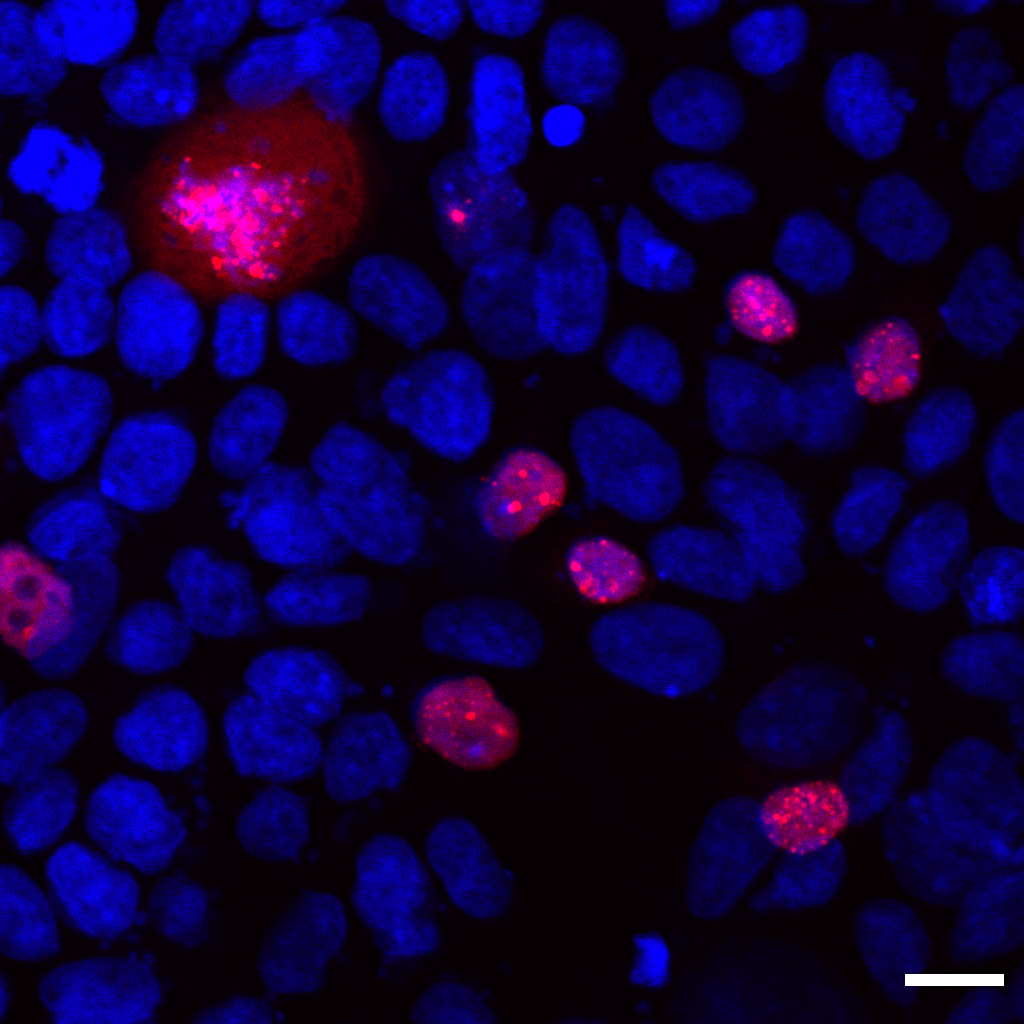

Supplement: Supplementary file 9 — EV Figures Source Data [file 44318_2024_58_MOESM9_ESM.zip › 2 EV Figures/EV3/EV3C/Rad18 -UV/Composite.png]

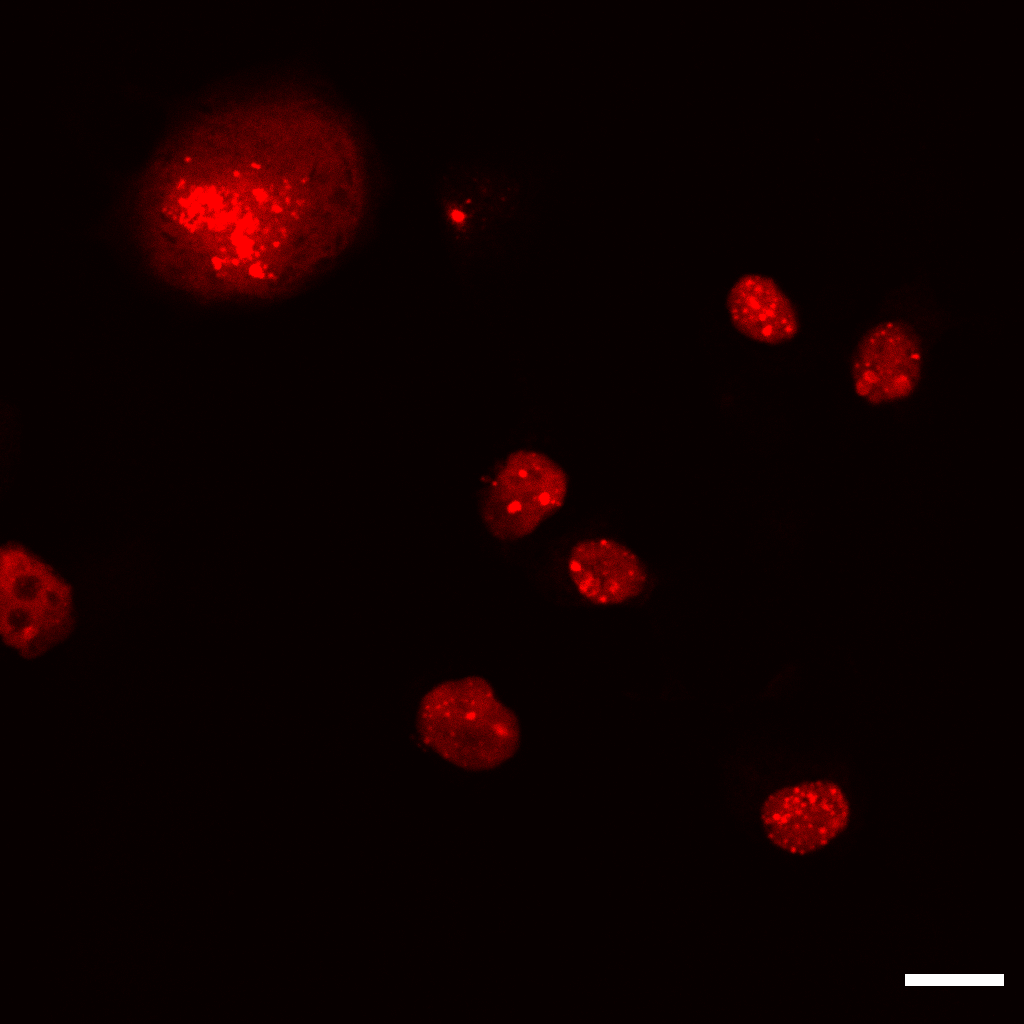

Supplement: Supplementary file 9 — EV Figures Source Data [file 44318_2024_58_MOESM9_ESM.zip › 2 EV Figures/EV3/EV3C/Rad18 -UV/C1-Composite.png]

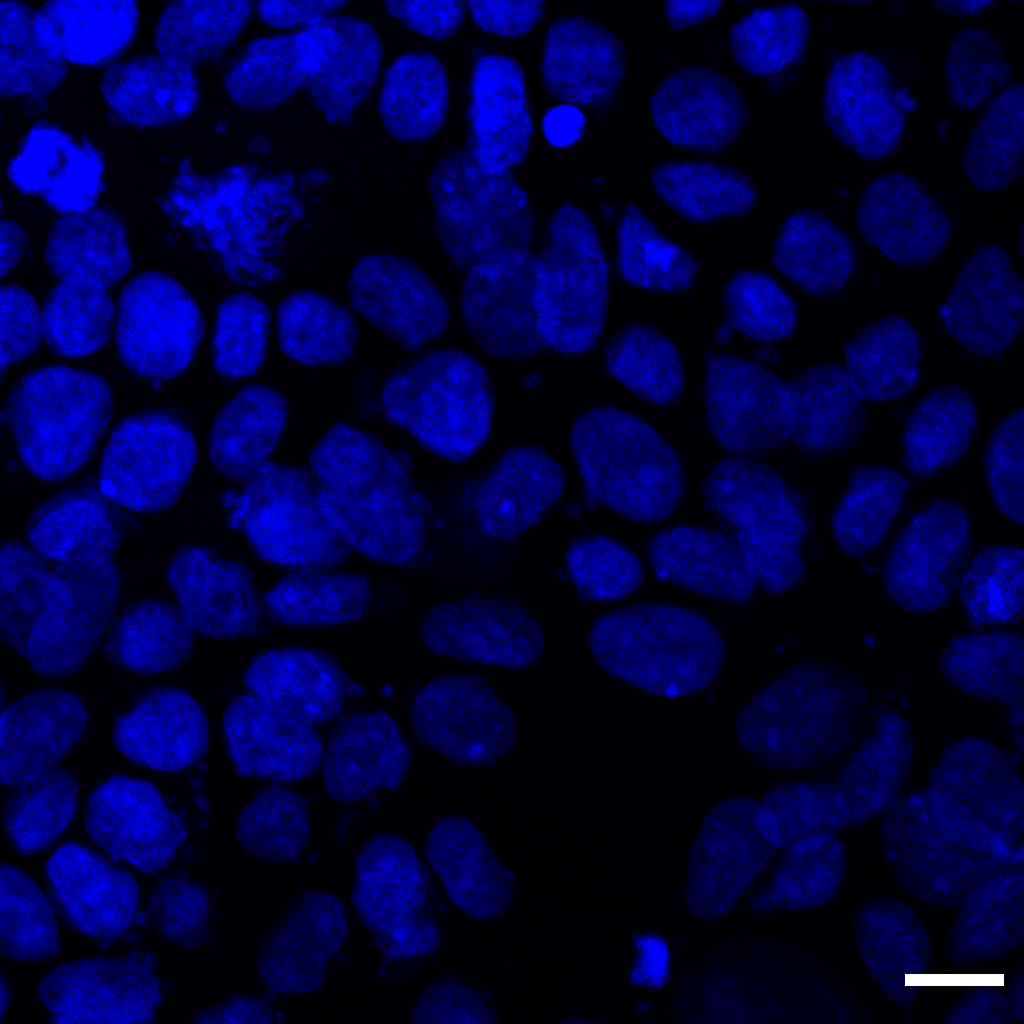

Supplement: Supplementary file 9 — EV Figures Source Data [file 44318_2024_58_MOESM9_ESM.zip › 2 EV Figures/EV3/EV3C/Rad18 -UV/C2-Composite.png]
