# Supplementary material for: Structural basis for RAD18 regulation by MAGEA4 and its implications for RING ubiquitin ligase binding by MAGE family proteins
Source: EMBO J. 2024 Mar 6;43(7):1273–300. doi: 10.1038/s44318-024-00058-9 (PMC10987633; doi:10.1038/s44318-024-00058-9)
Supplement: Supplementary file 6 — Source Data Fig. 3 [file 44318_2024_58_MOESM6_ESM.zip › Figure 3/3G/README.rtf]

The movies are provided for the MP data of RAD18/RAD6, RAD18/RAD6/MAGEA4 and the mass calibration. The report.csv files give additional information about the collection parameters for each condition.
