# Supplementary material for: Structural basis for RAD18 regulation by MAGEA4 and its implications for RING ubiquitin ligase binding by MAGE family proteins
Source: EMBO J. 2024 Mar 6;43(7):1273–300. doi: 10.1038/s44318-024-00058-9 (PMC10987633; doi:10.1038/s44318-024-00058-9)
Supplement: Supplementary file 8 — Source Data Fig. 5 [file 44318_2024_58_MOESM8_ESM.zip › Figure 5/5B/README.rtf]

I’ve included the colorimetric image of the Ub blot just to show where the marker is 
