# Supplementary material for: Structural basis for RAD18 regulation by MAGEA4 and its implications for RING ubiquitin ligase binding by MAGE family proteins
Source: EMBO J. 2024 Mar 6;43(7):1273–300. doi: 10.1038/s44318-024-00058-9 (PMC10987633; doi:10.1038/s44318-024-00058-9)
Supplement: Supplementary file 11 — Expanded View Figures [file 44318_2024_58_MOESM11_ESM.pdf]

Expanded View Figures

Figure EV1. MAGEA4 utilizes residues within the WH2 motif to bind the RAD18 R6BD.

(**A**) RAD18 R6BD/MAGEA4 AlphaFold2 model coloured by pLDDT score. (**B**) Predicted alignment error (PAE) plot of the RAD18 R6BD/MAGEA4 Alphafold model. (**C**) Size exclusion chromatography profiles of MAGEA4 WT and mutant proteins. (**D**) ITC analysis of the binding between MAGEA4 M161D and the RAD18 R6BD peptide. The ITC experiment was performed three times with similar results. (**E**) NMR analysis of MAGEA4/RAD18 R6BD peptide. Overlay of the ^{1}H-^{15}N TROSY-HSQC spectra of free [^{2}H,^{13}C,^{15}N] MAGEA4-MHD (red) and in a 3:1 molar ratio complex with unlabelled RAD18-R6BD (blue). Significant chemical shift perturbations are indicated with an arrow and observed in residues from the WH-B motif. To optimize data acquisition, a reduced ^{15}N spectral width was used, leading to folding of some peaks which are boxed. Source data are available online for this figure.

© The Author(s)

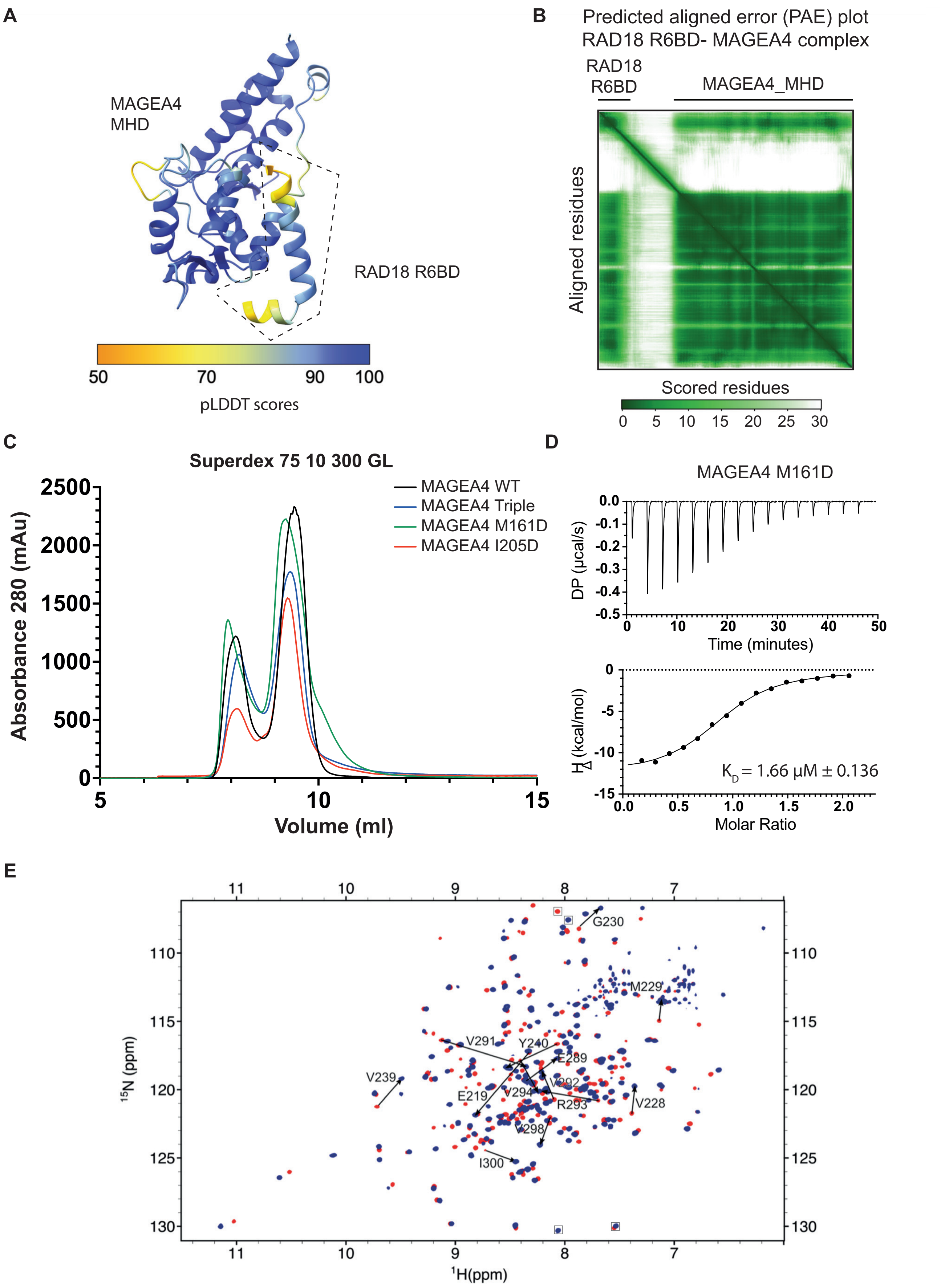

A
MAGEA4
MHD
RAD18 R6BD
50
70
90
100
pLDDT scores
B
Predicted aligned error (PAE) plot
RAD18 R6BD- MAGEA4 complex
RAD18
R6BD
MAGEA4_MHD
Aligned residues
Scored residues
0 5 10 15 20 25 30
C
Superdex 75 10 300 GL
MAGEA4 WT
MAGEA4 Triple
MAGEA4 M161D
MAGEA4 I205D
Absorbance 280 (mAu)
Volume (ml)
D
MAGEA4 M161D
DP (μcal/s)
Time (minutes)
ΔH (kcal/mol)
K$_D$ = 1.66 μM ± 0.136
Molar Ratio
E
G230
M229
V291
Y240
E289
V239
V292
E219
V294
R293
V228
V298
I300
^{15}N (ppm)
^{1}H(ppm)

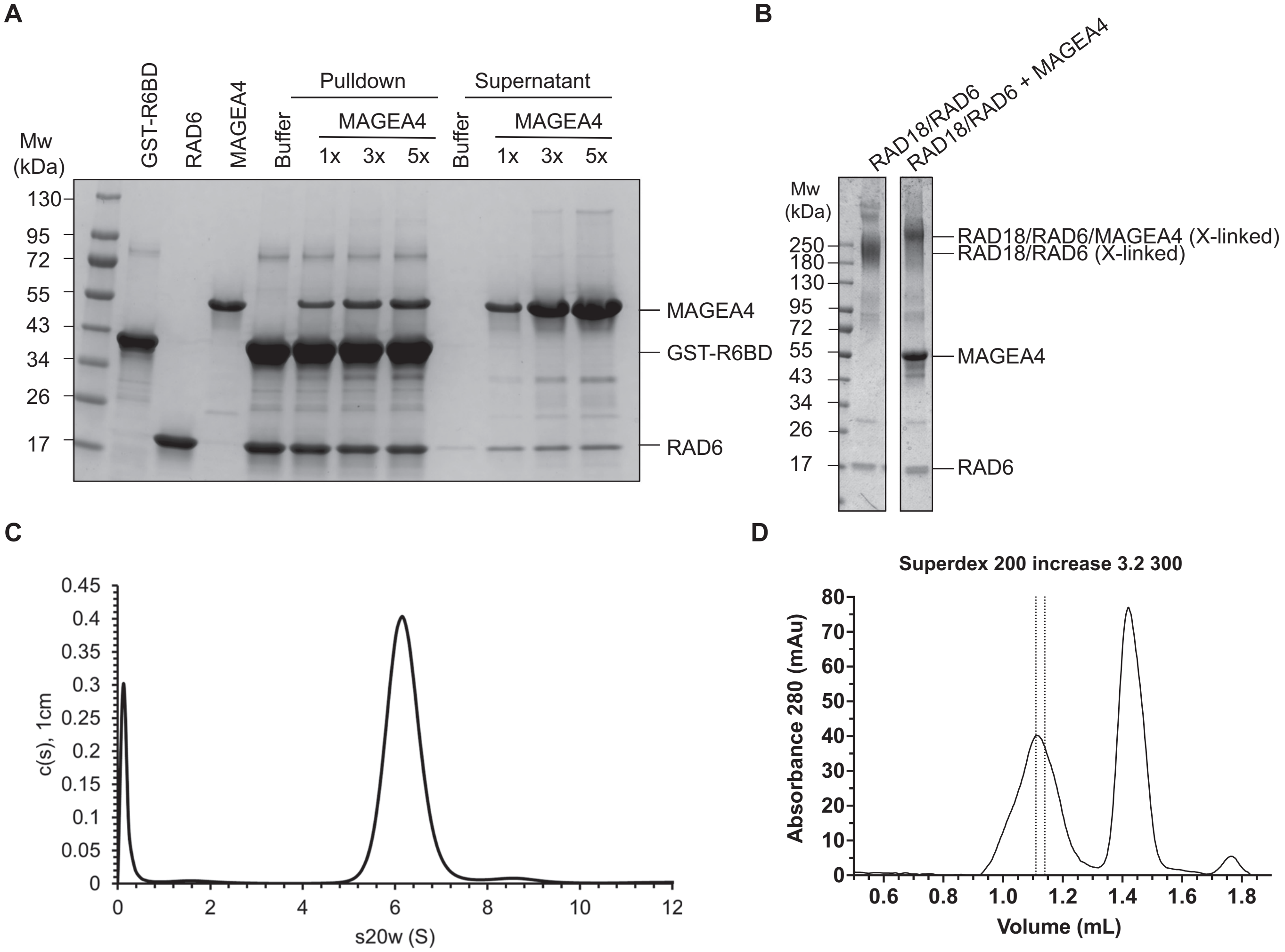

Figure EV2. Examining the impact of MAGEA4 binding on the RAD18/RAD6 complex.

(**A**) GST-tagged RAD18 R6BD was loaded onto glutathione beads and incubated with a 3x excess of RAD6. The immobilized GST-RAD18 R6BD was then incubated with an increasing concentration of MAGEA4 WT. The supernatant, containing proteins not bound to the GST-RAD18 R6BD, and the pulldown, containing proteins bound to the GST-RAD18 R6BD, from each condition were loaded onto an SDS PAGE gel and subjected to Coomassie staining to monitor RAD6 dissociation from the RAD18 R6BD. (**B**) Coomassie gel of RAD18/RAD6 cross-linked in the presence or absence of 5x molar MAGEA4. (**C**) Analytical ultracentrifugation of the RAD18/RAD6 complex. A sedimentation coefficient s20w of 6.2 S was obtained, corresponding to a molecular weight of 131.7 kDa. (**D**) Size exclusion chromatography profile of RAD18/RAD6/MAGEA4. The complex was first subjected to BS3 cross-linking, followed by size exclusion chromatography. The peak fraction was taken for analysis by Mass Photometry. Source data are available online for this figure.

© The Author(s)

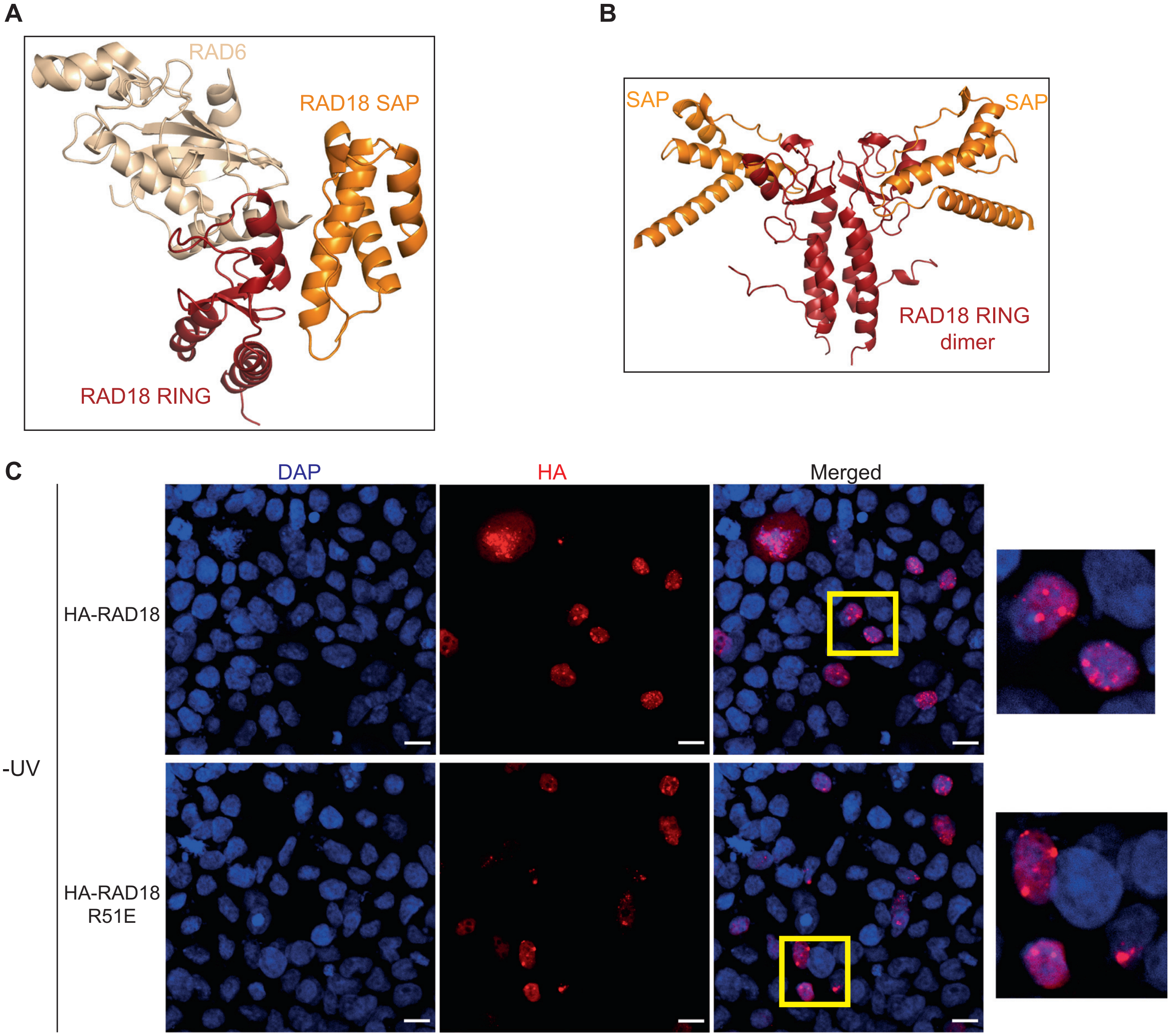

Figure EV3. Structural analysis of the interaction between the RING and SAP domains of RAD18.

(**A**) Model of the RAD18-RING/RAD6/SAP complex. RAD18 RING-SAP model is as shown in Fig. 4B. The RAD18 RING-RAD6 model was created by superposing each molecule on the respective RING (RNF12) and E2 (Ube2e2) of another RING-E2 (PDB: 6W9A). (**B**) The RING domain of the RAD18 RING/SAP Alphafold model is superposed onto the RING domain of the RAD18 RING domain dimer structure (2Y43). This resulted in the model of a RAD18 dimer containing two RING and two SAP domains without any clashes. (**C**) HEK293T cells were transfected with WT or R51E HA-RAD18. HA-RAD18 was immunostained using anti-HA antibody and the nucleus was stained using DAPI. Scale bar 12 μm. Source data are available online for this figure.

© The Author(s)

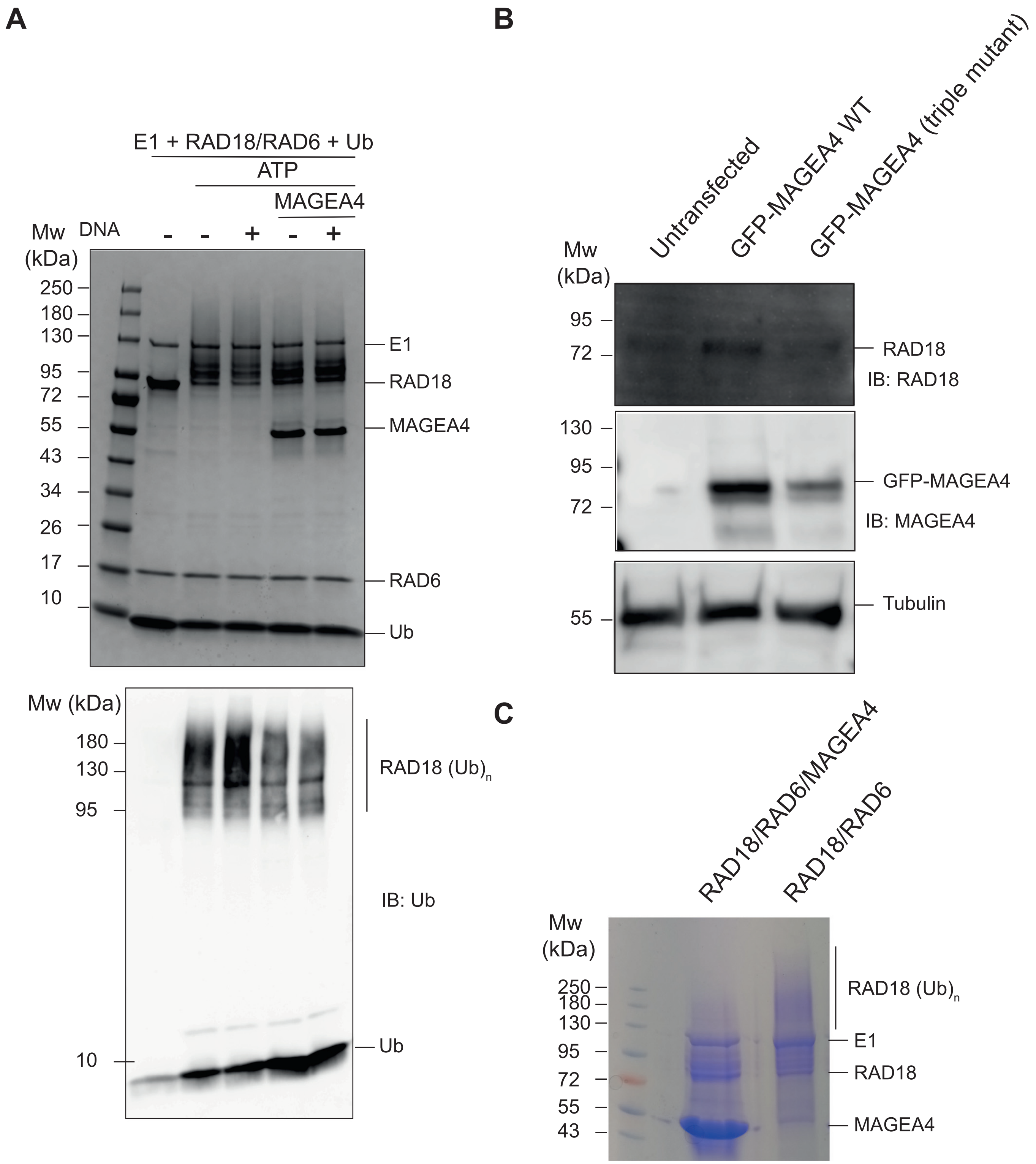
A
E1 + RAD18/RAD6 + Ub
ATP
MAGEA4
Mw DNA - - + - +
(kDa)
250
180
130
95
72
55
43
34
26
17
10
E1
RAD18
MAGEA4
RAD6
Ub
Mw (kDa)
180
130
95
10
RAD18 (Ub)n
IB: Ub
Ub
B
Untransfected
GFP-MAGEA4 WT
GFP-MAGEA4 (triple mutant)
Mw
(kDa)
95
72
RAD18
IB: RAD18
130
95
72
GFP-MAGEA4
IB: MAGEA4
55
Tubulin
C
RAD18/RAD6/MAGEA4
RAD18/RAD6
Mw
(kDa)
250
180
130
95
72
55
43
RAD18 (Ub)n
E1
RAD18
MAGEA4

 © The Author(s)

Figure EV4. MAGEA4 protects RAD18 from degradative autoubiquitination regardless of DNA.

(**A**) In vitro ubiquitination reactions performed with the RAD18/RAD6 complex in the presence or absence of a 33 bp single-stranded DNA, with or without a 3x excess of MAGEA4, as indicated. Reactions were subjected to SDS PAGE, followed by Coomassie staining (top) or WB using an antibody against ubiquitin (bottom). (**B**) GFP-tagged MAGEA4 WT or MAGEA4 triple mutant were transfected into HEK293T cells and the levels of endogenous RAD18 were monitored. The soluble fraction was subjected to SDS PAGE and WB using antibodies against RAD18 or GFP. Tubulin levels were monitored as a loading control. (**C**) In vitro ubiquitination reactions were performed using the RAD18/RAD6 complex in the presence or absence of MAGEA4. The reaction mix was analysed using SDS-PAGE followed by Coomassie staining. Autoubiquitinated RAD18 in the RAD18/RAD6 sample, as indicated in the figure, was analysed using mass spectrometry to identify ubiquitination sites. Source data are available online for this figure.

© The Author(s)

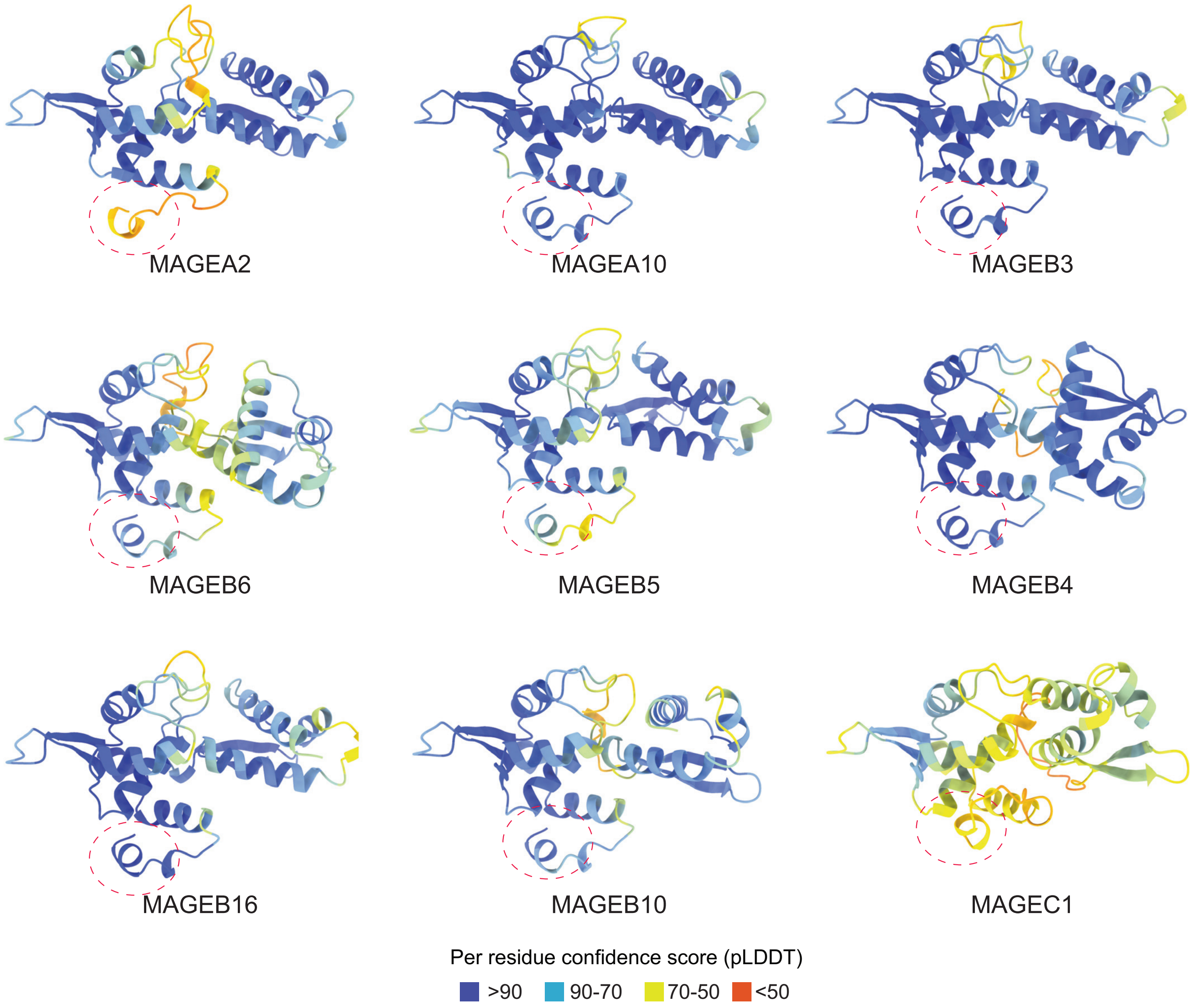

Figure EV5. AlphaFold predictions of various Type 1 MAGEs, coloured by pLDDT score.

The red circle marks the peptide binding groove of various MAGE proteins.

 © The Author(s)
